# Mood Disorder Detection in Adolescents by Classification Trees, Random Forests and XGBoost in Presence of Missing Data

**DOI:** 10.3390/e23091210

**Published:** 2021-09-14

**Authors:** Elzbieta Turska, Szymon Jurga, Jaroslaw Piskorski

**Affiliations:** 1 Institute of Pedagogy, University of Zielona Gora, 65-417 Zielona Gora, Poland; e.turska@wpps.uz.zgora.pl; 2 Department of Neurology, Collegium Medicum, University Hospital, University of Zielona Góra, 65-417 Zielona Gora, Poland; s.jurga@cm.uz.zgora.pl; 3 Institute of Physics, University of Zielona Gora, 65-417 Zielona Gora, Poland

**Keywords:** classification, classification trees, rpart algorithm, random forests, XGBoost, mood disorders, emotions detection, Burns test

## Abstract

We apply tree-based classification algorithms, namely the classification trees, with the use of the rpart algorithm, random forests and XGBoost methods to detect mood disorder in a group of 2508 lower secondary school students. The dataset presents many challenges, the most important of which is many missing data as well as the being heavily unbalanced (there are few severe mood disorder cases). We find that all algorithms are specific, but only the rpart algorithm is sensitive; i.e., it is able to detect cases of real cases mood disorder. The conclusion of this paper is that this is caused by the fact that the rpart algorithm uses the surrogate variables to handle missing data. The most important social-studies-related result is that the adolescents’ relationships with their parents are the single most important factor in developing mood disorders—far more important than other factors, such as the socio-economic status or school success.

## 1. Introduction

Adolescence is one of the most difficult periods in a person’s life. It is also one of the most consequential ones—what happens during this period may and often will shape the life of a person.

An adolescent transitions between childhood an adulthood, and this is one of the formative developmental tasks that everyone goes through. The young person, not being unambiguously identified by their immediate environment as an adult or a child, may experience uncertainty, loss of orientation, internal and external conflicts, and tensions. These lead to changes in mood and behavior (compare [1] and references therein). These developmental tasks are made even more difficult by the fact that the adolescents need to cope with violent neurohormonal changes [2].

For these reasons, many adolescents experience mood disorders, which include sadness, dejection, hopelessness, sense of worthlessness, and losing interest in various activities and social relationships [1,2]. These feelings are not exclusive to adolescents as everyone can experience them at various points in their life, but they seem to be particularly prevalent in this group [2]. It is necessary to stress that mood disorders as described in this paper, as well as those which are being cited, should not be equated with depression, which is a pathological form and can only be diagnosed by a qualified psychiatrist. What we are concerned about is a state of depressed mood, which is quite distinct from depressive syndromes and clinical depression [2].

Depressed moods are experienced by 15–40% of adolescents, but in most of them the mood disorders resolve and disappear. However, in a small fraction they develop into the depressive syndrome (5–6%), and for some it develops into clinical depression (1–3% of adolescents with mood disorders) [2].

According to Carr et al. [3] the development of mood disorders is mainly determined by the following factors

1. Genetic susceptibility, the presence of mood disorders in the family.
2. Early loss experience related with health problems or psychological loss (parting, orphaning, stay at orphanage, deleterious social conditions, loss of trust in relationships following mistreatment).
3. Difficulties in contacts with parents, leading to the loss of the sense of security. This factor also comprises other forms of family dysfunctionality, which lead to losing the sense of security and mood disorders, for example: depression in parents, domestic violence, alcohol or drug abuse, and family disintegration.
4. Individual personality traits, which include low intelligence, hampering expected school achievement, feeling of helplessness, and as excessive self-criticism following from the conviction that the most important sources of support are unavailable to the child.

The literature cited above, as well as analyses and classifications proposed in other sources, point to family relation as one of the most important factors in mood disorders [4,5,6].

One of the most often used tools for establishing and classifying mood disorders is the Burns Depression Checklist [7]. This is a self-assessment-based checklist in which the respondent assess their own mood based on the analysis of their thoughts and feelings, relationships with other people, the actions they undertook, physical symptoms, and suicidal impulses (Burns, 2008). The answers are used to establish the presence of depressed mood as well as categorizing it according to the following scale (we are using a slightly simplified list proposed in [1]).

1. No depressed mood.
2. Normal state but there is no joy.
3. Mildly depressed mood.
4. Average level of depressed mood.
5. Strongly depressed mood.
6. Very strongly depressed mood.

### 1.1. Motivation for the Present Study

In 2012, a major study called “The Life of the Citizens of Lubusz Voivodeship. The Present and Future Outlook” was carried out by the University of Zielona Gora, Poland. It was geared towards getting to know many aspects of the life of western Poland, one of which was the condition of the secondary school students, i.e., adolescents, from the Lubusz voivodeship. This part of this study gave a large questionnaire to 2508 students, aged 13–16 years old, and one of its parts was the Burns checklist. In the returned questionnaires, there were 221 cases for which the mood disorder could not be established because the data necessary to do so were missing. There were at least some answers to other questions, but not to the specific ones, given by the Burns checklist [8].

This was not the only problem with this study. As the reader may learn from the supplementary resources accompanying the present paper, which contain all the questions used in this questionnaire, many of the questions are repetitive and highly correlated, some of them might be considered misleading (in the opinion of the authors of this paper), some of them lack the necessary options for respondents (i.e., the specific answer a student may want to give is absent and cannot be selected), and the questionnaire is very long, making it difficult for teenagers to concentrate for long enough and fill out the whole document. These flaws have led to many missing answers, which entails the impossibility to use standard statistical methods to establish the correlates of mood disorder. Indeed, there are few publications following this study, which might be surprising given how large it was.

Classification trees and related techniques (i.e., random forests and the XGBoost algorithm) are considered to be robust to the problems identified above. They can deal with missing data either naturally (classification trees via the rpart algorithm) or by cleverly inputting them (random forests and XGBoost). They can also naturally deal with large number of covariates, which is true of the questionnaire being described. Additionally, the classification trees are highly interpretable and can help identify the most important variables for prediction, effectively ranking them from the most to the least important one [9,10,11].

Thus, the aims of the present paper are the following. The first goal is to try and apply the three algorithms to see if they can cope with the missing values in the data and to what extent their absence influences their predictive power. The measures used to establish this are the mean misclassification error and the confusion matrix. We also aimed to build a predictive model helping us to classify the non-respondents (i.e., those for which the Burns test could not be used) as either having the more severe cases of mood disorder or not. These models cannot have any application outside this study since it is hard to expect that someone would use all the other question but the Burns checklist. Rather, it is a proof of concept that these types of techniques can be used for highly problematic, questionnaire-based datasets. Another aim was to try and identify the correlates of mood disorder, which turns out to be almost impossible to do using the standard statistical methods.

### 1.2. Classification Trees

Classification trees are one of the oldest and most often used ML techniques. Because of their simplicity, both conceptual and algorithmic, they are among the most popular approaches—they were found to be among the top 10 algorithms in data mining [12]. They are used in a wide variety of applications, ranging from stochastic digit recognition to disease severity classification [9,10,13].

Let us define

(1) $$X\overset{def}{=}\left\{X_{1},X_{2},\ldots ,X_{p}\right\},$$

as the predictor space. The classification tree partitions this space into *J* distinct, non overlapping regions

(2) $$R_{1},R_{2},\ldots ,R_{J}.$$

The regions $R_{i}$ are high-dimensional rectangles (boxes).

Let us assume that the sample population consists of *n* observations from *C* classes. A trained classification tree model partitions the observations into *J* terminal groups, to each of which one of the *C* classes will be assigned on the basis of the majority class obtained during the training process on the training set (we assume no ties).

The goal of the training procedure is finding the boxes $R_{1},\ldots ,R_{J}$ with the use of a top-down, greedy procedure called recursive binary splitting. The procedure begins with an *R* that is composed of all observations, and then this region is successively split into two. The splits are based in minimizing an *impurity index*, which measures classification error.

Before we go over the most important impurity indices, let us define an auxiliary concept. In classification problems, sometimes the consequences of misclassifying observations to some classes are more important than to other classes. This problem is tackled by defining the so-called *loss matrix*, which is a C×C matrix with L(i,j) being the loss incurred by classifying a class *i* observation as class *j* [9,10,13].

#### 1.2.1. Splitting Criteria

Let us assume we are splitting a node *A* into a left and a right node—$A_{L}$ and $A_{R}$, respectively.

The decision on how to split the *A* node is usually based on look-ahead rules or defining an impurity function. Since the former is computationally expensive, it is not very often used, and the implementation used in the present paper employs the latter approach.

Let us define an impurity function as follows

(3) $$I\left(A\right)=\sum_{i=1}^{C}f\left(p_{iA}\right),$$

where $p_{iA}$ is the proportion of the training samples in node *A* that belong to class *i*. We impose the condition that I(A)=0 if *A* is pure, so function *f* has to be concave with

f(0)=f(1)=0.

The most popular node impurity indices meeting these criteria are  [9,10,13]:

1. Classification error rate

f(p)=1−max(p),

This measure is rarely used because it lacks sensitivity [10].

2. The Gini index

f(p)=p(1−p),

3. Entropy (information index)

f(p)=−plog(p),

in fact, the Gini index and Entropy are very similar to one another.

The split of *A* into $A_{L}$ and $A_{R}$ is chosen so that it maximizes the following quantity

(4) $$\Delta I=p\left(A\right)I\left(A\right)-p\left(A_{L}\right)I\left(A_{L}\right)-p\left(A_{R}\right)I\left(A_{R}\right).$$

ΔI is called impurity reduction [13].

#### 1.2.2. Incorporating Losses through the Loss Matrix

One of the methods in which the risk of misclassification is reduced that is not covered by the impurity measures described above is incorporating the loss matrix.

Let us assume that samples are randomly distributed among the *C* classes with probabilities $\left(p_{1},p_{2},\ldots ,p_{c}\right)$.

1. Generalized Gini index: The Gini index follows from the misclassification probability [13]

(5) $$G=\sum_{i=1}^{C}\sum_{j=1,j\neq i}^{C}p_{i}p_{j}=\sum_{i}p_{i}\left(1-p_{i}^{2}\right).$$

*G* measures total variance across the *C* classes. If L(i,j) is the (i,j) misclassification loss, the expected cost of misclassification is $\sum_{i}\sum_{j}L\left(i,j\right)p_{i}p_{j}$, which leads to the *generalized Gini index* [13]

(6) $$G\left(p\right)=\sum_{i}\sum_{j}L\left(i,j\right)p_{i}p_{j}.$$

2. Altered priors method The software used in this paper uses the altered priors method, so let us concentrate on this approach. Let us define

(7) $$R\left(A\right)=\sum_{i=1}^{C}p\left(i|A\right)L\left(i,\tau A\right),$$

where *A* is a node of the classification tree, p(i|A) is the conditional probability that the true class of observation *x* is *i* on the condition that observation *x* belongs to class *A*, τ(A) is the class assigned to *A*, and R(A) is the risk of *A*, i.e., the proportion of misclassified cases [13].

It can be shown that

(8) $$R\left(A\right)=\sum_{i=1}^{C}\pi_{i}L\left(i,\tau \left(A\right)\right)\frac{n_{iA}}{n_{i}}\frac{n}{n_{A}},$$

where L(i,τ(A)) is the loss matrix for incorrectly classifying an *i* as τ(A), $n_{i}$ is the number of observations in the sample that are class *i*, $n_{A}$ is the number observations in node *A*, and  $n_{iA}$ is the number of observations of class *i* in node *A*.

Let us define $\pi_{i}$ as the prior probability of class *i*. Let us assume that there exist $\tilde{\pi_{i}}$ and $\tilde{L}$ such that

(9) $$L\left(i,j\right)=\left\{\begin{array}{cc} L_{i} & i\neq j\\ 0 & i=j, \end{array}\right.$$

so that now

(10) $$\tilde{\pi_{i}}=\frac{\pi_{i}L_{i}}{\sum_{j}\pi_{j}L_{j}}.$$

For C=2, the above is always possible and other priors are exact, and for arbitrary loss matrix dimension of C>2 the above formula is used with [13]

$$L_{i}=\sum_{j}L\left(i,j\right).$$

When a tree is being built, one of the above measures is used to evaluate the quality of a particular split. A split is performed if it leads to increased node purity [9,10].

#### 1.2.3. Tree Pruning

The methodology described above will almost certainly lead to growing very large trees and consequently overfitting the data. To avoid this, the concept of tree pruning has been introduced [9]. This strategy is based on growing a very large tree and then pruning it back based on selecting a subtree that leads to the lowest classification error established by the cross-validation procedure. One of the best known algorithms to achieve this goal of the most effective subtree (pruned tree) is cost complexity pruning, also known as the weakest link pruning [9,10].

In cost complexity pruning, a series of trees $T_{0}\ldots T_{m}$ is created, with $T_{0}$ being the initial, full size tree and $T_{m}$ being the tr. A tree is created at step *i* by removing a subtree from the previous tree (i−1) and replacing this whole subtree by a leaf node containing the value arrived at during the tree building process. The tree to be removed is chosen in the following way  [14,15]:

1. The error rate of tree *T* over dataset *S* is defined as err(T,S),
2. The subtree for which
  $$\frac{\mathrm{err}(\mathrm{prune}(T,t),S)-\mathrm{err}(T,S)}{\left|\mathrm{leaves}(T)|-|\mathrm{leaves}(\mathrm{prune}(T,t))\right|},$$
  is minimal is removed. Function prune(T,t) is the tree defined by pruning the *t* subtrees from the *T* tree.

### 1.3. Random Forests

Random forests is an algorithm based on the bagging algorithm  [16]. In the bagging approach, the bootstrapping idea is utilized to reduce the variance of a machine learning method  [10]. It is not limited to the tree-based methods and has been used in many other contexts [9,10].

In this approach, *B* separate training sets are bootstrapped from the training set and the predictive functions

$$\left\{\hat{f_{1}^{*}}\left(x\right),\hat{f_{2}^{*}}\left(x\right),\ldots ,\hat{f_{B}^{*}}\left(x\right)\right\}$$

are built.

The final predictive function is an average of these:

(11) $${\hat{f}}_{\mathrm{avg}}\left(x\right)=\frac{1}{B}\sum_{b=1}^{B}{\hat{f}}^{*b}\left(x\right),$$

where the asterisk marks a function on a bootstrapped set  [9,16].

If we assume that in a limited classification problem with two classes each of the $f^{*b}$ can yield a result in {−1,1}, $\mathrm{sign}({\hat{f}}_{\mathrm{avg}}\left(x\right))$ will act as a *majority vote* on a bagged classification problem [9,16].

The error in this approach can be estimated as out of bag error; i.e., the error is measured for the observations that did not enter the currently bootstrapped sample [9,10,16].

*Random forests* are an improvement over bagged trees by introducing trees *decorrelation*. Decorrelation is introduced by choosing a random sample of *m* predictors from the full set of *p* predictors at each split. This split can thus only use the selected *m* predictors, and at each split a new random sample of *m* predictors is taken. It has been reported that random forests lead to a substantial improvement in prediction over both standard trees and bagging [10].

### 1.4. Tree Boosting

Boosting is a method proposed by Kearns and Valiant  [17,18] that introduces the idea of many weak classifiers helping to produce a strong classifier.

A weak classifier is a classifier whose error only marginally outperforms random guessing. In boosting, such weak classifiers are repeatedly (sequentially) applied to progressively modified data.

Let us assume that we have a two-class problem with output in the set Y∈{−1,1}. Using the predictor space (1), a  classifier G(X) produces a prediction with the following error rate

(12) $$Err.=\frac{1}{N}\sum_{1}^{N}I\left(y_{i}\neq G\left(x_{i}\right)\right),$$

where *I* is an indicator function [9,18].

The boosting algorithm produces a sequence of *M* weak classifiers $G_{m}\left(x\right)$ where m=1,2,…M.

While this approach can bee applied to many ML algorithms, we are interested in applying it to classification trees.

Let us assume the prediction space is divided into *C* disjoint regions (terminal nodes) $R_{j}$ where j=1…,C (compare (2)). A  constant γ is assigned to each such region with the following predictive rule [9,18]

(13) $$x\in R_{i}\Rightarrow f\left(x\right)=\gamma_{i}.$$

We now express a tree as

(14) $$T\left(x;\Theta \right)=\sum_{i=1}^{C}\gamma_{i}I\left(x\in R_{i}\right),$$

with parameters $\Theta \in {\left\{R_{i},\gamma_{i}\right\}}_{1}^{C}$. These parameters are found by minimizing the empirical risk

(15) $$\hat{\Theta }=\arg\min_{\Theta }\sum_{i=1}^{C}\sum_{x_{i}\in R_{i}}L\left(y_{i},\gamma_{i}\right).$$

In the previous section, the Gini index was introduced to replace the misclassification loss. The boosted version of trees built with the use of the Gini index is a sum introduced in a forward manner [9]

(16) $$f_{M}\left(x\right)=\sum_{m=1}^{M}T\left(x;\Theta_{m}\right).$$

Each step of this procedure must optimize the following problem

(17) $${\hat{\Theta }}_{m}=\arg\min_{\Theta_{m}}\sum_{i=1}^{N}L\left(y_{i},f_{m-1}\left(x_{i}\right)+T\left(x_{i};\Theta_{m}\right)\right),$$

for the region set and constants

$$\Theta_{m}={\left\{R_{im},\gamma_{im}\right\}}_{1}^{Cm},$$

of the next tree given the current model’s $f_{m-1}\left(x\right)$ [9].

Thus, we have arrived at a *numerical* optimization problem. This problem can be solved by many numerical algorithms—in this paper, we have applied the *extreme gradient boosting* algorithm, which is an implementation of the algorithm proposed in [19,20].

### 1.5. Handling Missing Values

As will become apparent from the Materials and Methods section, missing values handling is one of the most important aspects of any analysis of the data presented in this paper.

In most approaches, missing values are removed from the dataset; however, in our case, for the reasons described in the Materials and Methods section, this would mean tossing almost all our data. Additionally, as we describe in the Discussion section, we believe that the absence of data is meaningful.

We will use two methods according to their availability in the different algorithms introduced above.

#### 1.5.1. Imputation

Data imputation is a concept that dates back to classical statistics. It is the process of dealing with missing data by substituting those data with a plausible value. This process usually entails some sort of inference about both the value and the plausibility of the data [21].

The most popular methods of imputations include replacing the missing data with the mean or the median or modeling the data on the basis of the existing data [21,22].

In the spirit of ML research we have chosen a method based on modeling the missing data. For the reasons described in the Introduction as well as in Materials and Methods, we could not use any statistical methods. Rather, we have used the typical ML approach of imputing missing values with the use of the proximity matrix calculated by the random forest algorithm [23].

The algorithm begins by replacing the missing values by medians. Then, the random forest algorithm is run on this data and the proximity matrix is calculated. In the next step, this proximity matrix is used to update the imputed missing values. The procedure is repeated (iterated) a set number of times. This approach has aspects of both single imputation  [24] and building a model, for example, a regression model [24,25], known from other areas of statistics and machine learning. This approach starts by single imputation for each missing value using the median, followed by building successive forests, modifying the missing data, tracking their performance of the model, and arriving at optimal values for the missing data.

It is worth noting that there are some reservations about the accuracy of this approach [16]. Our exact implementation can be found in the supplementary materials.

#### 1.5.2. Surrogate Variables

This method is characteristic of the rpart algorithm. In this algorithm, any observation that has values for the dependent variable and at least one value for the independent variable will be taken into account in both training and prediction.

In this approach the impurity reduction is still the objective; i.e., expression (4) is still being maximized. However, the interpretations of the last two elements of this expression are modified. First of all, the impurity indices $I(A_{R})$ and $I(A_{L})$ are limited only to those observations for which a particular split candidate variable is available. Additionally, probabilities $p(A_{L})$ and $p(A_{R})$ are modified to be defined only for the observations which are not missing the values for the particular variable, as well as adjusting them so that they sum to the value of p(A) for the parent node [13].

The next step is dealing with the variables that are actually missing variables. Once the primary splits have been found as explained above, a list of surrogate predictors and split points is formed. The surrogate predictors and corresponding split points are ordered according to their ability to emulate the primary split in the training data. Surrogates are then ranked according to the misclassification risk and used when going down the tree  [9,13].

The surrogate predictor method uses the correlations between the predictors, the assumption being that the higher the correlation between the missing predictor and the other predictors, the smaller loss of information will be incurred as a result of the missing values [13].

## 2. Materials and Methods

A questionnaire concerning various aspects of the live of a gymnasium (in Poland this is lower secondary school) student was given to 2508 students. The questions preparation process was not systematic—rather it involved researchers in various subfields of education research preparing questions that were of interest to them.

### 2.1. Data Preparation

There were 53 questions in total, but most of them included subquestions. All of them can be found in the social_AI.Rmd Rmd document available in the supplementary materials. Question 21 consisted of subquestions constituting the Burns checklist test. They were used to classify the students professional to one of the six groups given in the Introduction. This question, naturally, was not used in training the predictive models–it was only used by the researchers carrying out the study to establish the response variable.

The numbers of respondents in the respective mood disorder classes identified in the study were the following.

1. no depressed mood—435 cases,
2. normal state but there is no joy—319 cases,
3. mildly depressed mood—804 cases,
4. average level of depressed mood—577 cases,
5. strongly depressed mood—123 cases,
6. very strongly depressed mood—28 cases.

It is clear that the dataset is heavily unbalanced, with very few cases in the more severe mood disorder categories. There were 221 respondents who could not be classified due to missing some answers to the subquestions of this question. Since there were very few students in classes 5 and 6 we grouped all respondents into just two classes, i.e., those in which a severe mood disorder was present (classes 5 and 6) and those in which it was absent (classes 1 through 4). This classification was the target variable of the present study, i.e., we set out to build a model that classifies the students into those two classes, without using question 21.

All the questions that had a natural ordering (e.g., the answers could be classified on a scale) were treated as numerical variables. The questions that did not have this kind of ordering were coded with the one-hot encoding method [9]. Open-ended questions that could not be cast in a numerical form were dropped.

There were a substantial number of answers missing. We believe that there were various reasons for this absence, some of which have been addressed in the Discussion section below. The statistics about these absences are given in the social_AI.html file available in the supplementary materials file. Two questions, 11 and 12, contained too many missing values and therefore were dropped from the analysis.

After encoding different subquestions separately, one-hot encoding as well as removing open-ended, Burns checklist-related, and too incomplete questions, we arrived at 128 variables.

All the details about questions and encoding the variables can be found in the supplementary materials file.

### 2.2. Software and Methods

In the present paper we have used the libraries available for the R programming language. For classification trees we used the Rpart package [26], for random forests we used the randomForest package [27], and for the XGBoost method we used the xgboost package [28].

The programming framework for the analysis was provided by the mlr package [29].

### 2.3. Training and Test Data

The whole dataset was split into three parts: the first two were the training and test datasets, and the third was the data for which the mood variable could not be established. This dataset was only used for demonstration purposes of how the algorithm might work for new data.

The training dataset consisted of 1604 cases, which corresponds to roughly 70% of the whole number of the cases for which the mood could be established through the Burns checklist. The test dataset consisted of 683 cases, which corresponds to roughly 30% of the cases for which mood was available. We believe this split allows the algorithm described below enough data to ensure similarity of the two groups, at the same time providing enough data for training. Care has been taken to retain as much as possible of the relative distribution of the mood response variable throughout the training and test datasets. In other words, the subjects were selected at random, but the mood variable composition in each dataset corresponds to the distribution in the whole dataset. The function achieving this is available in the supplementary materials file for this paper.

### 2.4. Parameter Tuning and Models Training

For each of the models, we first tuned the hyperparameters through the 5-fold cross validation procedure, and then trained the models using the training data. Because the hyperparameter space was quite large, we used random search. In the following we are using the nomenclature provided by the mlr package. The hyperparameters tuned for the respective models were the following.

For the rpart algorithm, we have tuned the following hyperparameters (compare [11]):

1. minsplit—the minimum number of cases needed to split a node,
2. maxdepth—the maximum depth of the tree,
3. cp—complexity parameter calculated for each level of depth of the tree; if this parameter of a depth is less than the threshold, the nodes at this level will not be split further,
4. minbucket—minimum number of cases in a leaf,
5. maxsurrogate—number of the surrogates to retain in the model (if a case is missing a value, it is passed to successive surrogates)—this is a key hyperparameter for the purposes of the present paper.

For the random forest algorithms, we tuned the following hyperparameters (compare [11]):

1. ntree—the number of individual trees in the forest,
2. mtry—the number of features to randomly sample at each node,
3. nodesize—the minimum number of cases allowed in a leaf,
4. maxnodes—the maximum number of leaves allowed.

For the XGBoost algorithms, we tuned the following hyperparameters (compare [11]):

1. eta—the learning rate,
2. gamma—the minimum amount of splitting by which a node must improve the predictions,
3. max_depth—the maximum levels deep that each tree can be grown,
4. min_child_weight—the minimum degree of impurity needed in a node before a split is attempted,
5. subsample—the proportion of cases to be randomly sampled from each tree,
6. colsample_bytree—the proportion of predictor variables sampled for each tree,
7. nrounds—the number of sequentially built trees in the model,
8. eval_metric—the type of residual loss function to be used.

The details of the tuning procedure can be found in the Rmarkdown files for each model accompanying this paper.

### 2.5. Performance

The performance of the models was measured by the *mmce* (mean misclassification error) during training on the training set and also on the test set for the final models. The quality of the final models was assessed by the confusion matrix for the test set and also assessing how well the various mood disorder classes were identified by the model, even though the mood variable in our modes were cast to a dichotomous variable. We were interested in how well the models identified the most severe mood disorders.

## 3. Results

The models have been tuned on the training data and then tested on the test data. The tuning procedure and model training had the following results for the respective models.

### 3.1. Models Tuning

In this section, we present the results of model tuning obtained for the training data.

#### 3.1.1. Decision Trees via the Rpart Algorithm

The values of the tuned hyperparameters obtained via the cross validation procedure are presented in Table 1.

**Figure 1:**
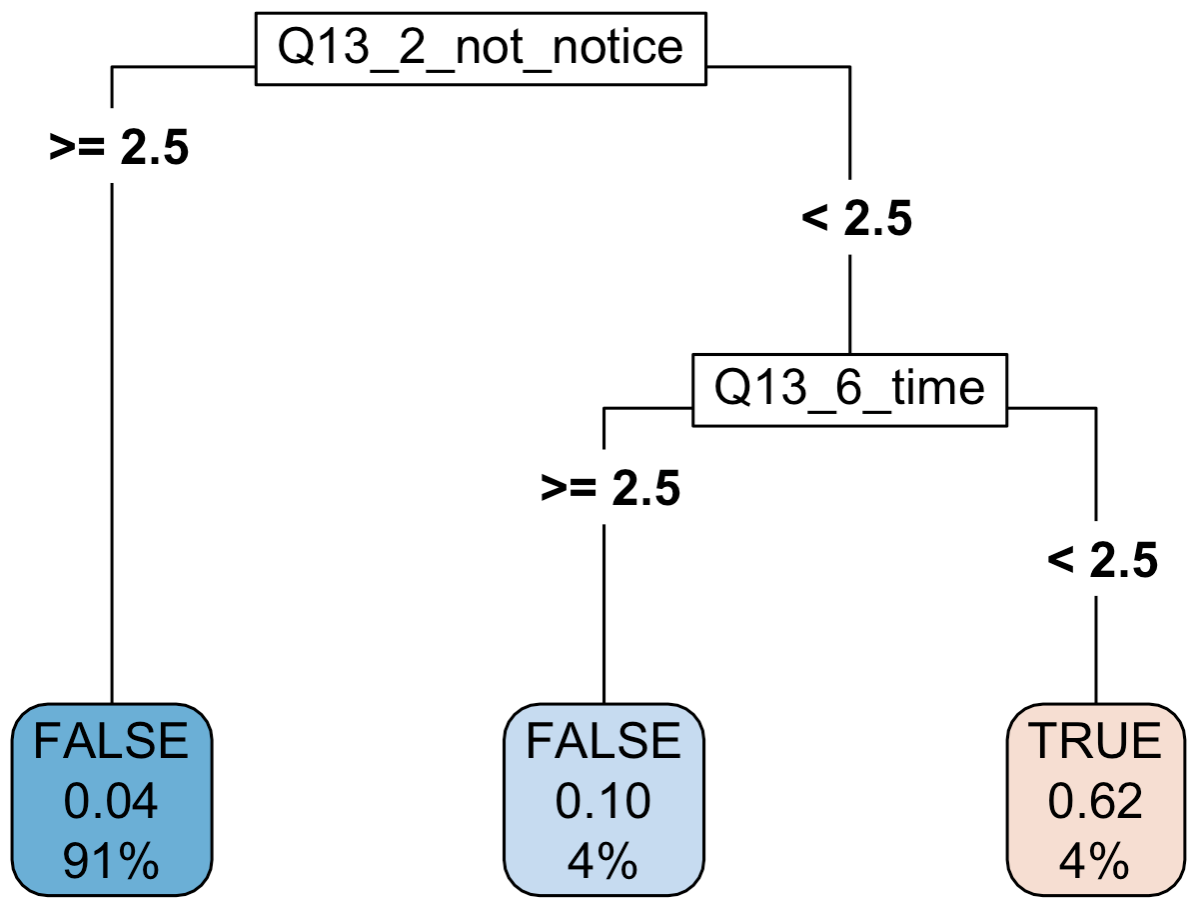
The classification tree resulting from running the rpart algorithm with the hyperparameters set according to Table 1 obtained via the cross validation procedure. It is worth noting that the rpart algorithm identifies the relations with parents as the most important variables for diagnosing mood disorder.

The *mmce* for this model was 0.03990. As depicted in the figure below, the resulting tree is very simple—it only uses two variables, both of which reflect the relationships between the child and the parent, i.e., question 13, subquestions 2 and 6. This question was the following: “Rate each of the statements below according to how well they fit the reality of your relation with your parents. Use the scale 1–5, where 1 means fully true and 5 means not true at all.” The two subquestions were: “Sometimes I feel my parents do not notice me” (2) and “My parents devote little time to me” (see the online resources). It should be stressed that the variables presented in Figure 1 also represent the surrogates, which for obvious reasons are not shown in the tree, so in fact it is not as simple—this is explored in more detail below. We believe this is a very powerful and highly interpretable result with a big potential for educational and therapeutic applications.

**Figure 2:**
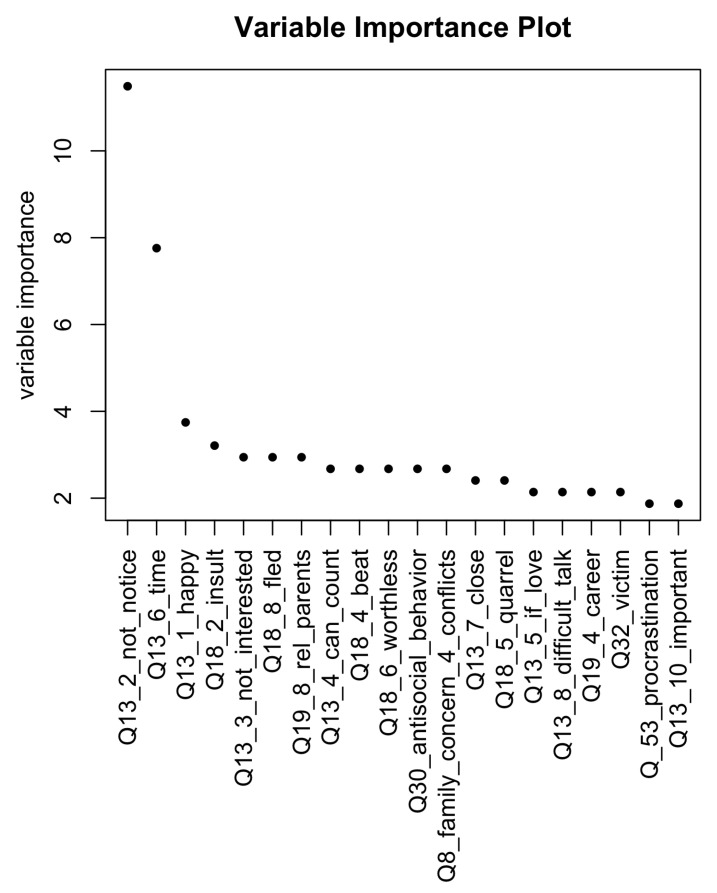
Variable importance according to the rpart algorithm. The plot presents the first 20 variables ordered according to their importance (see the main text). Thirteen of these variables are related to the child’s relations with parents.

The full ranking of variable importance as reported by the rpart algorithm is presented in Figure 2. Variable importance is the sum of the adjusted agreement of the split measures for each split for which it was the primary variable, plus the adjusted agreement for all splits in which it was a surrogate. This value is scaled to sum to 100 in the figure below [13].

It is worth noting that out of the first 20 most important variables, nine are subquestions of question 13 and four are subquestions of question 18, which was “During the last month, did (1-no, never/2-once /3-more than once) your parents did the following:” (a number of negative actions were listed as subquestions—for details see the supplementary materials). Both these questions concern the child’s relations with their parents. It is worth stressing again that in total, 13 out of 20 most important variables reflect the relationships with parents. As we noted before, this seems to be a significant result both from the cognitive point of view and also from the practical point of view, i.e., as far as interventions to correct mood disorders are concerned.

#### 3.1.2. Random Forest Model

The values of the hyperparameters tuned by the cross validation procedure are the following (see Table 2):

The *mmce* for this model was 0.06609.

#### 3.1.3. Xgboost Model

The values of the hyperparameters tuned by the cross-validation procedure are the following (see Table 3):

The *mmce* for this model was 0.0586.

### 3.2. Models Performance

In this section, we analyze the performance of the models tuned and trained in the previous section on the test data, which did not enter any analyses so far. In other words, these data are totally unknown to the models.

#### 3.2.1. Confusion Matrices

In this section, we compare the confusion matrices on the test data for the three models (see Table 4). The matrices are shown side by side for easier comparison.

#### 3.2.2. Error Rates

The misclassification errors for the models are as follows: for the rpart model it is 0.03074, for the random forest it is 0.0644, and for the XGBoost model it is 0.06295.

### 3.3. Drill down into the RPart Model and XGBoost Model

Since the rpart model is without any question the best model, it is worthwhile to drill down and see how well it identifies the most severe cases of mood disorder and the less severe cases (class 6 corresponds to the most severe disorders and class 5 to the less severe but still classified as severe disorders—see the Introduction). For comparison, we also drill down into the XGBoost model. We drop the the random forest model, since it does not identify any cases of mood disorder.

The rpart algorithm correctly identifies 6 out of 8 the most severe mood disorders, which, if this held for a larger group, would constitute 75% of the most severe cases. It also identifies 24 of 36 less severe mood disorders, which constitutes 66.67% of the less severe cases in the test set.

The XGBoost correctly identifies 1 out of 8 the most severe mood disorders, 12.5% of the most severe cases. It also identifies 4 of 36 less severe mood disorders, which constitute 11.11% of the less severe cases in the test set.

#### Expected Numbers in the Non-Classified Group

Out of the 221 cases in which the mood disorder could not be diagnosed, the rpart model predicts 21 cases of severe mood disorder (classes 6 or 5), the XGBoost model predicts 3 cases, and the random forest does not predict any cases.

We have no way of checking the correctness of these results—they are only given here as a possible application of the methodology presented in this paper.

## 4. Discussion

In the present paper we have used classification trees and related techniques to explore a mood disorder dataset and to build predictive models to identify students at risk for severe mood disorders. We have used classification-tree-related machine learning techniques, namely classification trees, random forests, and XGBoost. Since the dataset was heavy in missing values, in the theoretical exposition of the techniques, we concentrated on the approach to missing data in each of the three algorithms, rather than on the numerical optimization procedures.

From the results section, we can see that the most effective algorithm, as far as the misclassification error is concerned, was the rpart classification tree algorithm. The mmce for this model for training data was 0.0399 and for test data it was 0.03074. This can be contrasted with 0.06609 and 0.0644, respectively, for random forests and 0.0586 and 0.06295, respectively, for XGBoost. The predictive power of the rpart algorithm was also superior to the other two algorithms. This algorithm identified 30 out 44 cases of severe mood disorders in the test group, which is just around 68%. The classification tree also correctly identifies 75% of the most severe cases and 66.66% of the less severe (but still counted as severe) cases. The XGBoost identified only 4 out of 44 cases and the random forest identified none.

The observation about the random forest algorithm not identifying any cases of the severe mood disorder cases but still having an acceptable *mmce* is very interesting. It stresses how the reason the analyzed dataset is difficult to use with machine learning algorithms is that it is highly unbalanced. Indeed, there are very few cases of severe mood disorder contrasted with no or milder mood disorder. The imbalance is so high that a strategy based on classifying all cases as not having severe mood disorder is very effective and yields a low *mmce*—this is actually the strategy assumed by the random forest algorithm. This strategy maximizes the specificity of the model, which is 100% at the expense of sensitivity, which becomes equal to 0. In the present case, specificity is not important; we are only interested in sensitivity—we could accept an algorithm that misclassifies cases with no or mild mood disorder as having severe mood disorder if the model is sensitive and identifies most or all of true severe mood disorder cases. We can see that both the random forest and the XGBoost algorithms fail, with random forests failing particularly badly, even though this is not visible in the *mmce*.

The result that the classification tree outperforms random forests and XGBoost is an unusual one. The two latter algorithms were introduced as an improvement over the classification trees [9,10,16,17,18].

We believe that the reason for this result is the inadequate way of dealing with missing data for the two more modern algorithms. The rpart algorithm deals with absence of data, naturally, by using surrogate variables, the random forest and XGBoost algorithms using imputations, at least in the present paper (compare Section 1.5.1). We believe that the rpart algorithm did a better job avoiding absence of data by using surrogates than the other two algorithms, which assumed that data were missing completely at random (compare [9]) and imputed the data. As we remarked in the Introduction, we do not believe that data were missing completely at random. We (at least those of us more experienced in the social sciences) notice that some of the questions are leading, some are missing crucial options or are just too complex or using too involved a style for some adolescents to be able to process. In our opinion, assuming that answers are missing at random is assuming too much and in fact introducing information that is not there, thus modifying the training procedure.

The other aim of this paper was to explore the dataset and find out what the correlates of severe mood disorder are in this dataset. As we explained in the Introduction, this dataset does not render itself to standard statistical methods.

The only method out of those employed in the present paper that can provide interpretation is the classification tree [9,10]. It is a fortunate coincidence that this method turns out to be the most effective one as far as *mmce* and model sensitivity are concerned.

As demonstrated in the results section, the most important factor influencing severe mood disorder is the relationships with parents. The worse the relations with the parents are, the more often the case traversing the trained tree is sent to the “severe” bucket. It is interesting to notice that the analysis of variable importance yields the result that out of the 20 most important variables, 13 describes the relationships with parents (these correspond to questions 13 and 18—see the supplementary materials). Furthermore, the basic graphical representation of the classification tree built by the rpart algorithm only contains questions relating to parents (this graphical representation cannot, of course, depict the surrogates). This finding is strongly validated by the performance of the final model, which mostly uses the variables related to the parent relationship for prediction.

This result is partly corroborated by [3]; however, we only see one of the factors listed there, namely relations with parents, making it the strongest one—we hardly see any evidence for the other factors in our analysis. The importance of relations with parents is also corroborated by, e.g., [30,31]; however, our study is different as it tries to rank relationship with parents against other possible correlates, some of which are also related to the family but are not specifically aimed at the relationships with parents. Thus, the most important message of this paper as far as social and medical issues are concerned is that the relationship with parents is the most important factor in mood disorders in adolescents.

There are some important limitations of the present study. First of all, we want to stress that we do not postulate a general utility of the models created in this paper. To establish mood disorder, it is much simpler to just administer the Burns checklist. The predictive machine learning considerations from this paper should be viewed as being more methodological than prescriptive. The methodological conclusion following from our paper is the observation that the rpart algorithm deals with missing data much better than the other algorithm and the predictive power and sensitivity bear this observation out. A much more important conclusion is the interpretative one, in which we find that the covariates reflecting the adolescents’ relationships with their parents are the most important for developing mood disorders.

Another limitation of the study is the fact that we did not try to tune each model as much as possible. We are aware that there are ways to make the models more sensitive, but since one of the main goals was to compare the three models, we did this to give all of them an equal footing.

One of the possible ways to improve the performance of the random forest and xgboost models is using the approach of undersampling the majority class/oversampling the minority class [32]. Additionally, all analyzed models might benefit from using a different perfomance measure, such recall, F-measure, or AUC. This will be attempted in future—in this paper, our main concerns were the exploratory/explanatory aspects of the analysis, which allowed us to interpret the data, as well as comparing the sensitivity of the various models to missing data without tuning. It should, however, be clear that there is a lot of room for predictive power improvement as far as the random forest and xgboost sampling strategies are concerned, as well as the performance measures used in all three models.

## 5. Conclusions

Studies applying machine learning to social sciences and data derived from this domain are quite rare, with References [32,33,34] being three examples. We believe that the conclusions about missing data related to the machine learning methodology as well as the finding about parent relationships will be of interest to a broad readership oriented both towards technology and social sciences.

## Figures and Tables

**Table 1:** Results of the cross validation hyperparameter tuning procedure.

minsplit	minbucket	cp	maxdepth	maxsurrogate
18	16	0.0605	5	94

**Table 2:** Results of the cross validation hyperparameter tuning procedure for the random forest model.

ntree	mtry	nodesize	maxnodes
500	10	1	16

**Table 3:** Results of the cross validation hyperparameter tuning procedure for the random forest model.

eta	gamma	max_depth	min_child_weight	subsample	colsample_bytree	nrounds
0.211	1.44	4	2.98	0.662	0.614	20

**Table 4:** Comparison of confusion matrices across the three models. In all matrices, F stands for FALSE, i.e., no mood disorder, T stands for TRUE, i.e., severe mood disorder (classes 5 and 6) as diagnosed by the Burns test, and err stands for error. Column titles refer to the actual count, while row titles to predicted count.

rpart			
	F	T	err
F	632	7	7
T	14	30	14
err	14	7	21
random forest			
	F	T	err
F	639	0	0
T	44	0	44
err	44	0	44
XGBoost			
	F	T	err
F	635	4	4
T	40	4	39
err	40	4	42

## Data Availability

Data available on request due to legal restrictions. The data presented in this study are available on request from the corresponding author. The data are not publicly available due to Polish law and the agreements with the institution granting them.

## Supplementary Materials

The following are available online at https://www.mdpi.com/article/10.3390/e23091210/s1.
