# Supplementary material for: Mood Disorder Detection in Adolescents by Classification Trees, Random Forests and XGBoost in Presence of Missing Data
Source: Entropy (Basel). 2021 Sep 14;23(9):1210. doi: 10.3390/e23091210 (PMC8468933; doi:10.3390/e23091210)
Supplement: Supplementary file 1 [file entropy-23-01210-s001.zip › social_AI/trees.nb.html]

R Notebook


Code 

- Show All Code
- Hide All Code
- Download Rmd

# R Notebook


```
library(mlr)
```


```
Ładowanie wymaganego pakietu: ParamHelpers
Warning message: 'mlr' is in 'maintenance-only' mode since July 2019. Future development will only happen
in 'mlr3' (<https://mlr3.mlr-org.com>). Due to the focus on 'mlr3' there might be uncaught bugs meanwhile
in {mlr} - please consider switching.
```


```
library(magrittr)
library(tidyverse)
```


```
Registered S3 methods overwritten by 'dbplyr':
  method         from
  print.tbl_lazy     
  print.tbl_sql      
── Attaching packages ──────────────────────────────────────────────────────────────────────────────── tidyverse 1.3.1 ──
✓ ggplot2 3.3.3     ✓ purrr   0.3.4
✓ tibble  3.1.1     ✓ dplyr   1.0.5
✓ tidyr   1.1.3     ✓ stringr 1.4.0
✓ readr   1.4.0     ✓ forcats 0.5.1
── Conflicts ─────────────────────────────────────────────────────────────────────────────────── tidyverse_conflicts() ──
x tidyr::extract()   masks magrittr::extract()
x dplyr::filter()    masks stats::filter()
x dplyr::lag()       masks stats::lag()
x purrr::set_names() masks magrittr::set_names()
```


# Data preparation


```
#' Helper function drawing rows from a subset of the dataset
#' @param dataset dataset
#' @param test_fraction what fraction of each class should become a test set
draw_train_test <- function(dataset, test_fraction) {
  N_row <- nrow(dataset)
  set.seed(777)
  test_rows <- sample(1:N_row, as.integer(test_fraction * N_row), replace = FALSE)
  train_rows <- (1:N_row)[!(1:N_row %in% test_rows)]
  list(train = dataset[train_rows, ], test = dataset[test_rows, ])
}
#' Function distributing the data to training and test sets
#' @param dataset dataset
#' @param taget target column
#' @param test_fraction what fraction of each class should become a test set
make_train_test <- function(dataset, target, test_fraction) {
  result_train <- NULL
  result_test <- NULL
  for (target_elem in unique(dataset[[target]])) {
    train_test <- draw_train_test(dataset[dataset[[target]] == target_elem, ], test_fraction)
    result_train <- bind_rows(result_train, train_test[["train"]])
    result_test <- bind_rows(result_test, train_test[["test"]])
  }
  list(train = result_train, test = result_test)
}
#' Function plotting variable importance
#' @param rpart_model trained rpart model
#' @param var_no number of variables
var_imp_plot <- function(rpart_model, var_no = 10) {
  png("var_importance.png", width = 1600, height = 1900, res= 300)
  oldpar <- par(mar = c(13, 4, 3, 2))
  plot(rpart_model$learner.model$variable.importance[1:var_no], xlab = "", 
       ylab="", xaxt = "n", pch = 20,
       main = "Variable Importance Plot")
  title(ylab = "variable importance", mgp = c(2.3, 1, 0))
  axis(1, at=1:var_no, labels = names(rpart_model$learner.model$variable.importance)[1:var_no], las=2)
  par(oldpar)
  dev.off()
}
```


```
mood_data <- readRDS('mood_data.RDS')

# split dataset into those with and without mood data
mood_data_all <- mood_data[!is.na(mood_data$mood), ]
mood_data_na <- mood_data[is.na(mood_data$mood), ]

mood_train_test <- make_train_test(mood_data_all, target = "mood", test_fraction = 0.3)
mood_train <- mood_train_test[["train"]]
mood_test <- mood_train_test[["test"]]
saveRDS(mood_test, file = "mood_test_full.RDS")
saveRDS(mood_train, file = "mood_train_full.RDS")

## this is specific to a classification task
severity_train <- mood_train$mood %in% c(5, 6)
severity_test <- mood_test$mood %in% c(5, 6)

mood_data_train_class <- mood_train %>% 
  select(-c('mood')) %>% 
  add_column(severity = severity_train, .before = 1)

mood_data_test_class <- mood_test %>% 
  select(-c('mood')) %>% 
  add_column(severity = severity_test, .before = 1)

saveRDS(mood_data_train_class, "mood_train.RDS")
saveRDS(mood_data_test_class, "mood_test.RDS")
saveRDS(mood_data_na, "mood_data_na.RDS")
```

# The rpart tree algorithm

## Task, Learner and Training


```
# TUTAJ ZMIENIAJ!
severity_task <- makeClassifTask(data = mood_data_train_class, target = "severity")
```


```
Provided data is not a pure data.frame but from class tbl_df, hence it will be converted.
```


```
tree_learner <- makeLearner("classif.rpart")
getParamSet(tree_learner)
```

## Hyperparameter tuning


```
tree_parameter_space <- makeParamSet(
  makeIntegerParam("minsplit", lower = 1, upper = 20),
  makeIntegerParam("minbucket", lower = 1, upper = 16),
  makeNumericParam("cp", lower = 0.01, upper = 0.1),
  makeIntegerParam("maxdepth", lower = 3, upper = 10),
  makeIntegerParam("maxsurrogate", lower = 90, upper = 110)
)

rand_search <- makeTuneControlRandom(maxit = 200)
cv_for_tuning <- makeResampleDesc("CV", iters = 5, stratify = TRUE)

library(parallel)
library(parallelMap)
print(paste("Detected ", detectCores(), " cores", collapse = ""))
```


```
[1] "Detected  4  cores"
```


```
parallelStartSocket(cpus = detectCores())
```


```
Starting parallelization in mode=socket with cpus=4.
```


```
tuned_tree_pars <- tuneParams(tree_learner, task = severity_task,
                            resampling = cv_for_tuning,
                            par.set = tree_parameter_space, 
                            control = rand_search)
```


```
[Tune] Started tuning learner classif.rpart for parameter set:
```


```
With control class: TuneControlRandom
Imputation value: 1
Exporting objects to slaves for mode socket: .mlr.slave.options
Mapping in parallel: mode = socket; level = mlr.tuneParams; cpus = 4; elements = 200.
[Tune] Result: minsplit=20; minbucket=16; cp=0.0157; maxdepth=3; maxsurrogate=101 : mmce.test.mean=0.041190
```


```
parallelStop()
```


```
Stopped parallelization. All cleaned up.
```


```
tuned_tree_pars
```


```
Tune result:
Op. pars: minsplit=20; minbucket=16; cp=0.0157; maxdepth=3; maxsurrogate=101
mmce.test.mean=0.041190
```

## Training the model


```
tuned_tree <- setHyperPars(tree_learner, par.vals = tuned_tree_pars$x)
tuned_tree_model <- train(tuned_tree, severity_task)
saveRDS(tuned_tree_model, file = "tuned_tree_model.RDS")
```


```
library(rpart.plot)
```


```
Ładowanie wymaganego pakietu: rpart
```


```
tree_model_data <- getLearnerModel(tuned_tree_model)
png(filename = "drzewko.png", res = 300, width = 1800, height = 1800)
rpart.plot(tree_model_data, roundint = FALSE, 
           box.palette = "BuBn",
           type = 5,
           fallen.leaves = TRUE)
dev.off()
```


```
null device 
          1
```


```
severity_task_data <- severity_task$env$data
predicted_mood_tree <- predict(tuned_tree_model, newdata = severity_task_data)
performance <- performance(predicted_mood_tree, measures = list(mmce, acc))
calculateConfusionMatrix(predicted_mood_tree)
```


```
        predicted
true     FALSE TRUE -err.-
  FALSE   1490    7      7
  TRUE      89   18     89
  -err.-    89    7     96
```

## Large tree


```
large_tree_pars <- list(
  maxdepth = 10, 
  minsplit = tuned_tree_pars$x$minsplit, 
  maxsurrogate =  tuned_tree_pars$x$maxsurrogate,  
  minbucket = 1, 
  cp = 0.00
)

large_tree <- setHyperPars(tree_learner, par.vals = large_tree_pars)
large_tree_model <- train(large_tree, severity_task)
saveRDS(large_tree_model, file = "large_tree_model.RDS")
tree_large_model_data <- getLearnerModel(large_tree_model)
png(filename = "drzewko_big.png", res = 300, width = 1800, height = 1800)
rpart.plot(tree_large_model_data, roundint = FALSE, 
           box.palette = "BuBn",
           type = 5,
           fallen.leaves = TRUE)
dev.off()
```


```
null device 
          1
```


```
predicted_mood_tree <- predict(large_tree_model, newdata = severity_task_data)
performance <- performance(predicted_mood_tree, measures = list(mmce, acc))
print(performance)
```


```
      mmce        acc 
0.03990025 0.96009975
```


```
calculateConfusionMatrix(predicted_mood_tree)
```


```
        predicted
true     FALSE TRUE -err.-
  FALSE   1478   19     19
  TRUE      45   62     45
  -err.-    45   19     64
```

## Exploring the model


```
printcp(tree_model_data, digits = 3)
```


```
Classification tree:
rpart::rpart(formula = f, data = d, xval = 0, minsplit = 20, 
    minbucket = 16, cp = 0.0157401523250155, maxdepth = 3, maxsurrogate = 101)

Variables actually used in tree construction:
[1] Q18_6_worthless Q27_comparisons

Root node error: 107/1604 = 0.0667

n= 1604 

      CP nsplit rel error
1 0.0514      0     1.000
2 0.0157      2     0.897
```


```
summary(tree_model_data)
```


```
Call:
rpart::rpart(formula = f, data = d, xval = 0, minsplit = 20, 
    minbucket = 16, cp = 0.0157401523250155, maxdepth = 3, maxsurrogate = 101)
  n= 1604 

          CP nsplit rel error
1 0.05140187      0 1.0000000
2 0.01574015      2 0.8971963

Variable importance
         Q18_6_worthless          Q27_comparisons           Q18_7_felt_bad      Q20_self_assessment 
                      53                       20                        3                        2 
          Q28_peer_group               Q18_8_fled        Q_52_wasting_time              Q13_1_happy 
                       2                        2                        2                        2 
          Q15_13_nothing             Q19_2_health             Q19_6_rel_bg               Q19_9_time 
                       2                        2                        2                        2 
     Q2_school_plan_real               Q32_victim     Q_53_procrastination          Q13_4_can_count 
                       2                        2                        1                        1 
Q26_school_relationships       Q3_edu_fear_4_work  Q30_antisocial_behavior              Q34_support 
                       1                        1                        1                        1 
 Q6_father_does_not_work 
                       1 

Node number 1: 1604 observations,    complexity param=0.05140187
  predicted class=FALSE  expected loss=0.06670823  P(node) =1
    class counts:  1497   107
   probabilities: 0.933 0.067 
  left son=2 (1525 obs) right son=3 (79 obs)
  Primary splits:
      Q18_6_worthless  < 2.5      to the left,  improve=19.36646, (14 missing)
      Q13_6_time       < 2.5      to the right, improve=16.83481, (15 missing)
      Q18_5_quarrel    < 1.5      to the left,  improve=13.99542, (11 missing)
      Q13_9_important  < 2.5      to the left,  improve=12.89911, (9 missing)
      Q13_2_not_notice < 3.5      to the right, improve=12.76664, (7 missing)
  Surrogate splits:
      Q18_7_felt_bad < 2.5      to the left,  agree=0.953, adj=0.051, (8 split)
      Q18_8_fled     < 2.5      to the left,  agree=0.953, adj=0.038, (0 split)

Node number 2: 1525 observations
  predicted class=FALSE  expected loss=0.04918033  P(node) =0.9507481
    class counts:  1450    75
   probabilities: 0.951 0.049 

Node number 3: 79 observations,    complexity param=0.05140187
  predicted class=FALSE  expected loss=0.4050633  P(node) =0.04925187
    class counts:    47    32
   probabilities: 0.595 0.405 
  left son=6 (54 obs) right son=7 (25 obs)
  Primary splits:
      Q27_comparisons < 1.771242 to the right, improve=7.255209, (0 missing)
      Q51_1_phone     < 2.75     to the left,  improve=5.206590, (2 missing)
      Q13_1_happy     < 3.5      to the left,  improve=4.366234, (2 missing)
      Q15_8_meet      < 0.5      to the right, improve=4.310256, (1 missing)
      Q51_2_sms       < 3.75     to the left,  improve=3.933770, (8 missing)
  Surrogate splits:
      Q20_self_assessment      < 2.166667 to the right, agree=0.722, adj=0.12, (0 split)
      Q28_peer_group           splits as  LR,           agree=0.722, adj=0.12, (0 split)
      Q2_school_plan_real      < 2.5      to the right, agree=0.709, adj=0.08, (0 split)
      Q13_1_happy              < 3.5      to the left,  agree=0.709, adj=0.08, (0 split)
      Q15_13_nothing           < 0.5      to the left,  agree=0.709, adj=0.08, (0 split)
      Q19_2_health             < 1.5      to the right, agree=0.709, adj=0.08, (0 split)
      Q19_6_rel_bg             < 3.5      to the right, agree=0.709, adj=0.08, (0 split)
      Q19_9_time               < 3.5      to the right, agree=0.709, adj=0.08, (0 split)
      Q32_victim               < 1.083333 to the left,  agree=0.709, adj=0.08, (0 split)
      Q_52_wasting_time        < 2.75     to the left,  agree=0.709, adj=0.08, (0 split)
      Q3_edu_fear_4_work       splits as  RL,           agree=0.696, adj=0.04, (0 split)
      Q6_father_does_not_work  < 0.5      to the left,  agree=0.696, adj=0.04, (0 split)
      Q13_4_can_count          < 4.5      to the left,  agree=0.696, adj=0.04, (0 split)
      Q26_school_relationships < 1.5      to the right, agree=0.696, adj=0.04, (0 split)
      Q30_antisocial_behavior  < 0.025    to the right, agree=0.696, adj=0.04, (0 split)
      Q34_support              < 0.5      to the right, agree=0.696, adj=0.04, (0 split)
      Q_53_procrastination     < 1.083333 to the right, agree=0.696, adj=0.04, (0 split)

Node number 6: 54 observations
  predicted class=FALSE  expected loss=0.2592593  P(node) =0.03366584
    class counts:    40    14
   probabilities: 0.741 0.259 

Node number 7: 25 observations
  predicted class=TRUE   expected loss=0.28  P(node) =0.01558603
    class counts:     7    18
   probabilities: 0.280 0.720
```


```
var_imp_plot(large_tree_model, 20)
```


```
null device 
          1
```

## Cross-validation


```
outer <- makeResampleDesc("CV", iters = 5)
tree_wrapper <- makeTuneWrapper("classif.rpart", resampling = cv_for_tuning,
                               par.set = tree_parameter_space,
                               control = rand_search)
parallelStartSocket(cpus = detectCores())
```


```
Starting parallelization in mode=socket with cpus=4.
```


```
cv_with_tuning <- resample(tree_wrapper, severity_task, resampling = outer)
```


```
Exporting objects to slaves for mode socket: .mlr.slave.options
Resampling: cross-validation
Measures:             mmce      
Mapping in parallel: mode = socket; level = mlr.resample; cpus = 4; elements = 5.


Aggregated Result: mmce.test.mean=0.03990
```


```
parallelStop()
```


```
Stopped parallelization. All cleaned up.
```


```
cv_with_tuning
```


```
Resample Result
Task: mood_data_train_class
Learner: classif.rpart.tuned
Aggr perf: mmce.test.mean=0.03990
Runtime: 769.024
```

LS0tCnRpdGxlOiAiUiBOb3RlYm9vayIKb3V0cHV0OiBodG1sX25vdGVib29rCi0tLQoKCmBgYHtyIHNldHVwLCBpbmNsdWRlPUZBTFNFfQprbml0cjo6b3B0c19jaHVuayRzZXQoZWNobyA9IFRSVUUpCmBgYApgYGB7cn0KbGlicmFyeShtbHIpCmxpYnJhcnkobWFncml0dHIpCmxpYnJhcnkodGlkeXZlcnNlKQpgYGAKIyBEYXRhIHByZXBhcmF0aW9uCmBgYHtyfQojJyBIZWxwZXIgZnVuY3Rpb24gZHJhd2luZyByb3dzIGZyb20gYSBzdWJzZXQgb2YgdGhlIGRhdGFzZXQKIycgQHBhcmFtIGRhdGFzZXQgZGF0YXNldAojJyBAcGFyYW0gdGVzdF9mcmFjdGlvbiB3aGF0IGZyYWN0aW9uIG9mIGVhY2ggY2xhc3Mgc2hvdWxkIGJlY29tZSBhIHRlc3Qgc2V0CmRyYXdfdHJhaW5fdGVzdCA8LSBmdW5jdGlvbihkYXRhc2V0LCB0ZXN0X2ZyYWN0aW9uKSB7CiAgTl9yb3cgPC0gbnJvdyhkYXRhc2V0KQogIHNldC5zZWVkKDc3NykKICB0ZXN0X3Jvd3MgPC0gc2FtcGxlKDE6Tl9yb3csIGFzLmludGVnZXIodGVzdF9mcmFjdGlvbiAqIE5fcm93KSwgcmVwbGFjZSA9IEZBTFNFKQogIHRyYWluX3Jvd3MgPC0gKDE6Tl9yb3cpWyEoMTpOX3JvdyAlaW4lIHRlc3Rfcm93cyldCiAgbGlzdCh0cmFpbiA9IGRhdGFzZXRbdHJhaW5fcm93cywgXSwgdGVzdCA9IGRhdGFzZXRbdGVzdF9yb3dzLCBdKQp9CiMnIEZ1bmN0aW9uIGRpc3RyaWJ1dGluZyB0aGUgZGF0YSB0byB0cmFpbmluZyBhbmQgdGVzdCBzZXRzCiMnIEBwYXJhbSBkYXRhc2V0IGRhdGFzZXQKIycgQHBhcmFtIHRhZ2V0IHRhcmdldCBjb2x1bW4KIycgQHBhcmFtIHRlc3RfZnJhY3Rpb24gd2hhdCBmcmFjdGlvbiBvZiBlYWNoIGNsYXNzIHNob3VsZCBiZWNvbWUgYSB0ZXN0IHNldAptYWtlX3RyYWluX3Rlc3QgPC0gZnVuY3Rpb24oZGF0YXNldCwgdGFyZ2V0LCB0ZXN0X2ZyYWN0aW9uKSB7CiAgcmVzdWx0X3RyYWluIDwtIE5VTEwKICByZXN1bHRfdGVzdCA8LSBOVUxMCiAgZm9yICh0YXJnZXRfZWxlbSBpbiB1bmlxdWUoZGF0YXNldFtbdGFyZ2V0XV0pKSB7CiAgICB0cmFpbl90ZXN0IDwtIGRyYXdfdHJhaW5fdGVzdChkYXRhc2V0W2RhdGFzZXRbW3RhcmdldF1dID09IHRhcmdldF9lbGVtLCBdLCB0ZXN0X2ZyYWN0aW9uKQogICAgcmVzdWx0X3RyYWluIDwtIGJpbmRfcm93cyhyZXN1bHRfdHJhaW4sIHRyYWluX3Rlc3RbWyJ0cmFpbiJdXSkKICAgIHJlc3VsdF90ZXN0IDwtIGJpbmRfcm93cyhyZXN1bHRfdGVzdCwgdHJhaW5fdGVzdFtbInRlc3QiXV0pCiAgfQogIGxpc3QodHJhaW4gPSByZXN1bHRfdHJhaW4sIHRlc3QgPSByZXN1bHRfdGVzdCkKfQojJyBGdW5jdGlvbiBwbG90dGluZyB2YXJpYWJsZSBpbXBvcnRhbmNlCiMnIEBwYXJhbSBycGFydF9tb2RlbCB0cmFpbmVkIHJwYXJ0IG1vZGVsCiMnIEBwYXJhbSB2YXJfbm8gbnVtYmVyIG9mIHZhcmlhYmxlcwp2YXJfaW1wX3Bsb3QgPC0gZnVuY3Rpb24ocnBhcnRfbW9kZWwsIHZhcl9ubyA9IDEwKSB7CiAgcG5nKCJ2YXJfaW1wb3J0YW5jZS5wbmciLCB3aWR0aCA9IDE2MDAsIGhlaWdodCA9IDE5MDAsIHJlcz0gMzAwKQogIG9sZHBhciA8LSBwYXIobWFyID0gYygxMywgNCwgMywgMikpCiAgcGxvdChycGFydF9tb2RlbCRsZWFybmVyLm1vZGVsJHZhcmlhYmxlLmltcG9ydGFuY2VbMTp2YXJfbm9dLCB4bGFiID0gIiIsIAogICAgICAgeWxhYj0iIiwgeGF4dCA9ICJuIiwgcGNoID0gMjAsCiAgICAgICBtYWluID0gIlZhcmlhYmxlIEltcG9ydGFuY2UgUGxvdCIpCiAgdGl0bGUoeWxhYiA9ICJ2YXJpYWJsZSBpbXBvcnRhbmNlIiwgbWdwID0gYygyLjMsIDEsIDApKQogIGF4aXMoMSwgYXQ9MTp2YXJfbm8sIGxhYmVscyA9IG5hbWVzKHJwYXJ0X21vZGVsJGxlYXJuZXIubW9kZWwkdmFyaWFibGUuaW1wb3J0YW5jZSlbMTp2YXJfbm9dLCBsYXM9MikKICBwYXIob2xkcGFyKQogIGRldi5vZmYoKQp9CmBgYAoKCmBgYHtyfQptb29kX2RhdGEgPC0gcmVhZFJEUygnbW9vZF9kYXRhLlJEUycpCgojIHNwbGl0IGRhdGFzZXQgaW50byB0aG9zZSB3aXRoIGFuZCB3aXRob3V0IG1vb2QgZGF0YQptb29kX2RhdGFfYWxsIDwtIG1vb2RfZGF0YVshaXMubmEobW9vZF9kYXRhJG1vb2QpLCBdCm1vb2RfZGF0YV9uYSA8LSBtb29kX2RhdGFbaXMubmEobW9vZF9kYXRhJG1vb2QpLCBdCgptb29kX3RyYWluX3Rlc3QgPC0gbWFrZV90cmFpbl90ZXN0KG1vb2RfZGF0YV9hbGwsIHRhcmdldCA9ICJtb29kIiwgdGVzdF9mcmFjdGlvbiA9IDAuMykKbW9vZF90cmFpbiA8LSBtb29kX3RyYWluX3Rlc3RbWyJ0cmFpbiJdXQptb29kX3Rlc3QgPC0gbW9vZF90cmFpbl90ZXN0W1sidGVzdCJdXQpzYXZlUkRTKG1vb2RfdGVzdCwgZmlsZSA9ICJtb29kX3Rlc3RfZnVsbC5SRFMiKQpzYXZlUkRTKG1vb2RfdHJhaW4sIGZpbGUgPSAibW9vZF90cmFpbl9mdWxsLlJEUyIpCgojIyB0aGlzIGlzIHNwZWNpZmljIHRvIGEgY2xhc3NpZmljYXRpb24gdGFzawpzZXZlcml0eV90cmFpbiA8LSBtb29kX3RyYWluJG1vb2QgJWluJSBjKDUsIDYpCnNldmVyaXR5X3Rlc3QgPC0gbW9vZF90ZXN0JG1vb2QgJWluJSBjKDUsIDYpCgptb29kX2RhdGFfdHJhaW5fY2xhc3MgPC0gbW9vZF90cmFpbiAlPiUgCiAgc2VsZWN0KC1jKCdtb29kJykpICU+JSAKICBhZGRfY29sdW1uKHNldmVyaXR5ID0gc2V2ZXJpdHlfdHJhaW4sIC5iZWZvcmUgPSAxKQoKbW9vZF9kYXRhX3Rlc3RfY2xhc3MgPC0gbW9vZF90ZXN0ICU+JSAKICBzZWxlY3QoLWMoJ21vb2QnKSkgJT4lIAogIGFkZF9jb2x1bW4oc2V2ZXJpdHkgPSBzZXZlcml0eV90ZXN0LCAuYmVmb3JlID0gMSkKCnNhdmVSRFMobW9vZF9kYXRhX3RyYWluX2NsYXNzLCAibW9vZF90cmFpbi5SRFMiKQpzYXZlUkRTKG1vb2RfZGF0YV90ZXN0X2NsYXNzLCAibW9vZF90ZXN0LlJEUyIpCnNhdmVSRFMobW9vZF9kYXRhX25hLCAibW9vZF9kYXRhX25hLlJEUyIpCmBgYAoKIyBUaGUgcnBhcnQgdHJlZSBhbGdvcml0aG0KCiMjIFRhc2ssIExlYXJuZXIgYW5kIFRyYWluaW5nCmBgYHtyfQojIFRVVEFKIFpNSUVOSUFKIQpzZXZlcml0eV90YXNrIDwtIG1ha2VDbGFzc2lmVGFzayhkYXRhID0gbW9vZF9kYXRhX3RyYWluX2NsYXNzLCB0YXJnZXQgPSAic2V2ZXJpdHkiKQp0cmVlX2xlYXJuZXIgPC0gbWFrZUxlYXJuZXIoImNsYXNzaWYucnBhcnQiKQpnZXRQYXJhbVNldCh0cmVlX2xlYXJuZXIpCmBgYAoKIyMgSHlwZXJwYXJhbWV0ZXIgdHVuaW5nCgpgYGB7cn0KdHJlZV9wYXJhbWV0ZXJfc3BhY2UgPC0gbWFrZVBhcmFtU2V0KAogIG1ha2VJbnRlZ2VyUGFyYW0oIm1pbnNwbGl0IiwgbG93ZXIgPSAxLCB1cHBlciA9IDIwKSwKICBtYWtlSW50ZWdlclBhcmFtKCJtaW5idWNrZXQiLCBsb3dlciA9IDEsIHVwcGVyID0gMTYpLAogIG1ha2VOdW1lcmljUGFyYW0oImNwIiwgbG93ZXIgPSAwLjAxLCB1cHBlciA9IDAuMSksCiAgbWFrZUludGVnZXJQYXJhbSgibWF4ZGVwdGgiLCBsb3dlciA9IDMsIHVwcGVyID0gMTApLAogIG1ha2VJbnRlZ2VyUGFyYW0oIm1heHN1cnJvZ2F0ZSIsIGxvd2VyID0gOTAsIHVwcGVyID0gMTEwKQopCgpyYW5kX3NlYXJjaCA8LSBtYWtlVHVuZUNvbnRyb2xSYW5kb20obWF4aXQgPSAyMDApCmN2X2Zvcl90dW5pbmcgPC0gbWFrZVJlc2FtcGxlRGVzYygiQ1YiLCBpdGVycyA9IDUsIHN0cmF0aWZ5ID0gVFJVRSkKCmxpYnJhcnkocGFyYWxsZWwpCmxpYnJhcnkocGFyYWxsZWxNYXApCnByaW50KHBhc3RlKCJEZXRlY3RlZCAiLCBkZXRlY3RDb3JlcygpLCAiIGNvcmVzIiwgY29sbGFwc2UgPSAiIikpCnBhcmFsbGVsU3RhcnRTb2NrZXQoY3B1cyA9IGRldGVjdENvcmVzKCkpCnR1bmVkX3RyZWVfcGFycyA8LSB0dW5lUGFyYW1zKHRyZWVfbGVhcm5lciwgdGFzayA9IHNldmVyaXR5X3Rhc2ssCiAgICAgICAgICAgICAgICAgICAgICAgICAgICByZXNhbXBsaW5nID0gY3ZfZm9yX3R1bmluZywKICAgICAgICAgICAgICAgICAgICAgICAgICAgIHBhci5zZXQgPSB0cmVlX3BhcmFtZXRlcl9zcGFjZSwgCiAgICAgICAgICAgICAgICAgICAgICAgICAgICBjb250cm9sID0gcmFuZF9zZWFyY2gpCnBhcmFsbGVsU3RvcCgpCnR1bmVkX3RyZWVfcGFycwpgYGAKIyMgVHJhaW5pbmcgdGhlIG1vZGVsCgpgYGB7cn0KdHVuZWRfdHJlZSA8LSBzZXRIeXBlclBhcnModHJlZV9sZWFybmVyLCBwYXIudmFscyA9IHR1bmVkX3RyZWVfcGFycyR4KQp0dW5lZF90cmVlX21vZGVsIDwtIHRyYWluKHR1bmVkX3RyZWUsIHNldmVyaXR5X3Rhc2spCnNhdmVSRFModHVuZWRfdHJlZV9tb2RlbCwgZmlsZSA9ICJ0dW5lZF90cmVlX21vZGVsLlJEUyIpCmBgYAoKYGBge3J9CmxpYnJhcnkocnBhcnQucGxvdCkKdHJlZV9tb2RlbF9kYXRhIDwtIGdldExlYXJuZXJNb2RlbCh0dW5lZF90cmVlX21vZGVsKQpwbmcoZmlsZW5hbWUgPSAiZHJ6ZXdrby5wbmciLCByZXMgPSAzMDAsIHdpZHRoID0gMTgwMCwgaGVpZ2h0ID0gMTgwMCkKcnBhcnQucGxvdCh0cmVlX21vZGVsX2RhdGEsIHJvdW5kaW50ID0gRkFMU0UsIAogICAgICAgICAgIGJveC5wYWxldHRlID0gIkJ1Qm4iLAogICAgICAgICAgIHR5cGUgPSA1LAogICAgICAgICAgIGZhbGxlbi5sZWF2ZXMgPSBUUlVFKQpkZXYub2ZmKCkKc2V2ZXJpdHlfdGFza19kYXRhIDwtIHNldmVyaXR5X3Rhc2skZW52JGRhdGEKcHJlZGljdGVkX21vb2RfdHJlZSA8LSBwcmVkaWN0KHR1bmVkX3RyZWVfbW9kZWwsIG5ld2RhdGEgPSBzZXZlcml0eV90YXNrX2RhdGEpCnBlcmZvcm1hbmNlIDwtIHBlcmZvcm1hbmNlKHByZWRpY3RlZF9tb29kX3RyZWUsIG1lYXN1cmVzID0gbGlzdChtbWNlLCBhY2MpKQpjYWxjdWxhdGVDb25mdXNpb25NYXRyaXgocHJlZGljdGVkX21vb2RfdHJlZSkKYGBgCiMjIExhcmdlIHRyZWUKYGBge3J9CmxhcmdlX3RyZWVfcGFycyA8LSBsaXN0KAogIG1heGRlcHRoID0gMTAsIAogIG1pbnNwbGl0ID0gdHVuZWRfdHJlZV9wYXJzJHgkbWluc3BsaXQsIAogIG1heHN1cnJvZ2F0ZSA9ICB0dW5lZF90cmVlX3BhcnMkeCRtYXhzdXJyb2dhdGUsICAKICBtaW5idWNrZXQgPSAxLCAKICBjcCA9IDAuMDAKKQoKbGFyZ2VfdHJlZSA8LSBzZXRIeXBlclBhcnModHJlZV9sZWFybmVyLCBwYXIudmFscyA9IGxhcmdlX3RyZWVfcGFycykKbGFyZ2VfdHJlZV9tb2RlbCA8LSB0cmFpbihsYXJnZV90cmVlLCBzZXZlcml0eV90YXNrKQpzYXZlUkRTKGxhcmdlX3RyZWVfbW9kZWwsIGZpbGUgPSAibGFyZ2VfdHJlZV9tb2RlbC5SRFMiKQp0cmVlX2xhcmdlX21vZGVsX2RhdGEgPC0gZ2V0TGVhcm5lck1vZGVsKGxhcmdlX3RyZWVfbW9kZWwpCnBuZyhmaWxlbmFtZSA9ICJkcnpld2tvX2JpZy5wbmciLCByZXMgPSAzMDAsIHdpZHRoID0gMTgwMCwgaGVpZ2h0ID0gMTgwMCkKcnBhcnQucGxvdCh0cmVlX2xhcmdlX21vZGVsX2RhdGEsIHJvdW5kaW50ID0gRkFMU0UsIAogICAgICAgICAgIGJveC5wYWxldHRlID0gIkJ1Qm4iLAogICAgICAgICAgIHR5cGUgPSA1LAogICAgICAgICAgIGZhbGxlbi5sZWF2ZXMgPSBUUlVFKQpkZXYub2ZmKCkKCnByZWRpY3RlZF9tb29kX3RyZWUgPC0gcHJlZGljdChsYXJnZV90cmVlX21vZGVsLCBuZXdkYXRhID0gc2V2ZXJpdHlfdGFza19kYXRhKQpwZXJmb3JtYW5jZSA8LSBwZXJmb3JtYW5jZShwcmVkaWN0ZWRfbW9vZF90cmVlLCBtZWFzdXJlcyA9IGxpc3QobW1jZSwgYWNjKSkKcHJpbnQocGVyZm9ybWFuY2UpCmNhbGN1bGF0ZUNvbmZ1c2lvbk1hdHJpeChwcmVkaWN0ZWRfbW9vZF90cmVlKQpgYGAKCiMjIEV4cGxvcmluZyB0aGUgbW9kZWwKYGBge3J9CnByaW50Y3AodHJlZV9tb2RlbF9kYXRhLCBkaWdpdHMgPSAzKQpgYGAKYGBge3J9CnN1bW1hcnkodHJlZV9tb2RlbF9kYXRhKQoKdmFyX2ltcF9wbG90KGxhcmdlX3RyZWVfbW9kZWwsIDIwKQpgYGAKCiMjIENyb3NzLXZhbGlkYXRpb24KCmBgYHtyfQpvdXRlciA8LSBtYWtlUmVzYW1wbGVEZXNjKCJDViIsIGl0ZXJzID0gNSkKdHJlZV93cmFwcGVyIDwtIG1ha2VUdW5lV3JhcHBlcigiY2xhc3NpZi5ycGFydCIsIHJlc2FtcGxpbmcgPSBjdl9mb3JfdHVuaW5nLAogICAgICAgICAgICAgICAgICAgICAgICAgICAgICAgcGFyLnNldCA9IHRyZWVfcGFyYW1ldGVyX3NwYWNlLAogICAgICAgICAgICAgICAgICAgICAgICAgICAgICAgY29udHJvbCA9IHJhbmRfc2VhcmNoKQpwYXJhbGxlbFN0YXJ0U29ja2V0KGNwdXMgPSBkZXRlY3RDb3JlcygpKQpjdl93aXRoX3R1bmluZyA8LSByZXNhbXBsZSh0cmVlX3dyYXBwZXIsIHNldmVyaXR5X3Rhc2ssIHJlc2FtcGxpbmcgPSBvdXRlcikKcGFyYWxsZWxTdG9wKCkKY3Zfd2l0aF90dW5pbmcKYGBgCg==
