# Supplementary material for: Mood Disorder Detection in Adolescents by Classification Trees, Random Forests and XGBoost in Presence of Missing Data
Source: Entropy (Basel). 2021 Sep 14;23(9):1210. doi: 10.3390/e23091210 (PMC8468933; doi:10.3390/e23091210)
Supplement: Supplementary file 1 [file entropy-23-01210-s001.zip › social_AI/random_forest.html]

R Notebook


# R Notebook

# Random Forest - Bagging

```
library(mlr)
```

```
## Loading required package: ParamHelpers
```

```
## Warning message: 'mlr' is in 'maintenance-only' mode since July 2019.
## Future development will only happen in 'mlr3'
## (<https://mlr3.mlr-org.com>). Due to the focus on 'mlr3' there might be
## uncaught bugs meanwhile in {mlr} - please consider switching.
```

```
library(magrittr)
library(tidyverse)
```

```
## ── Attaching packages ─────────────────────────────────────── tidyverse 1.3.0 ──
```

```
## ✔ ggplot2 3.3.3     ✔ purrr   0.3.4
## ✔ tibble  3.1.0     ✔ dplyr   1.0.5
## ✔ tidyr   1.1.3     ✔ stringr 1.4.0
## ✔ readr   1.4.0     ✔ forcats 0.5.1
```

```
## ── Conflicts ────────────────────────────────────────── tidyverse_conflicts() ──
## ✖ tidyr::extract()   masks magrittr::extract()
## ✖ dplyr::filter()    masks stats::filter()
## ✖ dplyr::lag()       masks stats::lag()
## ✖ purrr::set_names() masks magrittr::set_names()
```

```
library(randomForest)
```

```
## randomForest 4.6-14
```

```
## Type rfNews() to see new features/changes/bug fixes.
```

```
## 
## Attaching package: 'randomForest'
```

```
## The following object is masked from 'package:dplyr':
## 
##     combine
```

```
## The following object is masked from 'package:ggplot2':
## 
##     margin
```

```
#' Function imputing data with the use of random forest
#' @param data_frame data frame with both predictors and independent data
impute_NAs <- function(data_frame, ntree = 500) {
  transformed_data <- rfImpute(data.matrix(data_frame[, -1]), as.factor(data_frame[["severity"]]), iter=5, ntree=ntree)
  transformed_data[, 1] <- transformed_data[, 1]-1
  transformed_data %<>% 
    as.data.frame()
  names(transformed_data)[[1]] <- 'severity'
  transformed_data$severity <- as.factor(transformed_data$severity)
  transformed_data
}
```

## Data

```
mood_data_train_class <- readRDS("mood_train.RDS")
#mood_data_test_class <- readRDS("mood_test.RDS")
# mood_data_test_full <- readRDS("mood_test_full.RDS")
```

imputing missing values

```
mood_data_train_class_matrix <- impute_NAs(mood_data_train_class)
```

```
## ntree      OOB      1      2
##   500:   6.30%  0.20% 91.59%
## ntree      OOB      1      2
##   500:   6.67%  0.40% 94.39%
## ntree      OOB      1      2
##   500:   6.30%  0.20% 91.59%
## ntree      OOB      1      2
##   500:   6.73%  0.40% 95.33%
## ntree      OOB      1      2
##   500:   6.55%  0.27% 94.39%
```

## Make task and learner

```
severity_forest <- makeLearner("classif.randomForest")
severity_task <- makeClassifTask(data = mood_data_train_class_matrix, target = "severity")
```

## Tuning the random forest hyperparameters

```
forest_param_space <- makeParamSet(
  makeIntegerParam("ntree", lower = 500, upper = 500), # fixing the number of trees to 500
  makeIntegerParam("mtry", lower = 6, upper = 12),
  makeIntegerParam("nodesize", lower = 1, upper = 5),
  makeIntegerParam("maxnodes", lower = 5, upper = 20)
)

random_search <- makeTuneControlRandom(maxit = 100)
cv_for_tuning <- makeResampleDesc("CV", iters = 5)


library(parallel)
library(parallelMap)
print(paste("Detected ", detectCores(), " cores", collapse = ""))
```

```
## [1] "Detected  16  cores"
```

```
parallelStartSocket(cpus = detectCores())
```

```
## Starting parallelization in mode=socket with cpus=16.
```

```
tuned_forest_pars <- tuneParams(severity_forest, task = severity_task,
                              resampling = cv_for_tuning, 
                              par.set = forest_param_space,
                              control = random_search)
```

```
## [Tune] Started tuning learner classif.randomForest for parameter set:
```

```
##             Type len Def     Constr Req Tunable Trafo
## ntree    integer   -   - 500 to 500   -    TRUE     -
## mtry     integer   -   -    6 to 12   -    TRUE     -
## nodesize integer   -   -     1 to 5   -    TRUE     -
## maxnodes integer   -   -    5 to 20   -    TRUE     -
```

```
## With control class: TuneControlRandom
```

```
## Imputation value: 1
```

```
## Exporting objects to slaves for mode socket: .mlr.slave.options
```

```
## Mapping in parallel: mode = socket; level = mlr.tuneParams; cpus = 16; elements = 100.
```

```
## [Tune] Result: ntree=500; mtry=10; nodesize=1; maxnodes=16 : mmce.test.mean=0.0660962
```

```
parallelStop()
```

```
## Stopped parallelization. All cleaned up.
```

```
tuned_forest_pars
```

```
## Tune result:
## Op. pars: ntree=500; mtry=10; nodesize=1; maxnodes=16
## mmce.test.mean=0.0660962
```

## Train the final model with tuned parameters

```
tuned_forest <- setHyperPars(severity_forest, par.vals = tuned_forest_pars$x)
tuned_forest_model <- train(tuned_forest, severity_task)
saveRDS(tuned_forest_model, "tuned_forest_model.RDS")
```

## Plotting the out-of-bag error

```
forest_model_data <- getLearnerModel(tuned_forest_model)
res <- colnames(forest_model_data$err.rate)
plot(forest_model_data, col = 1:length(res), lty = 1:length(res))
legend("topright", res,
       col = 1:length(res),
       lty = 1:length(res))
```

We can see that once we hit 100 trees the error estimate stabilizes - this indicates that we have enough trees in our forest (we could have had fewer)

## Corss-validating the model building process (including hyperparameters)

```
outer <- makeResampleDesc("CV", iters = 5)
forest_wrapper <- makeTuneWrapper("classif.randomForest",
                                 resampling = cv_for_tuning,
                                 par.set = forest_param_space,
                                 control = random_search)
parallelStartSocket(cpus = detectCores())
```

```
## Starting parallelization in mode=socket with cpus=16.
```

```
cv_with_tuning <- resample(forest_wrapper, severity_task, resampling = outer)
```

```
## Exporting objects to slaves for mode socket: .mlr.slave.options
```

```
## Resampling: cross-validation
```

```
## Measures:             mmce
```

```
## Mapping in parallel: mode = socket; level = mlr.resample; cpus = 16; elements = 5.
```

```
##
```

```
## Aggregated Result: mmce.test.mean=0.0673384
```

```
##
```

```
parallelStop()
```

```
## Stopped parallelization. All cleaned up.
```

```
cv_with_tuning
```

```
## Resample Result
## Task: mood_data_train_class_matrix
## Learner: classif.randomForest.tuned
## Aggr perf: mmce.test.mean=0.0673384
## Runtime: 3015.38
```
