# Supplementary material for: Mood Disorder Detection in Adolescents by Classification Trees, Random Forests and XGBoost in Presence of Missing Data
Source: Entropy (Basel). 2021 Sep 14;23(9):1210. doi: 10.3390/e23091210 (PMC8468933; doi:10.3390/e23091210)
Supplement: Supplementary file 1 [file entropy-23-01210-s001.zip › social_AI/models_performance.html]

R Notebook


# R Notebook

```
library(mlr)
```

```
## Loading required package: ParamHelpers
```

```
## Warning message: 'mlr' is in 'maintenance-only' mode since July 2019.
## Future development will only happen in 'mlr3'
## (<https://mlr3.mlr-org.com>). Due to the focus on 'mlr3' there might be
## uncaught bugs meanwhile in {mlr} - please consider switching.
```

```
library(magrittr)
library(tidyverse)
```

```
## ── Attaching packages ─────────────────────────────────────── tidyverse 1.3.1 ──
```

```
## ✓ ggplot2 3.3.3     ✓ purrr   0.3.4
## ✓ tibble  3.1.1     ✓ dplyr   1.0.5
## ✓ tidyr   1.1.3     ✓ stringr 1.4.0
## ✓ readr   1.4.0     ✓ forcats 0.5.1
```

```
## ── Conflicts ────────────────────────────────────────── tidyverse_conflicts() ──
## x tidyr::extract()   masks magrittr::extract()
## x dplyr::filter()    masks stats::filter()
## x dplyr::lag()       masks stats::lag()
## x purrr::set_names() masks magrittr::set_names()
```

```
library(randomForest)
```

```
## randomForest 4.6-14
```

```
## Type rfNews() to see new features/changes/bug fixes.
```

```
## 
## Attaching package: 'randomForest'
```

```
## The following object is masked from 'package:dplyr':
## 
##     combine
```

```
## The following object is masked from 'package:ggplot2':
## 
##     margin
```

```
library(xgboost)
```

```
## 
## Attaching package: 'xgboost'
```

```
## The following object is masked from 'package:dplyr':
## 
##     slice
```

```
library(caret)
```

```
## Loading required package: lattice
```

```
## 
## Attaching package: 'caret'
```

```
## The following object is masked from 'package:purrr':
## 
##     lift
```

```
## The following object is masked from 'package:mlr':
## 
##     train
```

## Models performance

## Loading test data

```
test_data <- readRDS("mood_test.RDS")
#' Function imputing data with the use of random forest
#' @param data_frame data frame with both predictors and independent data
impute_NAs <- function(data_frame, ntree = 500) {
  transformed_data <- rfImpute(data.matrix(data_frame[, -1]), as.factor(data_frame[["severity"]]), iter=5, ntree=ntree)
  transformed_data[, 1] <- transformed_data[, 1]-1
  transformed_data %<>% 
    as.data.frame()
  names(transformed_data)[[1]] <- 'severity'
  transformed_data$severity <- as.factor(transformed_data$severity)
  transformed_data
}
```

## Loading trained models

```
tuned_tree_model <- readRDS('tuned_tree_model.RDS')
tuned_forest_model <- readRDS('tuned_forest_model.RDS')
tuned_xgb_model <- readRDS('tuned_xgb_model.RDS')
```

## Tree model by the rpart algorithm

```
predicted_mood_tree <- predict(tuned_tree_model, newdata = test_data)
```

```
## Warning in predict.WrappedModel(tuned_tree_model, newdata = test_data): Provided
## data for prediction is not a pure data.frame but from class tbl_df, hence it
## will be converted.
```

```
performance <- performance(predicted_mood_tree, measures = list(mmce, acc))
performance
```

```
##       mmce        acc 
## 0.03074013 0.9692599
```

```
calculateConfusionMatrix(predicted_mood_tree)
```

```
##         predicted
## true     FALSE TRUE -err.-
##   FALSE    632    7      7
##   TRUE      14   30     14
##   -err.-    14    7     21
```

# Tree random forest

```
# in the course of building the training model, the factors got rewritten
#mood_data_test_class_matrix <- data.matrix(test_data) %>% 
#  na.roughfix() 

mood_data_test_class_matrix <- impute_NAs(test_data)
```

```
## ntree      OOB      1      2
##   500:   6.59%  0.47% 95.45%
## ntree      OOB      1      2
##   500:   6.44%  0.31% 95.45%
## ntree      OOB      1      2
##   500:   6.44%  0.31% 95.45%
## ntree      OOB      1      2
##   500:   6.44%  0.31% 95.45%
## ntree      OOB      1      2
##   500:   6.59%  0.31% 97.73%
```

```
predicted_forest_tree <- predict(tuned_forest_model, newdata = mood_data_test_class_matrix)
performance <- performance(predicted_forest_tree, measures = list(mmce, acc))
performance
```

```
##       mmce        acc 
## 0.06442167 0.93557833
```

```
calculateConfusionMatrix(predicted_forest_tree)
```

```
##         predicted
## true       0 1 -err.-
##   0      639 0      0
##   1       44 0     44
##   -err.-  44 0     44
```

# xgb model

```
predicted_mood_xgb <- predict(tuned_xgb_model, newdata = mood_data_test_class_matrix)
performance <- performance(predicted_mood_xgb, measures = list(mmce, acc))
performance
```

```
##       mmce        acc 
## 0.06149341 0.93850659
```

```
calculateConfusionMatrix(predicted_mood_xgb)
```

```
##         predicted
## true       0 1 -err.-
##   0      634 5      5
##   1       37 7     37
##   -err.-  37 5     42
```

# Assessing the quality of classifying the most severe cases

```
mood_test_full <- readRDS('mood_test_full.RDS')
```

## Tree

### most severe

```
predicted_mood_tree$data$response[mood_test_full$mood == 6] %>% as.logical() %>% sum() / sum(mood_test_full$mood == 6) * 100
```

```
## [1] 75
```

### less severe

```
predicted_mood_tree$data$response[mood_test_full$mood == 5] %>% as.logical() %>% sum() / sum(mood_test_full$mood == 5) * 100
```

```
## [1] 66.66667
```

## xgboost

### most severe

```
predicted_mood_xgb$data$response[mood_test_full$mood == 6] %>% as.numeric %>% subtract(1) %>% sum() / sum(mood_test_full$mood == 6, na.rm = TRUE) * 100
```

```
## [1] 12.5
```

### less severe

```
predicted_mood_xgb$data$response[mood_test_full$mood == 5] %>% as.numeric %>% subtract(1) %>% sum() / sum(mood_test_full$mood == 5, na.rm = TRUE) * 100
```

```
## [1] 16.66667
```
