# Supplementary material for: Mood Disorder Detection in Adolescents by Classification Trees, Random Forests and XGBoost in Presence of Missing Data
Source: Entropy (Basel). 2021 Sep 14;23(9):1210. doi: 10.3390/e23091210 (PMC8468933; doi:10.3390/e23091210)
Supplement: Supplementary file 1 [file entropy-23-01210-s001.zip › social_AI/social_AI.html]

social\_AI


# social\_AI

#### Jarosław Piskorski

#### 29 05 2021

```
library(mlr)
```

```
## Loading required package: ParamHelpers
```

```
## Warning message: 'mlr' is in 'maintenance-only' mode since July 2019.
## Future development will only happen in 'mlr3'
## (<https://mlr3.mlr-org.com>). Due to the focus on 'mlr3' there might be
## uncaught bugs meanwhile in {mlr} - please consider switching.
```

```
library(magrittr)
library(tidyverse)
```

```
## ── Attaching packages ─────────────────────────────────────── tidyverse 1.3.1 ──
```

```
## ✓ ggplot2 3.3.3     ✓ purrr   0.3.4
## ✓ tibble  3.1.1     ✓ dplyr   1.0.5
## ✓ tidyr   1.1.3     ✓ stringr 1.4.0
## ✓ readr   1.4.0     ✓ forcats 0.5.1
```

```
## ── Conflicts ────────────────────────────────────────── tidyverse_conflicts() ──
## x tidyr::extract()   masks magrittr::extract()
## x dplyr::filter()    masks stats::filter()
## x dplyr::lag()       masks stats::lag()
## x purrr::set_names() masks magrittr::set_names()
```

# Data Preparation

```
library(foreign)
# dane1 <- read.spss("dane.sav", to.data.frame=T) %>% 
#   as_tibble()
# spss_data <- read.spss("uczniowie-samopoczucie.sav", to.data.frame=T) %>% 
#  as_tibble()
# saveRDS(file = "spss_data.rds", spss_data)
#' Function filling in a column in a transformed data frame
#' @param levels list - list with levels and their numeric representations
#' @template_column  the column that is being mapped to 
fill_column <- function(levels_list, template_column) {
  result_column <- rep(NA, length(template_column))
  for (level in names(levels_list)) {
    level_type <- template_column == level
    if (sum(level_type, na.rm = TRUE) > 0) {
      result_column <- levels_list[[level]]
    }
  }
  result_column
}

#' Function changing the factor names from tak-nie to yes-no
#' @param factor_vector vector of tak-nie factors
yes_no_relevel <- function(factor_vector) {
  levels(factor_vector) <- c("yes", "no")
  factor_vector
}

#' Function recoding a factorial variable as one-hot
#' @param factor_vector factor vector
#' @param factor_name factor name
one_hot_from_factor <- function(factor_vector, factor_name) {
  sapply(factor_vector, function(elem) {
    if(is.na(elem)) {
      return(NA)
    }
    'if'(elem == factor_name, 1, 0)
  })
}

 fill_column_from_many <- function(list_of_factor_vectors, factor_name) {
   interm_tib <- as.data.frame(list_of_factor_vectors)
   interm_tib %>% apply(1, function(row) {
     if (all(is.na(row))) {
       return(NA)
     }
     if (factor_name %in% row) {
       return(1)
     } else{
       return(0)
     }
   })
 }

#' Function filling in a number of columns with numeric values instead of ordered factors and returning a dataframe - useful for averaging across columns
#' @param col_list list of columns (vectors) to be filled
#' @param levels_list list with mapping between levels names and values
fill_and_collect_columns <- function(col_list, levels_list) {
  result <- data.frame(fill_column(levels_list = levels_list, template_column = col_list[[1]]))
  for (idx in 2:length(col_list)) {
    result <- cbind(result, fill_column(levels_list = levels_list, template_column = col_list))
  }
  result
}
spss_data <- readRDS("spss_data.rds") # around 3.8 Mb
```

## Mood column

The mood column contains the following ordered factors: 1. No depressed mood (1) 2. Normal state but there is no joy (2) 3. Mildly depressed mood (3) 4. Average level of depressed mood (4) 5. Strongly depressed mood (5) 6. Very strongly depressed mood (6)

This column has a clear numeric representation - the schools are ordered from least depressed to the most depressed. The numeric representation is given in parentheses next to the factor in the list above.

```
 # this tibble will be filled in as we go
mood <- list(
  "BD"  = 1,
  "normalny s" = 2, 
  "lagodna" = 3,
  "umiarkowana" = 4,
  "ciężka" = 5, 
  "b. ciężka" = 6)
mood_data <- tibble(mood = fill_column(levels_list = mood, 
                           template_column = spss_data$su))
```

Number of NAs in the mood column

```
is.na(mood_data$mood) %>% sum
```

```
## [1] 221
```

## Question 1: In what type of school do you want to continue learning

1. Regular secondary school (5)
2. Technical secondary school (4)
3. Vocational school (3)
4. Vocational training center (2)
5. No plans for further learning (2)

This column has a clear numeric representation - the schools are ordered from the most difficult, to the least difficult (or perhaps from the most ambitious to the least ambitious). The numeric representation is given in parentheses next to the factor in the list above.

```
schools <- list(
  "  W liceum ogólnokształcącym  " = 5,
  "  W technikum  " = 4,   
  "  W zasadniczej szkole zawodowej  " = 3, 
  "  W ośrodku doskonalenia zawodowego  " = 2,
  "  Nie planuję dalej uczyć się  " = 1
)

Q1_further_learning <- fill_column(levels_list = schools, 
                                template_column = spss_data$z1)
mood_data %<>% 
  add_column(Q1_further_learning)
```

## Question 2: Do you think you will be able to carry out this plan (the school choice)

1. Yes (4)
2. Perhaps (3)
3. Don’t think so (2)
4. No (1)

This column has a clear numeric representation - the schools are ordered from the most to least certain. The numeric representation is given in parentheses next to the factor in the list above.

```
school_plan_real <- list(
   "  tak  "  = 4,
   "  raczej tak  " = 3,
   "  raczej nie  " = 2,
   "  nie  " = 1
)

Q2_school_plan_real <- fill_column(levels_list = school_plan_real, 
                                template_column = spss_data$z2)
mood_data %<>% 
  add_column(Q2_school_plan_real)
```

## Question 3: If you are unsure of your further education, what are your fears connected with - choose all that apply:

1. I don’t know if I get admitted to my preferred school because of the exam results
2. I don’t know if my parents agree to my preferred school.
3. I don’t know if my parents will be able to cover the cost of learning
4. I suspect I’ll have to start working to help support my family
5. I have no fears about my preferred school
6. Other reasons

This variable is purely categorical - it will be encoded with one-hot encoding. The coded variables use Polish tak/nie for yes/no, so this is being corrected

```
Q3_edu_fear_1_exam <- yes_no_relevel(spss_data$z3_1)
Q3_edu_fear_2_consent <- yes_no_relevel(spss_data$z3_2)
Q3_edu_fear_3_money <- yes_no_relevel(spss_data$z3_3)
Q3_edu_fear_4_work <- yes_no_relevel(spss_data$z3_4)
Q3_edu_fear_5_none <- yes_no_relevel(spss_data$z3_5)
Q3_edu_fear_6_other <- yes_no_relevel(spss_data$z3_6)
mood_data %<>% 
  add_column(Q3_edu_fear_1_exam) %>% 
  add_column(Q3_edu_fear_2_consent) %>% 
  add_column(Q3_edu_fear_3_money) %>% 
  add_column(Q3_edu_fear_4_work) %>% 
  add_column(Q3_edu_fear_5_none) %>% 
  add_column(Q3_edu_fear_6_other)
```

## Question 4: skipped - open ended question

## Question 5: I am raised by:

1. Both parents
2. By mother only
3. By father only
4. By another family member
5. Outside a family

This is a categorical variable - will be encoded by one hot encoding. In the original dataset it is a factorial variable

```
Q5_raised_by_both <- one_hot_from_factor(spss_data$z5, "  Jestem wychowywany przez oboje rodziców  ")
Q5_raised_by_mother <- one_hot_from_factor(spss_data$z5, "  Jestem wychowywany/a tylko przez matkę  ")
Q5_raised_by_father <- one_hot_from_factor(spss_data$z5, "  Jestem wychowywany/a tylko przez ojca  ")
Q5_raised_by_other <- one_hot_from_factor(spss_data$z5, "  Jestem wychowywany/a przez inne osoby z rodziny  ")
Q5_raised_outside_family <- one_hot_from_factor(spss_data$z5, "  Wychowuję się poza Q5_rodziną  ")

mood_data %<>% 
  add_column(Q5_raised_by_both) %>% 
  add_column(Q5_raised_by_mother) %>% 
  add_column(Q5_raised_by_father) %>% 
  add_column(Q5_raised_by_other) %>% 
  add_column(Q5_raised_outside_family)
```

## Question 6: What is the professional situation of your parents(1 - works in the country, 2 - works abroad, 3 - does not work)

1. Mother
2. Father

This is a situation, when one question is represented by two categorical variables - this will be encoded as a series of one-hot vectors.

```
Q6_mother_works_domestically <- one_hot_from_factor(spss_data$z6_1, "  pracuje w kraju  ")
Q6_mother_works_abroad <- one_hot_from_factor(spss_data$z6_1, "  pracuje za granicą  ")
Q6_mother_does_not_work <- one_hot_from_factor(spss_data$z6_1, "  nie pracuje w ogóle  ")
Q6_father_works_domestically <- one_hot_from_factor(spss_data$z6_2, "  pracuje w kraju  ")
Q6_father_works_abroad <- one_hot_from_factor(spss_data$z6_2, "  pracuje za granicą  ")
Q6_father_does_not_work <- one_hot_from_factor(spss_data$z6_2, "  nie pracuje w ogóle  ")
mood_data %<>% 
  add_column(Q6_mother_works_domestically) %>% 
  add_column(Q6_mother_works_abroad) %>% 
  add_column(Q6_mother_does_not_work) %>% 
  add_column(Q6_father_works_domestically) %>% 
  add_column(Q6_father_works_abroad) %>% 
  add_column(Q6_father_does_not_work)
```

## Question 7: What is your assessment of the financial situation of your family?

1. We do not want for anything - everyone can afford what they want. (5)
2. We have everything we need, but we live frugally. (4)
3. We sometimes lack money for food, rent or clothes - we live very frugally, there is no money for fun, but we cope. (3)
4. We often lack money for food, rent or clothes, we often borrow money, but we always pay our debts. (2)
5. We often lack money for food, rent or clothes, we are helped by social workers and other people. (1)

This column has a clear numeric representation - from the best situation to the worst. . The numeric representation is given in parentheses next to the factor in the list above.

```
financial_situation <- list(
  "  W moim domu jest wszystko, nigdy niczego nie brakuje  " = 5,          
  "  W moim domu jest wszystko ale żyjemy skromnie  " = 4,        
  "  W moim domu czasami brakuje pieniędzy, ale jakoś sobie radzimy  " = 3,
  "  W moim domu często brakuje pieniędzy, ale długi oddajemy  " = 2,
  "  W moim domu brakuje pieniędzy, pomaga nam pomoc społeczna  "  = 1
)

Q7_financial_situation <- fill_column(levels_list = financial_situation,
                                   template_column = spss_data$z7)
mood_data %<>% 
  add_column(Q7_financial_situation)
```

## Question 8: What are the major concerns of your family:

1. Unemployment of both parents
2. Unemployment of one parent
3. Low income of parent/parents
4. Family conflicts
5. Alcohol abuse
6. Debt
7. Bad housing situation
8. Lack of money for basic needs (food, clothing, school textbooks and accessories)
9. One or both parents work too much
10. We don’t spent free time together
11. Caring for a chronically ill or handicapped person
12. There are no concerns

This variable is purely categorical - it will be encoded with one-hot encoding. The coded variables use Polish tak/nie for yes/no, so this is being corrected

```
Q8_family_concern_1_unemp_2 <- yes_no_relevel(spss_data$z8_a)
Q8_family_concern_2_unemp_2 <- yes_no_relevel(spss_data$z8_b)
Q8_family_concern_3_income <- yes_no_relevel(spss_data$z8_c)
Q8_family_concern_4_conflicts <- yes_no_relevel(spss_data$z8_d)
Q8_family_concern_5_alcohol <- yes_no_relevel(spss_data$z8_e)
Q8_family_concern_6_debt <- yes_no_relevel(spss_data$z8_f)
# family_concern_7_housing <- yes_no_relevel(spss_data$z8_g) - no data available
Q8_family_concern_8_money <- yes_no_relevel(spss_data$z8_h)
Q8_family_concern_9_long_work <- yes_no_relevel(spss_data$z8_i)
Q8_family_concern_10_time <- yes_no_relevel(spss_data$z8_j)
Q8_family_concern_11_handicap <- yes_no_relevel(spss_data$z8_k)
Q8_family_concern_12_no <- yes_no_relevel(spss_data$z8_l)

mood_data %<>% 
  add_column(Q8_family_concern_1_unemp_2) %>% 
  add_column(Q8_family_concern_2_unemp_2) %>% 
  add_column(Q8_family_concern_3_income) %>% 
  add_column(Q8_family_concern_4_conflicts) %>% 
  add_column(Q8_family_concern_5_alcohol) %>% 
  add_column(Q8_family_concern_6_debt) %>% 
  #add_column(family_concern_7_housing) %>% 
  add_column(Q8_family_concern_8_money) %>% 
  add_column(Q8_family_concern_9_long_work) %>% 
  add_column(Q8_family_concern_10_time) %>% 
  add_column(Q8_family_concern_11_handicap) %>% 
  add_column(Q8_family_concern_12_no)
```

## Question 9: Do you help your family in solving their problems? If so, how?

1. I try to save money. I pass my own pleasures so that my parents don’t have to spend money on me.
2. I help out at home so that the parents can work more.
3. I help my parents in their professional work (e.g. running a shop, helping out on a farm etc.)
4. I work myself
5. I don’t have to help as we have no problems
6. Others

This variable is purely categorical - it will be encoded with one-hot encoding. The coded variables use Polish tak/nie for yes/no, so this is being corrected

```
Q9_help_1_save <- yes_no_relevel(spss_data$z9_a)
Q9_help_2_home <- yes_no_relevel(spss_data$z9_b)
Q9_help_3_at_work <- yes_no_relevel(spss_data$z9_c)
Q9_help_4_work <- yes_no_relevel(spss_data$z9_d)
Q9_help_5_no_problems <- yes_no_relevel(spss_data$z9_e)
Q9_help_6_other <- yes_no_relevel(spss_data$z9_f)

mood_data %<>% 
  add_column(Q9_help_1_save) %>% 
  add_column(Q9_help_2_home) %>% 
  add_column(Q9_help_3_at_work) %>% 
  add_column(Q9_help_4_work) %>% 
  add_column(Q9_help_5_no_problems) %>% 
  add_column(Q9_help_6_other)
```

## Question 10: If you chose 4 in question 9, what type of work do you do?

1. Small jobs (cleaning, babysitting, loading etc.)
2. Ads distribion
3. Promotion/sales (e.g. in a shop)
4. I ask others for money
5. Collecting berries, mushrooms etc.
6. Selling the above
7. I take part in seasonal work on a farm
8. Car wash, parking staff
9. Others

This variable is purely categorical - since this is a very specific question, all answers will be summarized by the number of “yes” answers. The coded variables use Polish tak/nie for yes/no, so this is being corrected.

```
Q10_work_1_small <- yes_no_relevel(spss_data$z10_a)
Q10_work_2_ads <- yes_no_relevel(spss_data$z10_b)
Q10_work_3_sales <- yes_no_relevel(spss_data$z10_c)
Q10_work_4_beg <- yes_no_relevel(spss_data$z10_d)
Q10_work_5_collect <- yes_no_relevel(spss_data$z10_e)
Q10_work_6_sell <- yes_no_relevel(spss_data$z10_f)
Q10_work_7_seasonal <- yes_no_relevel(spss_data$z10_g)
Q10_work_8_carwash <- yes_no_relevel(spss_data$z10_h)
Q10_work_9_others <- yes_no_relevel(spss_data$z10_i)

Q10_work <- apply(data.frame(Q10_work_1_small,
                             Q10_work_2_ads,
                             Q10_work_3_sales,
                             Q10_work_4_beg,
                             Q10_work_5_collect,
                             Q10_work_6_sell,
                             Q10_work_7_seasonal,
                             Q10_work_8_carwash,
                             Q10_work_9_others), 1, function(row) sum(row == "yes", na.rm = TRUE)) 

mood_data %<>% 
  add_column(Q10_work)
```

## Question 11: Who initiated taking up the above work?

1. Parents demanded it
2. I feel I should
3. It was my own decission to have more pocket money
4. My friends
5. Others

This is a purely categorical variable. It will be encoded with one-hot encoding.

```
Q11_work_by_parents <- one_hot_from_factor(spss_data$z11, "  Wymagają tego ode mnie rodzice  ")
Q11_work_I_should <- one_hot_from_factor(spss_data$z11, "  Czuję się w obowiązku wspomóc finansowo rodzinę  ")
Q11_work_own_decission <- one_hot_from_factor(spss_data$z11, "  Z własnej inicjatywy  " )
Q11_work_friends <- one_hot_from_factor(spss_data$z11, "  Z inicjatywy kolegów i koleżanek  ")
Q11_work_others <- one_hot_from_factor(spss_data$z11,  "  Inne")

mood_data %<>% 
  add_column(Q11_work_by_parents) %>% 
  add_column(Q11_work_I_should) %>% 
  add_column(Q11_work_own_decission) %>% 
  add_column(Q11_work_friends) %>% 
  add_column(Q11_work_others)
```

## Question 12: If you selected 4 in question 9, how do you spend the money you earn?

1. I give it to my parents
2. I give some to my parents and keep some
3. I use it to support myself (bus tickets, clothes, food)
4. I spend it all on the thinks I like (fun, cinema etc.)
5. Both for things I like and to support myself

This is a purely categorical variable. It will be encoded with one-hot encoding.

```
Q12_give_money_to_parents <- one_hot_from_factor(spss_data$z12, "  W całości oddaję rodzicom  ")
Q12_keep_some_give_some <-one_hot_from_factor(spss_data$z12, "  Część oddaję rodzicom  ") 
Q12_support_myself <- one_hot_from_factor(spss_data$z12,"  W całości na swoje utrzymanie  ")
Q12_pleasures <- one_hot_from_factor(spss_data$z12, "  W całości na swoje przyjemności  " )
Q12_peasures_support <- one_hot_from_factor(spss_data$z12, "  Na swoje utrzymanie i swoje przyjemności  ")

mood_data %<>% 
  add_column(Q12_give_money_to_parents) %>% 
  add_column(Q12_keep_some_give_some) %>% 
  add_column(Q12_support_myself) %>% 
  add_column(Q12_pleasures) %>% 
  add_column(Q12_peasures_support)
```

## Question 13: Rate each of the statements below according to how well they fit the reality of your relation with your parents. Use the scale 1-5, where 1 means fully true and 5 means not ture at all.

1. I feel my parents are happy with me
2. Sometimes I feel my parents do not notice me
3. I feel my parents have no interest in my life
4. I can always count on my parents if I’m in trouble
5. I sometimes wonder if my parents really love me
6. My parents devote little time to me
7. I feel I am close to my parents
8. It is hard for me to talk to my parents
9. I feel I am important to my parents
10. I feel my parents do not understand me

These are all different numerical vectors coded as separate columns.

```
rating <- list(
  "  Prawdziwe  " = 1,           
  "  Raczej prawdziwe  " = 2, 
  "  Trudno powiedzieć  " = 3,
  "  Raczej nieprawdziwe  " = 4, 
  "  Nieprawdziwe  "  = 5
)
Q13_1_happy <- fill_column(levels_list = rating, template_column = spss_data$z13_a)
Q13_2_not_notice <- fill_column(levels_list = rating, template_column = spss_data$z13_b)
Q13_3_not_interested <- fill_column(levels_list = rating, template_column = spss_data$z13_c)
Q13_4_can_count <- fill_column(levels_list = rating, template_column = spss_data$z13_d)
Q13_5_if_love <- fill_column(levels_list = rating, template_column = spss_data$z13_e)
Q13_6_time <- fill_column(levels_list = rating, template_column = spss_data$z13_f)
Q13_7_close <- fill_column(levels_list = rating, template_column = spss_data$z13_g)
Q13_8_difficult_talk <- fill_column(levels_list = rating, template_column = spss_data$z13_h)
Q13_9_important <- fill_column(levels_list = rating, template_column = spss_data$z13_i)
Q13_10_important <- fill_column(levels_list = rating, template_column = spss_data$z13_j)

mood_data %<>% 
  add_column(Q13_1_happy) %>% 
  add_column(Q13_2_not_notice) %>% 
  add_column(Q13_3_not_interested) %>% 
  add_column(Q13_4_can_count) %>% 
  add_column(Q13_5_if_love) %>% 
  add_column(Q13_6_time) %>% 
  add_column(Q13_7_close) %>% 
  add_column(Q13_8_difficult_talk) %>% 
  add_column(Q13_9_important) %>% 
  add_column(Q13_10_important)
```

## Question 14: Young people sometimes feel lonely in families. What do you most often do if you feel lonely at home. Choose up to 3 answers

1. I listen to music
2. I watch television
3. I play computer games
4. I surf the internet
5. I talk to friends over communicators
6. I write on my own blog about how I feel
7. I go outside for a walk
8. I go out and meet with my frieds
9. I use alcohol
10. I use drugs
11. I use tobacco
12. Other
13. I don’t do anything
14. I never feel lonely in my family

This variable will be coded as one-hot encoding, with each column corresponding to one choice. If a respondent selected this choice, the value will be 1, otherwise it will be 0 or NA if not available.

```
Q14_1_music <- fill_column_from_many(list(spss_data$z14_1, spss_data$z14_2, spss_data$z14_3), "  Słucham muzyki  " )
Q14_2_TV <- fill_column_from_many(list(spss_data$z14_1, spss_data$z14_2, spss_data$z14_3), "  Oglądam telewizję  "  )
Q14_3_computer_games <- fill_column_from_many(list(spss_data$z14_1, spss_data$z14_2, spss_data$z14_3), "  Gram w gry komputerowe  ")
Q14_4_surf_I <- fill_column_from_many(list(spss_data$z14_1, spss_data$z14_2, spss_data$z14_3), "  Surfuję po Internecie  ")
Q15_5_talk <- fill_column_from_many(list(spss_data$z14_1, spss_data$z14_2, spss_data$z14_3),"  Rozmawiam ze znajomymi na GG, czacie  ")
Q15_6_talk <- fill_column_from_many(list(spss_data$z14_1, spss_data$z14_2, spss_data$z14_3),"  Piszę co myślę na własnym blogu  " )
Q15_7_walk <- fill_column_from_many(list(spss_data$z14_1, spss_data$z14_2, spss_data$z14_3),"  Wychodzę z domu i spaceruję  "  )
Q15_8_meet <- fill_column_from_many(list(spss_data$z14_1, spss_data$z14_2, spss_data$z14_3),"  Wychodzę z domu i spotykam się z kolegami")
Q15_9_alcohol <- fill_column_from_many(list(spss_data$z14_1, spss_data$z14_2, spss_data$z14_3),"Sięgam po alkohol")
Q15_10_drugs <- fill_column_from_many(list(spss_data$z14_1, spss_data$z14_2, spss_data$z14_3), "Sięgam po narkotyki")
Q15_11_tobacco <- fill_column_from_many(list(spss_data$z14_1, spss_data$z14_2, spss_data$z14_3), "Sięgam po papierosy" )
Q15_12_other <- fill_column_from_many(list(spss_data$z14_1, spss_data$z14_2, spss_data$z14_3), "Inne" )
Q15_13_nothing <- fill_column_from_many(list(spss_data$z14_1, spss_data$z14_2, spss_data$z14_3), "Nic nie robię" )
Q15_14_never_lonely <- fill_column_from_many(list(spss_data$z14_1, spss_data$z14_2, spss_data$z14_3), "Nigdy nie odczuwam osamotnienia w rodzinie")

mood_data %<>% 
  add_column(Q14_1_music) %>% 
  add_column(Q14_2_TV) %>% 
  add_column(Q14_3_computer_games) %>% 
  add_column(Q14_4_surf_I) %>% 
  add_column(Q15_5_talk) %>% 
  add_column(Q15_6_talk) %>% 
  add_column(Q15_7_walk) %>% 
  add_column(Q15_8_meet) %>% 
  add_column(Q15_9_alcohol) %>% 
  add_column(Q15_10_drugs) %>% 
  add_column(Q15_11_tobacco) %>% 
  add_column(Q15_12_other) %>% 
  add_column(Q15_13_nothing) %>% 
  add_column(Q15_14_never_lonely)
```

## Question 15: What would you like to change in your family - write a short paragraph.

This question is not easily captured numerically, so it does not enter the dataset

## Question 16: When we feel lonely, we very often experience strong feelings and thoughts. What is the extent to which you suffer from them.

This question is very subjective and we have doubts about the coding suggested. Therefore it does not enter the dataset.

## Question 17: Have you ever spoke to anyone about your feeling of loneliness.

1. No, even though i sometimes suffer from it
2. Yes (with whom?)

Again, this question is poorly formulated and therefore hard to cast as a numerical variable. For example it does not give the respondents a chance to say “I do not feel lonely”, therefore it is impossible to differentiate between those subjects and real NAs. It does not enter the dataset.

## Question 18: During the last month, did (1-no, never/ 2 - once / 3 - more than once:

1. Your parents shout at you
2. Your parents insult you
3. Your parents hit you on the face
4. Your parents beat you
5. Your parents quarreled among themselves using insults and being aggressive
6. Said you were worth nothing and nothing will become of you
7. You felt really bad at home
8. You fled home because you were afraid you would be beaten
9. You were afraid of your parents
10. You parents denied you food or sleep
11. A member of your family used “bad touch” on you
12. Police came because of a row

This question will be coded as 12 numerical columns

```
bad_things <- list(
  "  nie  " = 1, 
  "  raz  " = 2,
  "  więcej niż raz  " = 3
)
Q18_1_shout <- fill_column(levels_list = bad_things, 
                                template_column = spss_data$z18_a)
Q18_2_insult <- fill_column(levels_list = bad_things, 
                                template_column = spss_data$z18_b)
Q18_3_hit_face <- fill_column(levels_list = bad_things, 
                                template_column = spss_data$z18_d)
Q18_4_beat <- fill_column(levels_list = bad_things, 
                                template_column = spss_data$z18_e)
Q18_5_quarrel <- fill_column(levels_list = bad_things, 
                                template_column = spss_data$z18_f)
Q18_6_worthless <- fill_column(levels_list = bad_things, 
                                template_column = spss_data$z18_g)
Q18_7_felt_bad <- fill_column(levels_list = bad_things, 
                                template_column = spss_data$z18_h)
Q18_8_fled <- fill_column(levels_list = bad_things, 
                                template_column = spss_data$z18_i)
Q18_9_afraid <- fill_column(levels_list = bad_things, 
                                template_column = spss_data$z18_j)
Q18_10_touch <- fill_column(levels_list = bad_things, 
                                template_column = spss_data$z18_k)
Q18_11_police <- fill_column(levels_list = bad_things, 
                                template_column = spss_data$z18_l)

mood_data %<>% 
  add_column(Q18_1_shout) %>% 
  add_column(Q18_2_insult) %>% 
  add_column(Q18_3_hit_face) %>% 
  add_column(Q18_4_beat) %>% 
  add_column(Q18_5_quarrel) %>% 
  add_column(Q18_6_worthless) %>% 
  add_column(Q18_7_felt_bad) %>% 
  add_column(Q18_8_fled) %>% 
  add_column(Q18_9_afraid) %>% 
  add_column(Q18_10_touch) %>% 
  add_column(Q18_11_police)
```

## Question 19: Based on your personal experience, how much influence do you have on (rate on the folowing scale 1 - always, 2 - usually, 3 - sometimes, 4- rarely, 5 - hardly ever, 6 - usually not, 7 - definitely not)

1. Your material status
2. Your health
3. Your grades
4. Your future career
5. Your relationships with others
6. Your relationships with a boyfriend/girlfriend
7. Your appearance
8. Your relationships with your parents
9. Your free time

This question will be coded as a number of numeric columns. The scores will be reversed - the answer ‘always’ will be given 7.

```
control_over_things <- list(
  "  Zawsze  " = 7, 
  "  Zazwyczaj  "  = 6, 
  "  Czasami  " = 5, 
  "  Rzadko  " = 4, 
  "  Prawie wcale  " = 3,
  "  Raczej nie  " = 2,
  "  Zdecydowanie nie  " = 1
)

Q19_1_material <- fill_column(levels_list = control_over_things, 
                                template_column = spss_data$z19_a)
Q19_2_health <- fill_column(levels_list = control_over_things, 
                                template_column = spss_data$z19_b)
Q19_3_grades <- fill_column(levels_list = control_over_things, 
                                template_column = spss_data$z19_c)
Q19_4_career <- fill_column(levels_list = control_over_things, 
                                template_column = spss_data$z19_d)
Q19_5_rel_others <- fill_column(levels_list = control_over_things, 
                                template_column = spss_data$z19_e)
Q19_6_rel_bg <- fill_column(levels_list = control_over_things, 
                                template_column = spss_data$z19_f)
Q19_7_appearance <- fill_column(levels_list = control_over_things, 
                                template_column = spss_data$z19_g)
Q19_8_rel_parents <- fill_column(levels_list = control_over_things, 
                                template_column = spss_data$z19_h)
Q19_9_time <- fill_column(levels_list = control_over_things, 
                                template_column = spss_data$z19_i)

mood_data %<>% 
  add_column(Q19_1_material) %>% 
  add_column(Q19_2_health) %>% 
  add_column(Q19_3_grades) %>% 
  add_column(Q19_4_career) %>% 
  add_column(Q19_5_rel_others) %>% 
  add_column(Q19_6_rel_bg) %>% 
  add_column(Q19_7_appearance) %>% 
  add_column(Q19_8_rel_parents) %>% 
  add_column(Q19_9_time)
```

## Question 20: Think about to what extent the traits listed below are true of you, then rate according to the following scheme: high extent - 1, medium - 2, little - 3, not at all -1.

1. Attractive
2. Intelligent
3. Pro-active
4. Sociable
5. Hard-working
6. Protective
7. Responsible
8. Self-sufficient
9. Forgiving
10. Frugal
11. Determined
12. Frank
13. Confident

All these reflect a person’s assessment of themselves. We don’t believe any one of them will be more important than the others, so we have combined them into a single column, `self_assessment`, which is an average of scores for all the above. Also, the scoring will be reversed.

```
assessment <- list(
  "  bardzo wysokim  " = 4, 
  "  średnim  " = 3, 
  "  średnim  " = 2,
  "  wcale  " = 1
)
Q20_1_attractive <- fill_column(levels_list = assessment, 
                                template_column = spss_data$z20_a)
Q20_2_intelligent <- fill_column(levels_list = assessment, 
                                template_column = spss_data$z20_b)
Q20_3_proactive <- fill_column(levels_list = assessment, 
                                template_column = spss_data$z20_c)
Q20_4_sociable <- fill_column(levels_list = assessment, 
                                template_column = spss_data$z20_d)
Q20_5_hardworking <- fill_column(levels_list = assessment, 
                                template_column = spss_data$z20_e)
Q20_6_protective <- fill_column(levels_list = assessment, 
                                template_column = spss_data$z20_f)
Q20_7_responsible <- fill_column(levels_list = assessment, 
                                template_column = spss_data$z20_g)
Q20_8_self_sufficient <- fill_column(levels_list = assessment, 
                                template_column = spss_data$z20_h)
Q20_9_forgiving <- fill_column(levels_list = assessment, 
                                template_column = spss_data$z20_i)
Q20_10_frugal <- fill_column(levels_list = assessment, 
                                template_column = spss_data$z20_j)
Q20_11_proactive <- fill_column(levels_list = assessment, 
                                template_column = spss_data$z20_k)
Q20_12_frank <- fill_column(levels_list = assessment, 
                                template_column = spss_data$z20_l)
Q20_13_confident <- fill_column(levels_list = assessment, 
                                template_column = spss_data$z20_m)
Q20_self_assessment <- rowMeans(data.frame(Q20_1_attractive,
                                        Q20_2_intelligent,
                                        Q20_3_proactive, 
                                        Q20_4_sociable,
                                        Q20_5_hardworking,
                                        Q20_6_protective,
                                        Q20_7_responsible,
                                        Q20_8_self_sufficient,
                                        Q20_9_forgiving,
                                        Q20_10_frugal,
                                        Q20_11_proactive,
                                        Q20_12_frank,
                                        Q20_13_confident), na.rm = TRUE)

Q20_self_assessment[is.nan(Q20_self_assessment)] <- NA
mood_data %<>% 
  add_column(Q20_self_assessment)
```

## Question 21: Circle the answer on the scale of 0-4 that best characterizes your well-being during the recent week, up until today. (0 - not at all, 1 - rarely, 2 - sometimes, 3 - often, 4 - very often)

1. Thoughts and feelings

1. feeling of sadness or depression
2. worse mood than usual
3. crying
4. discouragement
5. hopelessness
6. low self-esteem
7. feeling worthless and inadequate
8. feeling of guilt and shame
9. blaming and criticizing yourself
10. difficulty making decisions

2. Relationships with other people and acting in the world

1. loss of interest in family life, friends and acquaintances
2. loneliness
3. spending less time with family or friends
4. loss of motivation
5. loss of interest in school
6. avoiding school (work) and other activity
7. loss of joy of life

3. Physiological manifestations

1. felling of being overworked
2. trouble falling asleep or sleeping too long
3. loss of appetite
4. worrying about your health

4. Suicidal impulses

1. do you have suicidal thoughts?
2. would you like to end your life?
3. do you plan to harm yourself?

Each of these groups of questions will be grouped as one column, represented by the mean of the answers accross the group.

```
well_being <- list(
  "  wcale  " = 0, 
  "  rzadko  " = 1, 
  "  czasami  " = 2,
  "  często  " = 3,
  "  bardzo często  " = 4
  
)

attach(spss_data)
Q21_thoughts_and_feelings <- fill_and_collect_columns(list(z21_m1, z21_m2, z21_m3, z21_m4, z21_m5, z21_m6, z21_m7, z21_m8, z21_m9, z21_m10), well_being) %>% 
  rowMeans(na.rm = TRUE)
Q21_thoughts_and_feelings[is.nan(Q21_thoughts_and_feelings)] <- NA
Q21_relationships_people <- fill_and_collect_columns(list(z21_z1, z21_z2, z21_z3, z21_z4, z21_z5, z21_z6, z21_z7), well_being) %>% 
  rowMeans(na.rm = TRUE)
Q21_relationships_people[is.nan(Q21_relationships_people)] <- NA
Q21_phys <- fill_and_collect_columns(list(z21_o1, z21_o2, z21_o3, z21_o4), well_being) %>% 
  rowMeans(na.rm = TRUE)
Q21_phys[is.nan(Q21_phys)] <- NA
Q21_suic <- fill_and_collect_columns(list(z21_i1, z21_i2, z21_i3), well_being) %>% 
  rowMeans(na.rm = TRUE)
Q21_suic[is.nan(Q21_suic)] <- NA
detach(spss_data)

mood_data %<>%
  add_column(Q21_thoughts_and_feelings) %>% 
  add_column(Q21_relationships_people) %>% 
  add_column(Q21_phys) %>% 
  add_column(Q21_suic)
```

## Question 22: what kind of student are you?

1. Outstanding (average grade above 5.0)
2. Good or very good (average between 4.0 and 4.9)
3. Average (average between 3.0 and 3.9)
4. Moderate (average below 3.0)

```
grades <- list(
  "  Bardzo dobrym  " = 6,
  "  Dobrym  " = 5, 
  "  Dostatecznym  " = 4, 
  "  Miernym  " =  3
)

Q22_school_achievement <- fill_column(grades, spss_data$z22)
mood_data %<>%
  add_column(Q22_school_achievement)
```

## Question 23: have you ever repeated a grade? If, yes, which one?

1. Yes
2. No

Only yes and no answers will be retained - the information on which grade was repeated is dropped (in fact, in the original spss data file there was no information on which grade it was).

```
Q23_repeated_grade <- yes_no_relevel(spss_data$z23)
mood_data %<>%
  add_column(Q23_repeated_grade)
```

## Question 24: which of the following are the most difficult one at school? Choose at most 3.

1. Getting a positive grade for each subject
2. Getting a positive grade for some subjects
3. Building good relationships with peers
4. Building good relationships with teachers
5. Behaving well during classes
6. Behaving well during breaks and outside school
7. None of the above are difficult for me
8. Other

This question is fine-grained - the answers will be coded as having problems at school vs not having problems at school

```
helper_df <- data.frame(spss_data$z24_1, spss_data$z24_2, spss_data$z24_3)
Q24_problems_at_shool <- rep(NA, length(spss_data$z24_1))
for (idx in seq_along(spss_data$z24_1)) {
  if (!all(is.na(helper_df[idx, ])) && any(helper_df[idx, ] == "  Nic mi nie sprawia trudności  ", na.rm = TRUE)) {
    Q24_problems_at_shool[idx] <- 0
  } else {
    if (any(!is.na(helper_df[idx, ]))) {
      Q24_problems_at_shool[idx] <- 1
    }
  }
}

mood_data %<>% 
  add_column(Q24_problems_at_shool)
```

## Question 25: Do you feel safe at school?

1. Yes, definitely (4)
2. Yes, I think I am (3)
3. I am not sure I am (2)
4. No (1)

This question has a natural ordering so it will be coded as a numerical variable - the values assigned given in the brackets.

```
safety <- list(
  "  zdecydowanie tak  " = 4, 
  "  raczej tak  " = 3, 
  "  raczej nie  " = 2, 
  "  zdecydowanie nie  " = 1
)

Q25_safe_at_school <- fill_column(safety, spss_data$z25)
mood_data %<>%
  add_column(Q25_safe_at_school)
```

## Question 26: How do you rate your realtionships with other students in you group?

1. Very good (4)
2. Good enough (3)
3. Quite bad (2)
4. Bad (1)

This question has a natural ordering so it will be coded as a numerical variable - the values assigned given in the brackets.

```
school_relationships <- list(
  "Bardzo dobre" = 4, 
  "Raczej dobre" = 3, 
  "Raczej złe" = 2, 
  "Zdecydowanie złe" = 1
)
Q26_school_relationships <- fill_column(school_relationships, spss_data$z26)
mood_data %<>%
  add_column(Q26_school_relationships)
```

## Question 27: How do you feel comparing yourself to your peers in the following categories? (Rate using the following scale: 0 - I always feel inferior, 1 - I sometimes feel inferior, 2 - I afeel equal to others, 3 - I sometimes feel superior, 4 - I always feel superior).

1. Clothes and gadgets
2. Looks (height, weight etc.)
3. School marks
4. Popularity with the opposite sex
5. Physical fitness
6. Aptitude and skill
7. Health (frequent illnesses)
8. Specific handicaps (stuttering, dislexia, hyperactivity)
9. Financial situation
10. Possibility to go out (cinema, dico)
11. Religion, denomination
12. Nationality
13. Ability to go abroad on holiday
14. Good cosmetics
15. Living situation
16. Financial situation of your family
17. Another family family circumstances
18. Other

This question is very detailed and introduces many topics, but all of them are related to comparing oneself to others. Therefore, answers across all sub-questions will be averaged.

```
attach(spss_data)
comparisons <- list(
                    "  Zawsze czuję się gorszy  " = 0,
                    "  Czasem czuję się gorszy  " = 1,
                    "  Jestem taki sam jak inni  " = 2,
                    "  Czasem czuje się lepszy  " = 3,
                    "  Zawsze czuję się lepszy  " = 4,
                    "  brak danych  " = NA
)
Q27_comparisons <- fill_and_collect_columns(list(z27_a, z27_b, z27_c, z27_d, z27_e, z27_f,  z27_g, z27_h, z27_i, z27_j, z27_k, z27_l, z27_m, z27_n, z27_o, z27_p, z27_r, z27_s), comparisons) %>% 
  rowMeans(na.rm = TRUE)
detach(spss_data)

Q27_comparisons[is.nan(Q27_comparisons)] <- NA
mood_data %<>% 
  add_column(Q27_comparisons)
```

## Question 28: Are you part of a peer group?

1. yes
2. no

```
Q28_peer_group <- yes_no_relevel(spss_data$z28)
mood_data %<>% 
  add_column(Q28_peer_group)
```

## Question 29: Do you sometimes feel lonely in your peer group?

1. Yes, very often (3)
2. Yes, sometimes (2)
3. Yes, but rarely (1)
4. I am never lonely in my peer group (0)

This question has a clear numerical representation - the score has been given next to each question.

```
loneliness_in_group <- list(
  "  Tak, bardzo często  " = 3,
  "  Tak, czasami je odczuwam  " = 2,
  "Tak, ale bardzo rzadko  " = 1,
"  Nigdy nie odczuwam osamotnienia w relacjach z kolegami  " = 0
)


Q29_lonely_in_peer_group <- fill_column(loneliness_in_group, spss_data$z29)
mood_data %<>% 
  add_column(Q29_lonely_in_peer_group)
```

## Question 30: How often do you do the following? (Rate on the scale 1 - a few times a week, 2 - a few times in a semester, 3 - 2-3 times in a school year, 4 - never)

1. skip school
2. run away from home
3. smoke
4. petty theft
5. steal (something that would be reported to the police)
6. beat somebody from school
7. beat somebody outside of school
8. take drugs
9. harm yourself
10. use alcohol
11. use drugs
12. use powerups (kind of drug, but not regulated)
13. call your friends names
14. extort (money, cigarettes)
15. destroy somebody else’s property
16. destroy school property
17. destroy social property
18. make graffiti
19. record somebody with your phone with the intention of making fun of this person
20. bully somebody

This question is very detailed and introduces many topics, but all of them are related to comparing oneself to others. Therefore, answers across all questions will be averaged. The scale will be reversed to reflect the gravity of the social behavior.

```
attach(spss_data)
antisocial <- list(
  "  Kilka razy w tygodniu  "  = 3,
  "  Kilka razy w  semestrze  " = 2,
  "  2 -3 razy w roku szkolnym  " = 1, 
  "  Nie zdarzyło mi się  " = 0
          
)
Q30_antisocial_behavior <- fill_and_collect_columns(list(z30_a, z30_b, z30_c, z30_d, z30_e, z30_f, z30_g, z30_h, z30_i, z30_j, z30_k, z30_l, z30_m, z30_n, z30_o, z30_p, z30_q, z30_r, z30_s, z30_t), antisocial) %>% 
  rowMeans(na.rm = TRUE)
detach(spss_data)
Q30_antisocial_behavior[is.nan(Q30_antisocial_behavior)] <- NA
mood_data %<>% 
  add_column(Q30_antisocial_behavior)
```

## Question 31: Which of the behaviors above happen to you?

Since the antisocial behavior was averaged, this question will not be taken into account.

## Question 32: Have you ever been (rank on the scale: 1- a few times a week, 2, a few times during the semester, 3 - 1-3 times during a school year, 4 - never happened to me):

1. robbed
2. beaten by peers at school
3. beaten by peers outside school
4. forced to taken drugs
5. forced to do something you did not want to do
6. called names
7. injured
8. filmed with the intention to be ridiculed
9. forced to drink alcohol
10. drugged
11. extorted
12. a victim of property damage

This question is very detailed and introduces many topics, but all of them are related to being a victim. Therefore, answers across all questions will be averaged. The scale will be reversed to reflect the gravity of being a victim.

```
attach(spss_data)
victim <- list(
  "  Kilka razy w tygodniu  "  = 3,
  "  Kilka razy w  semestrze  " = 2,
  "  1-3 razy w roku szkolnym  " = 1, 
  "  Nie zdarzyło mi się  " = 0
          
)
Q32_victim <- fill_and_collect_columns(list(spss_data$z32_1, spss_data$z32_2, spss_data$z32_3, spss_data$z32_4, spss_data$z32_5, spss_data$z32_6, spss_data$z32_7, spss_data$z32_8, spss_data$z32_9, spss_data$z32_10, spss_data$z32_11,spss_data$z32_12), victim) %>% 
  rowMeans(na.rm = TRUE)
detach(spss_data)
Q32_victim[is.nan(Q32_victim)] <- NA
mood_data %<>% 
  add_column(Q32_victim)
```

## Question 33: Who in your opinion is most at risk of violence?

This question reflects the opinion of the respondent and has the character of an open-ended question. It will not be used in the study

## Question 34. Is there somebody who you can ask for help?

1. Yes
2. Usually
3. Usually not
4. No

This question has a natural ordering.

```
support <- list(
  "  tak  " = 3, 
  "  raczej tak  " = 2, 
  "  raczej nie  " = 1,
  "  nie  "  = 0
)
Q34_support <- fill_column(levels_list = support, 
                                template_column = spss_data$z34)
mood_data %<>% 
  add_column(Q34_support)
```

## Question 35: If you answered yes to the previous question, write who it is

This question will not be used in the study

## Question 36: Is there somebody who you can ask for advice?

```
support_advice <- list(
  "  tak  " = 3, 
  "  raczej tak  " = 2, 
  "  raczej nie  " = 1,
  "  nie  "  = 0
)
Q36_support_advice <- fill_column(levels_list = support_advice, 
                                template_column = spss_data$z36)
mood_data %<>% 
  add_column(Q36_support_advice)
```

## Question 37: If you answered yes to the previous question, write who it is

This question will not be used in the study

## Question 38: Do you have a friend?

1. yes
2. no

```
Q38_friend <- yes_no_relevel(spss_data$z38)
mood_data %<>% 
  add_column(Q38_friend)
```

## Question 39: In what matters do you ask the teacher for help?

This question will not be used in the study

## Question 40: In what matters do you ask the school psychologist for help?

This question will not be used in the study

## Question 41: Who is an authority to you?

This question gives the respondent a list of potential persons of authority and also lets them give their own answer. We se no way to use it numerically.

## Question 42: Do you participate in extracurricular activities at school?

1. Yes
2. No Say which (this information will be dropped)

```
Q42_extracurricular_1 <- yes_no_relevel(spss_data$z42_1)
Q42_extracurricular_2 <- yes_no_relevel(spss_data$z42_2)
Q42_extracurricular_3 <- yes_no_relevel(spss_data$z42_4)
Q42_extracurricular_4 <- yes_no_relevel(spss_data$z42_4)
Q42_extracurricular_5 <- yes_no_relevel(spss_data$z42_5)
Q42_extracurricular_6 <- yes_no_relevel(spss_data$z42_6)
extracurriculars <- data.frame(Q42_extracurricular_1, Q42_extracurricular_2, Q42_extracurricular_3, Q42_extracurricular_4, Q42_extracurricular_5, Q42_extracurricular_6)

Q42_extracurricular <- apply(extracurriculars, 1, function(row) {
  if (all(is.na(row))) {
    NA
  } else {
    "yes" %in% row %>% 
      as.numeric()
  }
})

mood_data %<>% 
  add_column(Q42_extracurricular)
```

## Question 43: Do you participate in extracurricular activities outside of school?

1. Yes
2. No Say which (this information will be dropped)

```
Q43_extracurricular_outside1 <- yes_no_relevel(spss_data$z43_1)
Q43_extracurricular_outside2 <- yes_no_relevel(spss_data$z43_2)
Q43_extracurricular_outside3 <- yes_no_relevel(spss_data$z43_4)
Q43_extracurricular_outside4 <- yes_no_relevel(spss_data$z43_4)
Q43_extracurricular_outside5 <- yes_no_relevel(spss_data$z43_5)
extracurriculars_outside <- data.frame(Q43_extracurricular_outside1, Q43_extracurricular_outside2, Q43_extracurricular_outside3, Q43_extracurricular_outside4, Q43_extracurricular_outside5)

Q43_extracurricular_outside <- apply(extracurriculars_outside, 1, function(row) {
  if (all(is.na(row))) {
    NA
  } else {
    "yes" %in% row %>% 
      as.numeric()
  }
})
mood_data %<>% 
  add_column(Q43_extracurricular_outside)
```

## 44. Do you volunteer?

1. Yes
2. No Say which (this information will be dropped)

```
Q44_volunteer_1 <- yes_no_relevel(spss_data$z44_1)
Q44_volunteer_2 <- yes_no_relevel(spss_data$z44_2)
Q44_volunteer_3 <- yes_no_relevel(spss_data$z44_4)
Q44_volunteer_4 <- yes_no_relevel(spss_data$z44_4)
Q44_volunteer_5 <- yes_no_relevel(spss_data$z44_5)
volunteer <- data.frame(Q44_volunteer_1, Q44_volunteer_2, Q44_volunteer_3, Q44_volunteer_4, Q44_volunteer_5)

Q44_volunteer <- apply(volunteer, 1, function(row) {
  if (all(is.na(row))) {
    NA
  } else {
    "yes" %in% row %>% 
      as.numeric()
  }
})

mood_data %<>% 
  add_column(Q44_volunteer)
```

## Question 45: How do you spend your free time in your peer group?

There is a list of choices about this for the respondent to select. We believe this question is very imprecise and for this reason it will not be taken into account.

## Question 46: Which values are the most important to your peer group? Answer yes/no.

1. Education
2. Work
3. Health
4. Money
5. Religion
6. Play,
7. Being active in the society
8. Other?

```
Q46_1_education <- yes_no_relevel(spss_data$z46_1)
Q46_2_work <- yes_no_relevel(spss_data$z46_2)
Q46_3_health <- yes_no_relevel(spss_data$z46_3)
Q46_4_money <- yes_no_relevel(spss_data$z46_4)
Q46_5_religion <- yes_no_relevel(spss_data$z46_5)
Q46_6_play <- yes_no_relevel(spss_data$z46_6)
Q46_7_active_in_soc <- yes_no_relevel(spss_data$z46_7)
Q46_8_other <- yes_no_relevel(spss_data$z46_8)

mood_data %<>%
  add_column(Q46_1_education) %>% 
  add_column(Q46_2_work) %>% 
  add_column(Q46_3_health) %>% 
  add_column(Q46_4_money) %>% 
  add_column(Q46_5_religion) %>% 
  add_column(Q46_6_play) %>% 
  add_column(Q46_7_active_in_soc) %>% 
  add_column(Q46_8_other)
```

## Question 47: Do you have a hobby?

1. Yes
2. No

```
Q47_hobby <- yes_no_relevel(spss_data$z47)
mood_data %<>%
  add_column(Q47_hobby)
```

## Question 48: Sometimes you don’t spend time in any of the ways described in the previous questions. How do you cope with boredom?

This is an open-ended question - will not be used in the study.

## Question 49: How often do you do the following (rank on the scale 1- at least once a week, 2- at least once a month, 3 - a few times a year, 4 - once a year or more rarely, 5 - I have never done this)

1. go to the cinema
2. go to the theater
3. go to the philharmonic
4. go to an exhibition
5. go to a museum

This is very detailed, so it will be averaged across all institutions of culture. The scale order will be reversed.

```
attach(spss_data)
culture <- list(
  "  Przynajmniej raz w tygodniu  "  = 4,
  "  Przynajmniej raz w miesiącu  " = 3,
  "  Kilka razy w roku  " = 2,
  "  Rzadziej  " = 1, 
  "  Nigdy nie byłem  " = 0
          
)

Q49_culture <- fill_and_collect_columns(list(z49_1, z49_2, z49_3, z49_4, z49_5), culture) %>% 
  rowMeans(na.rm = TRUE)
detach(spss_data)

Q49_culture[is.nan(Q49_culture)] <- NA

mood_data %<>% 
  add_column(Q49_culture)
```

## Question 50: Do you have access to:

cell phone, computer, internet, gaming console, TV

This question is practically repeated in the following questions, so it will not be included

## Question 51: Think about how much time you devote to each of the activities listed above 1) during week days 2) during weekends. (rate on the following scale 1 - not at all, 2 - under 2 hours, 3 - 2-4 hours, 4 - above 4 hours)

1. talking on the phone
2. sms
3. computer games
4. online communicators
5. running your own blog
6. looking for new friends
7. watching films on the internet
8. reading political news
9. watching TV
10. console games

The answers will be averaged over weekdays and weekends. Console games will be combined with computer games. We are aware that question 6 is very vague.

```
time <- list(
  "  W ogóle nie korzystam  " = 1, 
  "  Poniżej 2 godzin  " = 2, 
  "  Od 2 do 4 godz  " = 3, 
  "  Powyżej 4 godz  " = 4
)

attach(spss_data)
Q51_1_phone <- fill_and_collect_columns(list(z51_1p, z51_1w), time) %>% 
  rowMeans(na.rm = TRUE)
Q51_1_phone[is.nan(Q51_1_phone)] <- NA
Q51_2_sms <- fill_and_collect_columns(list(z51_2p, z51_2w), time) %>% 
  rowMeans(na.rm = TRUE)
Q51_2_sms[is.nan(Q51_2_sms)] <- NA
Q51_3_c_games <- fill_and_collect_columns(list(z51_3p, z51_10p, z51_3w, z51_10w), time) %>% 
  rowMeans(na.rm = TRUE)
Q51_3_c_games[is.nan(Q51_3_c_games)] <- NA
Q51_4_communicators <- fill_and_collect_columns(list(z51_4p, z51_4w), time) %>% 
  rowMeans(na.rm = TRUE)
Q51_4_communicators[is.nan(Q51_4_communicators)] <- NA
Q51_5_blog <- fill_and_collect_columns(list(z51_5p, z51_5w), time) %>% 
  rowMeans(na.rm = TRUE)
Q51_5_blog[is.nan(Q51_5_blog)] <- NA
Q51_6_friends <- fill_and_collect_columns(list(z51_6p, z51_6w), time) %>% 
  rowMeans(na.rm = TRUE)
Q51_6_friends[is.nan(Q51_6_friends)] <- NA
Q51_7_i_films <- fill_and_collect_columns(list(z51_7p, z51_7w), time) %>% 
  rowMeans(na.rm = TRUE)
Q51_7_i_films[is.nan(Q51_7_i_films)] <- NA
Q51_8_news <- fill_and_collect_columns(list(z51_8p, z51_8w), time) %>% 
  rowMeans(na.rm = TRUE)
Q51_8_news[is.nan(Q51_8_news)] <- NA
Q51_9_TV <- fill_and_collect_columns(list(z51_9p, z51_9w), time) %>% 
  rowMeans(na.rm = TRUE)
Q51_9_TV[is.nan(Q51_9_TV)] <- NA
detach(spss_data)

mood_data %<>% 
  add_column(Q51_1_phone) %>% 
  add_column(Q51_2_sms) %>% 
  add_column(Q51_3_c_games) %>% 
  add_column(Q51_4_communicators) %>% 
  add_column(Q51_5_blog) %>% 
  add_column(Q51_6_friends) %>% 
  add_column(Q51_7_i_films) %>% 
  add_column(Q51_8_news) %>% 
  add_column(Q51_9_TV)
```

## Question 52: How often do your parents complain that you devote too much time to the following: (rate on the scale 1 - never, 2 - a few times a month, 3 - a few times a week, 4 - every day)

1. talking on the cell phone
2. writing and sending sms
3. sitting in front of a computer
4. using the internet
5. console games
6. watching TV

All of the above involve parents complaining about wasting time on technology related distractions by the respondents. Therefore, all will be averaged.

```
wasting_time <- list(
  "  Nigdy  " = 1, 
  "  Kilka razy w miesiącu  " = 2, 
  "  Kilka razy w tygodniu  " = 3,
  "  Codziennie  " = 4
)
Q_52_wasting_time <- fill_and_collect_columns(list(spss_data$z52_1, 
                                                   spss_data$z52_2, 
                                                   spss_data$z52_3, 
                                                   spss_data$z52_4, 
                                                   spss_data$z52_5, 
                                                   spss_data$z52_6), wasting_time) %>% rowMeans(na.rm = TRUE)
Q_52_wasting_time[is.nan(Q_52_wasting_time)] <- NA
mood_data %<>% 
  add_column(Q_52_wasting_time)
```

## Question 53: Does it happen that you put off school work or your chores to have more time to do the following: (rate on the following scale: 1 - never, 2 - a few times a month, 3 - a few times a week, 4 - every day)

1. talking on the cell phone
2. writing and sending sms
3. sitting in front of a computer
4. using the internet
5. console games
6. watching TV

All of the above involve procrastination, therefore, all will be averaged.

```
wasting_time <- list(
  "  Nigdy  " = 1, 
  "  Kilka razy w miesiącu  " = 2, 
  "  Kilka razy w tygodniu  " = 3,
  "  Codziennie  " = 4
)
Q_53_procrastination <- fill_and_collect_columns(list(spss_data$z53_1, 
                                                   spss_data$z53_2, 
                                                   spss_data$z53_3, 
                                                   spss_data$z53_4, 
                                                   spss_data$z53_5, 
                                                   spss_data$z53_6), wasting_time) %>% rowMeans(na.rm = TRUE)
Q_53_procrastination[is.nan(Q_53_procrastination)] <- NA
mood_data %<>% 
  add_column(Q_53_procrastination)
```

## Question 54: gender:

1. male
2. female

Only these two options were given in the questionnaire.

```
gender <- list(
  "  chłopcem  " = 0, 
  "  dziewczyną  " = 1
)

Q54_gender <- fill_column(levels_list = gender, template_column = spss_data$z54m)

mood_data %<>% 
  add_column(Q54_gender)
```

## Question 55: What is the education level of your parents (mother and father separately):

1. primary education (1)
2. trade school (2)
3. secondary education (3)
4. higher education (4)
5. I don’t know (NA)

This question has a clear numerical structure. The numeric value of each choice is given in brackets.

```
education <- list(
  "  Podstawowe  " = 1, 
  "  Zawodowe  " = 2, 
  "  Średnie  " = 3,
  "  Wyższe  " = 4, 
  "  nie wiem  " = NA
)

Q55_mother <- fill_column(levels_list = education, template_column = spss_data$z55m_1)
Q55_father <- fill_column(levels_list = education, template_column = spss_data$z55m_2)

mood_data %<>%
  add_column(Q55_mother) %>% 
  add_column(Q55_father)
```

## Question 56: How many children there are in your family, including you?

1. one
2. two
3. three or more

This question has a clear numerical structure.

```
children <- list(
  "  jedno  " = 1,
  "  dwoje  " = 2, 
  "  troje i więcej  " = 3
)

Q56_children <- fill_column(levels_list = children, template_column = spss_data$z56)
```

## NAs

```
apply(mood_data, 2, function(column) sum(is.na(column)))
```

```
##                          mood           Q1_further_learning 
##                           221                             2 
##           Q2_school_plan_real            Q3_edu_fear_1_exam 
##                             7                            34 
##         Q3_edu_fear_2_consent           Q3_edu_fear_3_money 
##                            69                            89 
##            Q3_edu_fear_4_work            Q3_edu_fear_5_none 
##                            83                           115 
##           Q3_edu_fear_6_other             Q5_raised_by_both 
##                           831                            10 
##           Q5_raised_by_mother           Q5_raised_by_father 
##                            10                            10 
##            Q5_raised_by_other      Q5_raised_outside_family 
##                            10                            10 
##  Q6_mother_works_domestically        Q6_mother_works_abroad 
##                            31                            31 
##       Q6_mother_does_not_work  Q6_father_works_domestically 
##                            31                           115 
##        Q6_father_works_abroad       Q6_father_does_not_work 
##                           115                           115 
##        Q7_financial_situation   Q8_family_concern_1_unemp_2 
##                            40                            49 
##   Q8_family_concern_2_unemp_2    Q8_family_concern_3_income 
##                            54                            48 
## Q8_family_concern_4_conflicts   Q8_family_concern_5_alcohol 
##                            51                            59 
##      Q8_family_concern_6_debt     Q8_family_concern_8_money 
##                            52                            54 
## Q8_family_concern_9_long_work     Q8_family_concern_10_time 
##                            46                            55 
## Q8_family_concern_11_handicap       Q8_family_concern_12_no 
##                            58                            76 
##                Q9_help_1_save                Q9_help_2_home 
##                            71                            81 
##             Q9_help_3_at_work                Q9_help_4_work 
##                            90                            81 
##         Q9_help_5_no_problems               Q9_help_6_other 
##                           106                           702 
##                      Q10_work           Q11_work_by_parents 
##                             0                          2257 
##             Q11_work_I_should        Q11_work_own_decission 
##                          2257                          2257 
##              Q11_work_friends               Q11_work_others 
##                          2257                          2257 
##     Q12_give_money_to_parents       Q12_keep_some_give_some 
##                          2298                          2298 
##            Q12_support_myself                 Q12_pleasures 
##                          2298                          2298 
##          Q12_peasures_support                   Q13_1_happy 
##                          2298                            17 
##              Q13_2_not_notice          Q13_3_not_interested 
##                            15                            19 
##               Q13_4_can_count                 Q13_5_if_love 
##                            14                            18 
##                    Q13_6_time                   Q13_7_close 
##                            25                            21 
##          Q13_8_difficult_talk               Q13_9_important 
##                            32                            20 
##              Q13_10_important                   Q14_1_music 
##                            19                             9 
##                      Q14_2_TV          Q14_3_computer_games 
##                             9                             9 
##                  Q14_4_surf_I                    Q15_5_talk 
##                             9                             9 
##                    Q15_6_talk                    Q15_7_walk 
##                             9                             9 
##                    Q15_8_meet                 Q15_9_alcohol 
##                             9                             9 
##                  Q15_10_drugs                Q15_11_tobacco 
##                             9                             9 
##                  Q15_12_other                Q15_13_nothing 
##                             9                             9 
##           Q15_14_never_lonely                   Q18_1_shout 
##                             9                            11 
##                  Q18_2_insult                Q18_3_hit_face 
##                            14                            10 
##                    Q18_4_beat                 Q18_5_quarrel 
##                            12                            16 
##               Q18_6_worthless                Q18_7_felt_bad 
##                            19                             9 
##                    Q18_8_fled                  Q18_9_afraid 
##                            11                            12 
##                  Q18_10_touch                 Q18_11_police 
##                             8                            11 
##                Q19_1_material                  Q19_2_health 
##                            48                            29 
##                  Q19_3_grades                  Q19_4_career 
##                            37                            23 
##              Q19_5_rel_others                  Q19_6_rel_bg 
##                            48                            33 
##              Q19_7_appearance             Q19_8_rel_parents 
##                            22                            28 
##                    Q19_9_time           Q20_self_assessment 
##                            21                             9 
##     Q21_thoughts_and_feelings      Q21_relationships_people 
##                             1                             3 
##                      Q21_phys                      Q21_suic 
##                             4                             4 
##        Q22_school_achievement            Q23_repeated_grade 
##                            10                            11 
##         Q24_problems_at_shool            Q25_safe_at_school 
##                            19                             8 
##      Q26_school_relationships               Q27_comparisons 
##                            13                             2 
##                Q28_peer_group      Q29_lonely_in_peer_group 
##                            29                            34 
##       Q30_antisocial_behavior                    Q32_victim 
##                             6                            13 
##                   Q34_support            Q36_support_advice 
##                            18                           129 
##                    Q38_friend           Q42_extracurricular 
##                            87                            24 
##   Q43_extracurricular_outside                 Q44_volunteer 
##                            47                           117 
##               Q46_1_education                    Q46_2_work 
##                            73                            81 
##                  Q46_3_health                   Q46_4_money 
##                            69                            77 
##                Q46_5_religion                    Q46_6_play 
##                            87                            40 
##           Q46_7_active_in_soc                   Q46_8_other 
##                            92                           885 
##                     Q47_hobby                   Q49_culture 
##                            27                             9 
##                   Q51_1_phone                     Q51_2_sms 
##                            83                           165 
##                 Q51_3_c_games           Q51_4_communicators 
##                            37                           142 
##                    Q51_5_blog                 Q51_6_friends 
##                            74                            72 
##                 Q51_7_i_films                    Q51_8_news 
##                           105                            66 
##                      Q51_9_TV             Q_52_wasting_time 
##                           135                            19 
##          Q_53_procrastination                    Q54_gender 
##                            20                            16 
##                    Q55_mother                    Q55_father 
##                           327                           262
```

Question Q11 contains many NAs - it is present in the raw data rather than being introduced by the data transformation. Therefore, this question is removed from the dataset.

Question Q21 is the basis for the `mood_data` column - it is actually the Burns test which is used to establish mood disorders. Therefore, this question must be removed.

```
mood_data %<>% 
  select(-contains('Q11')) %>% 
  select(-contains('Q21'))
write_rds(mood_data, file = "mood_data.RDS")
```
