# Supplementary material for: Mood Disorder Detection in Adolescents by Classification Trees, Random Forests and XGBoost in Presence of Missing Data
Source: Entropy (Basel). 2021 Sep 14;23(9):1210. doi: 10.3390/e23091210 (PMC8468933; doi:10.3390/e23091210)
Supplement: Supplementary file 1 [file entropy-23-01210-s001.zip › social_AI/xgboost.html]

R Notebook


# R Notebook

# XGBoost

```
library(mlr)
```

```
## Loading required package: ParamHelpers
```

```
## Warning message: 'mlr' is in 'maintenance-only' mode since July 2019.
## Future development will only happen in 'mlr3'
## (<https://mlr3.mlr-org.com>). Due to the focus on 'mlr3' there might be
## uncaught bugs meanwhile in {mlr} - please consider switching.
```

```
library(magrittr)
library(tidyverse)
```

```
## ── Attaching packages ─────────────────────────────────────── tidyverse 1.3.0 ──
```

```
## ✔ ggplot2 3.3.3     ✔ purrr   0.3.4
## ✔ tibble  3.1.0     ✔ dplyr   1.0.5
## ✔ tidyr   1.1.3     ✔ stringr 1.4.0
## ✔ readr   1.4.0     ✔ forcats 0.5.1
```

```
## ── Conflicts ────────────────────────────────────────── tidyverse_conflicts() ──
## ✖ tidyr::extract()   masks magrittr::extract()
## ✖ dplyr::filter()    masks stats::filter()
## ✖ dplyr::lag()       masks stats::lag()
## ✖ purrr::set_names() masks magrittr::set_names()
```

```
library(randomForest)
```

```
## randomForest 4.6-14
```

```
## Type rfNews() to see new features/changes/bug fixes.
```

```
## 
## Attaching package: 'randomForest'
```

```
## The following object is masked from 'package:dplyr':
## 
##     combine
```

```
## The following object is masked from 'package:ggplot2':
## 
##     margin
```

```
library(xgboost)
```

```
## 
## Attaching package: 'xgboost'
```

```
## The following object is masked from 'package:dplyr':
## 
##     slice
```

```
#' Function imputing data with the use of random forest
#' @param data_frame data frame with both predictors and independent data
impute_NAs <- function(data_frame, ntree = 500) {
  transformed_data <- rfImpute(data.matrix(data_frame[, -1]), as.factor(data_frame[["severity"]]), iter=5, ntree=ntree)
  transformed_data[, 1] <- transformed_data[, 1]-1
  transformed_data %<>% 
    as.data.frame()
  names(transformed_data)[[1]] <- 'severity'
  transformed_data$severity <- as.factor(transformed_data$severity)
  transformed_data
}
```

## Data

```
mood_data_train_class <- readRDS("mood_train.RDS")
#mood_data_test_class <- readRDS("mood_test.RDS")
```

imputing missing values

```
mood_data_train_class_matrix <- data.matrix(mood_data_train_class) %>% 
  na.roughfix() %>% 
  as.data.frame()
mood_data_train_class_xg <- impute_NAs(mood_data_train_class)
```

```
## ntree      OOB      1      2
##   500:   6.36%  0.27% 91.59%
## ntree      OOB      1      2
##   500:   6.55%  0.27% 94.39%
## ntree      OOB      1      2
##   500:   6.48%  0.40% 91.59%
## ntree      OOB      1      2
##   500:   6.73%  0.53% 93.46%
## ntree      OOB      1      2
##   500:   6.36%  0.27% 91.59%
```

```
# mood_data_train_class_xg <- mutate_at(mood_data_train_class_matrix, .vars = vars(-severity), .funs = as.numeric)
# mood_data_train_class_xg$severity <- as.factor(mood_data_train_class_xg$severity)
```

## XGBoost: Make a learner and task

```
xgb <- makeLearner("classif.xgboost")
xgb_task <- makeClassifTask(data = mood_data_train_class_xg, target = "severity")
```

## Tuning XGBoost hyperparameters

```
xgb_param_space <- makeParamSet(
  makeNumericParam("eta", lower = 0, upper = 1),
  makeNumericParam("gamma", lower = 0, upper = 5),
  makeIntegerParam("max_depth", lower = 1, upper = 5),
  makeNumericParam("min_child_weight", lower = 1, upper = 10),
  makeNumericParam("subsample", lower = 0.5, upper = 1),
  makeNumericParam("colsample_bytree", lower = 0.5, upper = 1),
  makeIntegerParam("nrounds", lower = 20, upper = 20)#,
  #makeDiscreteParam("eval_metric", values = c("merror", "mlogloss")) # this can't be here because response is binomial!!!
)

rand_search <- makeTuneControlRandom(maxit = 1000)
cv_for_tuning <- makeResampleDesc("CV", iters = 5)

library(parallel)
library(parallelMap)
print(paste("Detected ", detectCores(), " cores", collapse = ""))
```

```
## [1] "Detected  16  cores"
```

```
parallelStartSocket(cpus = detectCores())
```

```
## Starting parallelization in mode=socket with cpus=16.
```

```
tuned_xgb_pars <- tuneParams(xgb, task = xgb_task,
                           resampling = cv_for_tuning,
                           par.set = xgb_param_space,
                           control = rand_search)
```

```
## [Tune] Started tuning learner classif.xgboost for parameter set:
```

```
##                     Type len Def   Constr Req Tunable Trafo
## eta              numeric   -   -   0 to 1   -    TRUE     -
## gamma            numeric   -   -   0 to 5   -    TRUE     -
## max_depth        integer   -   -   1 to 5   -    TRUE     -
## min_child_weight numeric   -   -  1 to 10   -    TRUE     -
## subsample        numeric   -   - 0.5 to 1   -    TRUE     -
## colsample_bytree numeric   -   - 0.5 to 1   -    TRUE     -
## nrounds          integer   -   - 20 to 20   -    TRUE     -
```

```
## With control class: TuneControlRandom
```

```
## Imputation value: 1
```

```
## Exporting objects to slaves for mode socket: .mlr.slave.options
```

```
## Mapping in parallel: mode = socket; level = mlr.tuneParams; cpus = 16; elements = 1000.
```

```
## [Tune] Result: eta=0.211; gamma=1.44; max_depth=4; min_child_weight=2.98; subsample=0.662; colsample_bytree=0.614; nrounds=20 : mmce.test.mean=0.0586040
```

```
parallelStop()
```

```
## Stopped parallelization. All cleaned up.
```

```
tuned_xgb_pars
```

```
## Tune result:
## Op. pars: eta=0.211; gamma=1.44; max_depth=4; min_child_weight=2.98; subsample=0.662; colsample_bytree=0.614; nrounds=20
## mmce.test.mean=0.0586040
```

## Training the final tuned model

```
tuned_xgb <- setHyperPars(xgb, par.vals = tuned_xgb_pars$x)
tuned_xgb_model <- train(tuned_xgb, xgb_task)
```

```
## [10:42:46] WARNING: amalgamation/../src/learner.cc:1095: Starting in XGBoost 1.3.0, the default evaluation metric used with the objective 'binary:logistic' was changed from 'error' to 'logloss'. Explicitly set eval_metric if you'd like to restore the old behavior.
```

```
saveRDS(tuned_xgb_model, file = "tuned_xgb_model.RDS") # saving the trained model to use with the test data
```

## Plotting iteration number against log loss

```
xgb_model_data <- getLearnerModel(tuned_xgb_model)
ggplot(xgb_model_data$evaluation_log, aes(iter, 'merror')) + 
  geom_line() +
  geom_point()
```

## Drawing individual trees

```
xgboost::xgb.plot.tree(model = xgb_model_data, trees = 1:5)
```

## Cross-validating the model-building process

```
outer <- makeResampleDesc("CV", iters = 3)
xgb_wrapper <- makeTuneWrapper("classif.xgboost",
                              resampling = cv_for_tuning, 
                              par.set = xgb_param_space,
                              control = rand_search)
library(parallel)
library(parallelMap)
print(paste("XGBoost CV: Detected ", detectCores(), " cores", collapse = ""))
```

```
## [1] "XGBoost CV: Detected  16  cores"
```

```
parallelStartSocket(cpus = detectCores())
```

```
## Starting parallelization in mode=socket with cpus=16.
```

```
cv_with_tuning <- resample(xgb_wrapper, xgb_task, resampling = outer)
```

```
## Exporting objects to slaves for mode socket: .mlr.slave.options
```

```
## Resampling: cross-validation
```

```
## Measures:             mmce
```

```
## Mapping in parallel: mode = socket; level = mlr.resample; cpus = 16; elements = 3.
```

```
##
```

```
## Aggregated Result: mmce.test.mean=0.0685825
```

```
##
```

```
parallelStop()
```

```
## Stopped parallelization. All cleaned up.
```

```
cv_with_tuning
```

```
## Resample Result
## Task: mood_data_train_class_xg
## Learner: classif.xgboost.tuned
## Aggr perf: mmce.test.mean=0.0685825
## Runtime: 13880.6
```
