# Supplementary material for: Mood Disorder Detection in Adolescents by Classification Trees, Random Forests and XGBoost in Presence of Missing Data
Source: Entropy (Basel). 2021 Sep 14;23(9):1210. doi: 10.3390/e23091210 (PMC8468933; doi:10.3390/e23091210)
Supplement: Supplementary file 1 [file entropy-23-01210-s001.zip › social_AI/Questionnaire_original/9.pdf]

21. Zaznacz (otocz kółkiem) **taką odpowiedź, która opisuje Twoje samopoczucie w ciągu ostatniego tygodnia, łącznie z dniem dzisiejszym.** Proszę uwzględnij wszystkie pytania.

**Myśli i uczucia**	Wcale	Rzadko	Czasami	Często	Bardzo często	
Odczucie smutku lub przygnębienia	0	1	2	3	4	107
Gorszy nastrój	0	1	2	3	4	108
Napady płaczu lub płaczliwość	0	1	2	3	4	109
Poczucie zniechęcenia	0	1	2	3	4	110
Poczucie beznadziei	0	1	2	3	4	111
Niska samoocena	0	1	2	3	4	112
Poczucie bezwartościowości i nieadekwatności	0	1	2	3	4	113
Poczucie winy lub wstydu	0	1	2	3	4	114
Krytykowanie i obwinianie siebie	0	1	2	3	4	115
Problemy z podejmowaniem decyzji	0	1	2	3	4	116
**Związki z ludźmi i działanie**						
Utrata zainteresowania życiem rodzinnym, przyjaciółmi, znajomymi	0	1	2	3	4	117
Samotność	0	1	2	3	4	118
Spędzanie mniej czasu z rodziną lub przyjaciółmi	0	1	2	3	4	119
Utrata motywacji	0	1	2	3	4	120
Utrata zainteresowania szkołą (pracą) lub innymi działaniami	0	1	2	3	4	121
Unikanie szkoły (pracy) i innych działań	0	1	2	3	4	122
Utrata przyjemności i zadowolenia z życia	0	1	2	3	4	123
**Objawy fizyczne**						
Poczucie przemęczenia	0	1	2	3	4	124
Problemy z zasypianiem lub spanie za długo	0	1	2	3	4	125
Zmniejszone lub zwiększone łaknienie	0	1	2	3	4	126
Zamartwianie się o swoje zdrowie	0	1	2	3	4	127
**Impulsy samobójcze**						
Czy dręczą cię myśli samobójcze	0	1	2	3	4	128
Czy chciałbyś zakończyć życie?	0	1	2	3	4	129
Czy masz plan, jak zrobić sobie krzywdę	0	1	2	3	4	130

Projekt „**Życie Lubuszan. Współczesność i perspektywy**" współfinansowany przez Unię Europejską w ramach Europejskiego Funduszu Społecznego
