# Supplementary material for: Mood Disorder Detection in Adolescents by Classification Trees, Random Forests and XGBoost in Presence of Missing Data
Source: Entropy (Basel). 2021 Sep 14;23(9):1210. doi: 10.3390/e23091210 (PMC8468933; doi:10.3390/e23091210)
Supplement: Supplementary file 1 [file entropy-23-01210-s001.zip › social_AI/Questionnaire_original/8.pdf]

Sytuacja osobista

19. Biorąc pod uwagę Twoje dotychczasowe doświadczenia, możesz powiedzieć o sobie, że masz wpływ na:

	Obszar wpływu	Zawsze	Zazwyczaj	Czasami	Rzadko	Prawie wcale	Raczej nie	Zdecydowa-nie nie	
A.	Status materialny	1	2	3	4	5	6	7	85
B.	Zdrowie	1	2	3	4	5	6	7	86
C.	Oceny	1	2	3	4	5	6	7	87
D.	Przyszłe życie zawodowe	1	2	3	4	5	6	7	88
E.	Stosunki z chłopakiem/ dziewczyną	1	2	3	4	5	6	7	89
F.	Stosunki z innymi ludźmi	1	2	3	4	5	6	7	90
G.	Wygląd	1	2	3	4	5	6	7	91
H.	Stosunki z rodzicami	1	2	3	4	5	6	7	92
I.	Wolny czas	1	2	3	4	5	6	7	93

20. Zastanów się i zaznacz (otocz kółkiem właściwą cyfrę) **w jakim stopniu, poniższe cechy Cię określają:.**

	Cecha	bardzo wysokim	średnim	małym	wcale	
A.	Atrakcyjność	1	2	3	4	94
B.	Inteligencja	1	2	3	4	95
C.	Aktywność	1	2	3	4	96
D.	Towarzyskość	1	2	3	4	97
E.	Pracowitość	1	2	3	4	98
F.	Opiekuńczość	1	2	3	4	99
G.	Odpowiedzialność	1	2	3	4	100
H.	Samodzielność	1	2	3	4	101
I.	Wyrozumiałość	1	2	3	4	102
J.	Gospodarność	1	2	3	4	103
K.	Zdecydowanie	1	2	3	4	104
L.	Szczerość	1	2	3	4	105
M.	Pewność siebie	1	2	3	4	106
