# Supplementary material for: Mood Disorder Detection in Adolescents by Classification Trees, Random Forests and XGBoost in Presence of Missing Data
Source: Entropy (Basel). 2021 Sep 14;23(9):1210. doi: 10.3390/e23091210 (PMC8468933; doi:10.3390/e23091210)
Supplement: Supplementary file 1 [file entropy-23-01210-s001.zip › social_AI/Questionnaire_original/16.pdf]

Czas wolny i uczestnictwo w kulturze

42. Do jakich kół zainteresowań na terenie szkoły należysz?

1. Tak
2. Nie

Przedmiotowych kół zainteresowań (np. matematyczne, polonistyczne, historyczne)		234
Szkolnego koła sportowego		235
Sekcji tanecznej		236
Zespołu muzycznego		237
Inne (jakie)		238
Nigdzie nie należę, gdyż	1	239

43. W jakich zajęciach pozaszkolnych uczestniczysz?

1. Tak
2. Nie

Językowych (np. nauka niemieckiego, angielskiego)		240
Sportowych (np. zajęcia na basenie, sekcja sportowa)		241
Artystycznych (np. taniec, gra na instrumencie)		242
Hobbystycznych (np. modelarskie)		243
Inne (jakie?)		244

44. Czy – i gdzie – pracujesz społecznie?

1. Tak
2. Nie

W samorządzie szkolnym		245
W samorządzie klasowym		246
W organizacji charytatywnej (Wielka Orkiestra Świątecznej Pomocy, pomoc dla zwierząt, itp.)		247
W wolontariacie		248
Inne (jakie?)		249
Nigdzie nie działam, gdyż	1	250
