# Supplementary material for: Mood Disorder Detection in Adolescents by Classification Trees, Random Forests and XGBoost in Presence of Missing Data
Source: Entropy (Basel). 2021 Sep 14;23(9):1210. doi: 10.3390/e23091210 (PMC8468933; doi:10.3390/e23091210)
Supplement: Supplementary file 1 [file entropy-23-01210-s001.zip › social_AI/Questionnaire_original/17.pdf]

45. Jak spędzacie w grupie swój wolny czas? Możesz wybrać więcej niż jedną odpowiedź

1. *Tak*
2. *Nie*

Spotykamy się u kogoś z naszych w domu.. 251
Gramy na komputerze.. 252
Idziemy do galerii lub centrum handlowego.. 253
Siedzimy na klatce schodowej.. 254
Chodzimy po mieście.. 255
Gramy na boisku w piłkę.. 256

46. Jakie wartości mają w Waszej grupie największe znaczenie? Możesz wybrać więcej niż jedną odpowiedź

1. *Tak*
2. *Nie*

Wykształcenie.. 257
Praca.. 258
Zdrowie.. 259
Pieniądze.. 260
Religia.. 261
Zabawa.. 262
Aktywność społeczna.. 263
Inne, jakie?.. 264

47. Czy masz hobby?.. 265

1. *Nie*
2. *Tak, jakie?*.. [illegible]

48. Zdarza się czasem, że nie korzystasz z żadnych ofert spędzania czasu wolnego i nudzisz się. Bywa, że nuda jest trudna do zniesienia. Jak sobie z nią radzisz?

.... 266
.... 267
.... 268

[illegible] !
