# Supplementary material for: Mood Disorder Detection in Adolescents by Classification Trees, Random Forests and XGBoost in Presence of Missing Data
Source: Entropy (Basel). 2021 Sep 14;23(9):1210. doi: 10.3390/e23091210 (PMC8468933; doi:10.3390/e23091210)
Supplement: Supplementary file 1 [file entropy-23-01210-s001.zip › social_AI/Questionnaire_original/15.pdf]

39. W jakich sprawach zwracasz się o pomoc do wychowawcy klasy? (Zaznacz właściwe)

1. Tak
2. Nie

(Jeśli nie zwraca się do wychowawcy to w pkt. 218 → wpisuje 1)

Dotyczących problemów w nauce		212
Dotyczących konfliktów z nauczycielem/ nauczycielami		213
Dotyczących konfliktów z rówieśnikami		214
Dotyczących agresji i przemocy wobec Ciebie ze strony kolegów/ koleżanek		215
Dotyczących problemów w rodzinie		216
Inne		217
Nie zwracam się do wychowawcy z żadnymi problemami		218

40. W jakich sprawach zwracasz się o pomoc do pedagoga szkolnego?

1. Tak
2. Nie

Dotyczących problemów w nauce		219
Dotyczących konfliktów z nauczycielem/ nauczycielami		220
Dotyczących konfliktów z rówieśnikami		221
Dotyczących agresji i przemocy wobec Ciebie ze strony kolegów/ koleżanek		222
Dotyczących problemów w rodzinie		223
Inne		224
Nie zwracam się do pedagoga z żadnymi problemami		225

wpisuje 1

41. Kto dla Ciebie jest autorytetem? Otocz kółkiem właściwą cyfrę w odpowiedniej kratce

Osoba	Zdecydowanie tak	Raczej tak	Raczej nie	Zdecydowanie nie	
Nauczyciel	1	2	3	4	226
Rodzic	1	2	3	4	227
Inna osoba z rodziny, kto?	1	2	3	4	228
Kolega/koleżanka	1	2	3	4	229
Idol (np. aktor, sportowiec, piosenkarz	1	2	3	4	230
Ksiądz	1	2	3	4	231
Trener	1	2	3	4	232
Inne osoby, jakie?	1	2	3	4	233
