# Supplementary material for: Mood Disorder Detection in Adolescents by Classification Trees, Random Forests and XGBoost in Presence of Missing Data
Source: Entropy (Basel). 2021 Sep 14;23(9):1210. doi: 10.3390/e23091210 (PMC8468933; doi:10.3390/e23091210)
Supplement: Supplementary file 1 [file entropy-23-01210-s001.zip › social_AI/Questionnaire_original/14.pdf]

Wsparcie

34. Czy jest ktoś (osoba, osoby) do kogo możesz się zawsze zwrócić o pomoc? 197

1. Tak
2. Raczej tak
3. Raczej nie
4. Nie

35. Jeśli podkreśliłeś odpowiedź 1.lub 2., to wymień te osoby. Jeśli podkreśliłeś odpowiedź 3. lub 4. przejdź do pytania 37

1. Tak
2. Nie

Kolega/ koleżanka 198
Rodzic 199
Ksiądz 200
Trener 201
Nauczyciel, pedagog szkolny 202
Inne osoby (napisz jakie) 203

36. Czy masz osobę godną zaufania, którą możesz prosić o radę, jeśli masz jakieś problemy? 204

1. Tak
2. Raczej tak
3. Raczej nie
4. Nie, nie mam takiej osoby

37. Jeśli wybrałeś odpowiedź 1. lub 2. wymień te osoby. Jeśli podkreśliłeś odpowiedź 3. lub 4. przejdź do pytania 38

1. Tak
2. Nie

Kolega/ koleżanka 205
Rodzic 206
Ksiądz 207
Trener 208
Nauczyciel, pedagog szkolny 209
Inne osoby (napisz jakie) 210

38. Czy masz przyjaciela/przyjaciółkę? 211

1. Tak
2. Nie
