# Supplementary material for: Mood Disorder Detection in Adolescents by Classification Trees, Random Forests and XGBoost in Presence of Missing Data
Source: Entropy (Basel). 2021 Sep 14;23(9):1210. doi: 10.3390/e23091210 (PMC8468933; doi:10.3390/e23091210)
Supplement: Supplementary file 1 [file entropy-23-01210-s001.zip › social_AI/Questionnaire_original/10.pdf]

Sytuacja szkolna

22. Jakim jesteś uczniem? 131

1. Bardzo dobrym i celującym (średnia ocen powyżej 5,0)
2. Dobrym i bardzo dobrym (średnia ocen: 4.0 do 4.9)
3. Dobrym i dostatecznym (średnia ocen: 3.0 do 3.9)
4. Dostatecznym i miernym (średnia ocen poniżej 3.0)

23. Czy zdarzyło się, że powtarzałeś klasę/klasy? 132

1. Tak
2. Nie

Jeśli tak, to którą/ które? ……………………………………………………………………………………………

24. Co sprawia Ci w szkole najwięcej trudności (zaznacz nie więcej niż **trzy** odpowiedzi)

1. Zdobycie pozytywnej oceny z większości przedmiotów 133
2. Zdobycie pozytywnej oceny z niektórych przedmiotów 134
3. Stworzenie dobrych relacji z kolegami/koleżankami 135
4. Stworzenie dobrych relacji z nauczycielami
5. Zachowanie dyscypliny na lekcjach
6. Poprawne zachowanie podczas przerw i w czasie pozalekcyjnym
7. Nic mi nie sprawia trudności
8. Inne ………………………………………………………………………

25. Czy czujesz się w swojej szkole bezpieczny? 136

1. zdecydowanie tak
2. raczej tak
3. raczej nie
4. zdecydowanie nie

26. Jak oceniasz swoje relacje z rówieśnikami z klasy? 137

1. Jako bardzo dobre
2. Jako raczej dobre
3. Jako raczej złe
4. Jako zdecydowanie złe
