# Supplementary material for: Mood Disorder Detection in Adolescents by Classification Trees, Random Forests and XGBoost in Presence of Missing Data
Source: Entropy (Basel). 2021 Sep 14;23(9):1210. doi: 10.3390/e23091210 (PMC8468933; doi:10.3390/e23091210)
Supplement: Supplementary file 1 [file entropy-23-01210-s001.zip › social_AI/Questionnaire_original/11.pdf]

**27. Jak czujesz się w porównaniu do swoich rówieśników w zakresie:**

| Zakresy | Zawsze czuję się gorszy | Czasem czuję się gorszy | Jestem taki sam jak inni | Czasem czuje się lepszy | Zawsze czuję się lepszy | |
|---|---|---|---|---|---|---|
| A. Odzieży i modnych gadżetów √ | 0 | 1 | 2 | 3 | 4 | 138 |
| B. Wyglądu (wzrostu, wagi ciała itp.) | 0 | 1 | 2 | 3 | 4 | 139 |
| C. Ocen | 0 | 1 | 2 | 3 | 4 | 140 |
| D. Powodzenia u płci przeciwnej | 0 | 1 | 2 | 3 | 4 | 141 |
| E. Sprawności fizycznej | 0 | 1 | 2 | 3 | 4 | 142 |
| F. Zdolności, umiejętności | 0 | 1 | 2 | 3 | 4 | 143 |
| G. Stanu zdrowia (częste choroby) | 0 | 1 | 2 | 3 | 4 | 144 |
| H. Specyficznych trudności (jąkanie, dysleksja, nadpobudliwość) | 0 | 1 | 2 | 3 | 4 | 145 |
| I. Możliwości finansowych √ | 0 | 1 | 2 | 3 | 4 | 146 |
| J. Szans na wyjścia do kina czy na dyskoteki √ | 0 | 1 | 2 | 3 | 4 | 147 |
| K. Wyznania, religii | 0 | 1 | 2 | 3 | 4 | 148 |
| L. Narodowości | 0 | 1 | 2 | 3 | 4 | 149 |
| M. Możliwości wyjazdów na wakacje za granicę | 0 | 1 | 2 | 3 | 4 | 150 |
| N. Dobrych kosmetyków √ | 0 | 1 | 2 | 3 | 4 | 151 |
| O. Miejsca zamieszkania | 0 | 1 | 2 | 3 | 4 | 152 |
| P. Warunków mieszkaniowych √ | 0 | 1 | 2 | 3 | 4 | 153 |
| Q. Sytuacji finansowej rodziny √ | 0 | 1 | 2 | 3 | 4 | 154 |
| R. Innej sytuacji w rodzinie | 0 | 1 | 2 | 3 | 4 | 155 |
| S. Inne ... (niech wpiszę) | 0 | 1 | 2 | 3 | 4 | 156 |

## Grupy rówieśnicze

**28. Czy należysz do grupy – paczki kolegów/koleżanek?** 157

1. *Tak*
2. *Nie*

**29. Czy zdarza Ci się odczuwać osamotnienie w grupie koleżeńskiej?** 158

1. *Tak, bardzo często*
2. *Tak, czasami je odczuwam*
3. *Tak, ale bardzo rzadko*
4. *Nigdy nie odczuwam osamotnienia w relacjach z kolegami*
