# Supplementary material for: Mood Disorder Detection in Adolescents by Classification Trees, Random Forests and XGBoost in Presence of Missing Data
Source: Entropy (Basel). 2021 Sep 14;23(9):1210. doi: 10.3390/e23091210 (PMC8468933; doi:10.3390/e23091210)
Supplement: Supplementary file 1 [file entropy-23-01210-s001.zip › social_AI/Questionnaire_original/13.pdf]

Lubuskie

UNIWERSYTET ZIELONOGÓRSKI

możesz wpisać → trzy najważniejsze

31. Które z tych zachowań zdarza się Tobie podejmować pod wpływem grupy?

(wpisuje literki z pyt. 30)

a .... | 1 | 179

b .... | 2 | 180

c .... | 3 | 181

32. Czy i jak często:

Zachowania	Kilka razy w tygodniu	Kilka razy w semestrze	1-3 razy w roku szkolnym	Nie zdarzyło mi się	
Zostałeś okradziony/a	1	2	3	4	182
Zostałeś pobity przez kolegów ze szkoły	1	2	3	4	183
Zostałeś pobity przez kolegów spoza szkoły	1	2	3	4	184
Zmuszano Ciebie do przyjęcia narkotyku	1	2	3	4	185
Zmuszano Ciebie do wykonania czynności, których nie chciałeś wykonać	1	2	3	4	186
Wyzywano, przezywano Ciebie	1	2	3	4	187
Kaleczono Ciebie	1	2	3	4	188
Byłeś filmowany telefonem komórkowym i narażony na ośmieszenie (poprzez np. umieszczenie filmu w Internecie)	1	2	3	4	189
Zmuszano Cię do spożywania alkoholu	1	2	3	4	190
Odurzano Ciebie	1	2	3	4	191
Wymuszano na Tobie coś (np. pieniędzy, papierosów)	1	2	3	4	192
Zniszczono Twoją własność	1	2	3	4	193

33. Kto jest najbardziej narażony na agresję? Wybierz nie więcej niż **trzy** odpowiedzi i ponumeruj według ważności (**1** – oznacza cechę **najważniejszą**)

1 *Osoby nieśmiałe*
2 *Zaczepiające inne osoby, agresywne* 2
3 *Osoby, które są gorzej ubrane*
4 *Osoby, które dostają dobre stopnie*
5 *Osoby, które odbiegają wyglądem fizycznym od innych* 1
6 *Osoby przeszkadzające innym* 3
7 *Inne – wpisz jakie*...

ponumeruj

2	
1	
	194 195
	196

trzy pierwsze numer

→ inne odpowiedź

V wiele przy wybranej odpowiedzi wpisz numer 1-3
podkreśl odpowiedź

KAPITAŁ LUDZKI
NARODOWA STRATEGIA SPÓJNOŚCI

Projekt „**Życie Lubuszan. Współczesność i perspektywy**" współfinansowany przez Unię Europejską w ramach Europejskiego Funduszu Społecznego

UNIA EUROPEJSKA
EUROPEJSKI FUNDUSZ SPOŁECZNY
