# Supplementary material for: Mood Disorder Detection in Adolescents by Classification Trees, Random Forests and XGBoost in Presence of Missing Data
Source: Entropy (Basel). 2021 Sep 14;23(9):1210. doi: 10.3390/e23091210 (PMC8468933; doi:10.3390/e23091210)
Supplement: Supplementary file 1 [file entropy-23-01210-s001.zip › social_AI/Questionnaire_original/12.pdf]

30. Jak często zdarza Ci się:

Zachowania	Kilka razy w tygodniu	Kilka razy w semestrze	2 -3 razy w roku szkolnym	Nie zdarzyło mi się	
A. Chodzić na wagary	1	2	3	4	159
B. Uciekać z domu	1	2	3	4	160
C. Palić papierosy	1	2	3	4	161
D. Dokonywać drobnych kradzieży	1	2	3	4	162
E. Dokonywać poważnych kradzieży (zgłoszona na Policję)	1	2	3	4	163
F. Pobić kolegę/koleżankę ze szkoły	1	2	3	4	164
G. Pobić osobę spoza szkoły	1	2	3	4	165
H. Przyjmować narkotyki	1	2	3	4	166
I. Dokonywać samookaleczenia	1	2	3	4	167
J. Spożywać alkohol	1	2	3	4	168
K. Przyjmować środki odurzające	1	2	3	4	169
L. Przyjmować dopalacze	1	2	3	4	170
M. Wyzywać, przezywać kolegów/koleżanki	1	2	3	4	171
N. Wymuszać na kimś coś (np. pieniądze, papierosy)	1	2	3	4	172
O. Zniszczyć rzecz należącą do kolegi / koleżanki	1	2	3	4	173
P. Niszczyć mienie szkoły (np. ciąć ławkę, wyrywać klamkę)	1	2	3	4	174
Q. Dewastować mienie społeczne (autobusy, przystanki)	1	2	3	4	175
R. Malować graffiti	1	2	3	4	176
S. Filmować kogoś telefonem komórkowym z zamiarem ośmieszenia (poprzez np. umieszczenie filmu w Internecie)	1	2	3	4	177
T. Znęcać psychicznie nad kimś	1	2	3	4	178
