# Supplementary material for: Mood Disorder Detection in Adolescents by Classification Trees, Random Forests and XGBoost in Presence of Missing Data
Source: Entropy (Basel). 2021 Sep 14;23(9):1210. doi: 10.3390/e23091210 (PMC8468933; doi:10.3390/e23091210)
Supplement: Supplementary file 1 [file entropy-23-01210-s001.zip › social_AI/Questionnaire_original/20.pdf]

53. Czy zdarza się, że odkładasz na później naukę czy obowiązki domowe, by mieć więcej czasu na (otocz kółkiem właściwą cyfrę):

Czynność	Nigdy	Kilka razy w miesiącu	Kilka razy w tygodniu	Codziennie	
Rozmowy przez telefon komórkowy	1	2	3	4	295
Pisanie i wysyłanie sms-ów	1	2	3	4	296
Siedzenie przed komputerem	1	2	3	4	297
Korzystanie z Internetu	1	2	3	4	298
Gry playstation	1	2	3	4	299
Oglądanie telewizji	1	2	3	4	300

Metryczka

54. Czy jesteś? 301

1. *Chłopcem*
2. *Dziewczyną*

55. Jakie wykształcenie mają Twoi rodzice?

1. *Podstawowe*
2. *Zawodowe*
3. *Średnie*
4. *Wyższe*

5. Nie wiem

A) Ojciec 302

B) Matka 303

304

56. Ile dzieci, łącznie z Tobą, jest w Twojej rodzinie? ....

1. *Jedno (jesteś jedynakiem/jedynaczką)*
2. *Dwoje*
3. *Troje i więcej*

Dziękujemy za wypełnienie ankiety

KAPITAŁ LUDZKI
NARODOWA STRATEGIA SPÓJNOŚCI

Projekt „Życie Lubuszan. Współczesność i perspektywy" współfinansowany przez Unię Europejską w ramach Europejskiego Funduszu Społecznego

UNIA EUROPEJSKA
EUROPEJSKI
FUNDUSZ SPOŁECZNY
