# Supplementary material for: Mood Disorder Detection in Adolescents by Classification Trees, Random Forests and XGBoost in Presence of Missing Data
Source: Entropy (Basel). 2021 Sep 14;23(9):1210. doi: 10.3390/e23091210 (PMC8468933; doi:10.3390/e23091210)
Supplement: Supplementary file 1 [file entropy-23-01210-s001.zip › social_AI/Questionnaire_original/19.pdf]

51. Zastanów się i zaznacz ile czasu dziennie poświęcasz na (otocz kółkiem właściwą cyfrę)

Czynność	W dni powszednie				W dni wolne od nauki				
	W ogóle nie korzystam	Poniżej 2 godzin	Od 2 do 4 godz	Powyżej 4 godz.	Poniżej 2 godz.	Od 2 do 4 godz	Od 4 do 6 godz.	Powyżej 6 godz.	
Rozmowy telefoniczne	1	2	3	4	1	2	3	4	279
Sms-y (wysyłanie i czytanie)	1	2	3	4	1	2	3	4	280
Gry komputerowe	1	2	3	4	1	2	3	4	281
Korzystanie z komunikatorów w Internecie (np. GG)	1	2	3	4	1	2	3	4	282
Prowadzenie własnego bloga	1	2	3	4	1	2	3	4	283
Poszukiwanie nowych znajomości	1	2	3	4	1	2	3	4	284
Oglądanie filmów z internetu	1	2	3	4	1	2	3	4	285
Czytanie wiadomości politycznych i społecznych	1	2	3	4	1	2	3	4	286
Oglądanie telewizji	1	2	3	4	1	2	3	4	287
Gry playstation	1	2	3	4	1	2	3	4	288

52. Jak często Twoi rodzice narzekają na to, że zbyt dużo czasu przeznaczasz na (otocz kółkiem właściwą cyfrę):

Czynność	Nigdy	Kilka razy w miesiącu	Kilka razy w tygodniu	Codziennie	
Rozmowy przez telefon komórkowy	1	2	3	4	289
Pisanie i wysyłanie sms-ów	1	2	3	4	290
Siedzenie przed komputerem	1	2	3	4	291
Korzystanie z Internetu	1	2	3	4	292
Gry playstation	1	2	3	4	293
Oglądanie telewizji	1	2	3	4	294
