# Supplementary material for: Mood Disorder Detection in Adolescents by Classification Trees, Random Forests and XGBoost in Presence of Missing Data
Source: Entropy (Basel). 2021 Sep 14;23(9):1210. doi: 10.3390/e23091210 (PMC8468933; doi:10.3390/e23091210)
Supplement: Supplementary file 1 [file entropy-23-01210-s001.zip › social_AI/Questionnaire_original/18.pdf]

49. Jak często uczestniczysz w różnych formach kultury?(otocz kółkiem właściwą cyfrę)

Kategorie	Przynajmniej raz w tygodniu	Przynajmniej raz w miesiącu	Kilka razy w roku	Rzadziej	Nigdy nie byłem	
Kino	1	2	3	4	5	269
Teatr	1	2	3	4	5	270
Filharmonia	1	2	3	4	5	271
Wystawa	1	2	3	4	5	272
Muzeum	1	2	3	4	5	273

Uzależnienie

50. Czy masz dostęp do (otocz kółkiem właściwącyfrę)

Urządzenia	Tak – mam własny	Tak – mamy w domu do wspólnego użytku	Nie mam stałego dostępu	Korzystam w domu kolegi	
Telefon komórkowy	1	2	3	4	274
Komputer	1	2	3	4	275
Internet	1	2	3	4	276
Play station	1	2	3	4	277
Telewizor	1	2	3	4	278
