# Supplementary material for: Mood Disorder Detection in Adolescents by Classification Trees, Random Forests and XGBoost in Presence of Missing Data
Source: Entropy (Basel). 2021 Sep 14;23(9):1210. doi: 10.3390/e23091210 (PMC8468933; doi:10.3390/e23091210)
Supplement: Supplementary file 1 [file entropy-23-01210-s001.zip › social_AI/Questionnaire_original/6.pdf]

Lubuskie

UNIWERSYTET ZIELONOGÓRSKI

14. Młodzi ludzie czasem odczuwają osamotnienie w rodzinie. Co najczęściej robisz, gdy zdarza Ci się poczuć samotnym w domu? Możesz dokonać wyboru trzech odpowiedzi.

1. Słucham muzyki 59
2. Oglądam telewizję
3. Gram w gry komputerowe 60
4. Surfuję po Internecie
5. Rozmawiam ze znajomymi na GG, czacie 61
6. Piszę co myślę na własnym blogu
7. Wychodzę z domu i spaceruję
8. Wychodzę z domu i spotykam się z kolegami/koleżankami
9. Sięgam po alkohol
10. Sięgam po narkotyki
11. Sięgam po papierosy
12. Inne, jakie ....
13. Nic nie robię
14. Nigdy nie odczułem osamotnienia w rodzinie

15. Czy jest coś co chciałbyś zmienić w swojej rodzinie? Wpisz krótką wypowiedź.

Kody
1. Nic
2. Więcej czasu z rodz.

.... 62

.... 63

.... 64

16. Kiedy czujemy się samotni często towarzyszą temu inne stany, emocje i myśli. Oceń jak bardzo Ty ich doświadczasz

	Prawie nie czuję 1	2	3	4	5	6	Odczuwam bardzo silnie 7	
a) Czuję się bezradny	1	2	3	4	5	6	7	65
b) Odczuwam żal	1	2	3	4	5	6	7	66
c) Czuję obojętność	1	2	3	4	5	6	7	67
d) Jestem przygnębiony	1	2	3	4	5	6	7	68
e) Mam złe myśli	1	2	3	4	5	6	7	69
f) Inne, jakie	1	2	3	4	5	6	7	70
g) Nigdy nie odczułem	1	2	3	4	5	6	7	71

Jeśli nie mogą tych wymienionych uczuć -> to zaznacza g 1

17. Czy rozmawiałeś z kimś o swoim poczuciu osamotnienia? 72

1. *Nie, choć to czasem przeżywam, z nikim o tym nie rozmawiam*
2. *Tak, z kim?* ..... mają wymienić
