# Supplementary material for: Mood Disorder Detection in Adolescents by Classification Trees, Random Forests and XGBoost in Presence of Missing Data
Source: Entropy (Basel). 2021 Sep 14;23(9):1210. doi: 10.3390/e23091210 (PMC8468933; doi:10.3390/e23091210)
Supplement: Supplementary file 1 [file entropy-23-01210-s001.zip › social_AI/Questionnaire_original/7.pdf]

18. Czy w ciągu ostatniego miesiąca:

	Zachowania	Nie, nigdy	Tak, jeden raz	Tak, więcej niż raz	
A.	Twoi rodzice krzyczeli na Ciebie	1	2	3	73
B.	Usłyszałeś/aś od rodziców obraźliwe słowa?	1	2	3	74
C.	Zostałeś/aś spoliczkowany/a przez rodziców?	1	2	3	75
D.	Zostałeś/aś zbity/a przez rodziców?	1	2	3	76
E.	W Twoim domu dochodziło do awantur, w których rodzice krzyczeli i wyzywali się nawzajem?	1	2	3	77
F.	Rodzice mówili, że jesteś nic nie wart/warta i nic z Ciebie nie będzie?	1	2	3	78
G.	Było Ci bardzo źle w Twoim domu?	1	2	3	79
H.	Uciekałeś/aś ze swojego domu z lęku przed pobiciem?	1	2	3	80
I.	Bałeś/aś się swoich rodziców?	1	2	3	81
J.	Rodzice zabraniali Ci za karę jedzenia lub spania?	1	2	3	82
K.	Zdarzyło się, że ktoś z rodziny stosował wobec Ciebie tzw. zły dotyk (dotykał części intymnych)	1	2	3	83
L.	Zdarzyła się interwencja Policji z powodu kłótni czy awantur?	1	2	3	84
