# Supplementary material for: Mood Disorder Detection in Adolescents by Classification Trees, Random Forests and XGBoost in Presence of Missing Data
Source: Entropy (Basel). 2021 Sep 14;23(9):1210. doi: 10.3390/e23091210 (PMC8468933; doi:10.3390/e23091210)
Supplement: Supplementary file 1 [file entropy-23-01210-s001.zip › social_AI/Questionnaire_original/5.pdf]

Mar.

13. **Proszę, odnieś każde twierdzenie do siebie i swoich relacji z rodzicami, a następnie dokonaj oceny jego prawdziwości** (poprzez otoczenie kółkiem cyfry w odpowiedniej kratce)

kółkiem zaznacz odpowiedź

Oceniane kategorie	Całkowicie prawdziwe	Raczej prawdziwe	Trudno powiedzieć	Raczej nieprawdz.	Całkowicie nieprawdz.	
A. Odczuwam, że rodzice są ze mnie zadowoleni	1	2	3	4	5	49
B. Czasem mam wrażenie, że rodzice mnie nie zauważają	1	2	3	4	5	50
C. Czuję, że rodzice w małym stopniu interesują się mną	1	2	3	4	5	51
D. W kłopotach mogę liczyć na radę i pomoc rodziców	1	2	3	4	5	52
E. Czasem zastanawiam się, czy rodzice naprawdę mnie kochają	1	2	3	4	5	53
F. Rodzice poświęcają mi mało czasu	1	2	3	4	5	54
G. Odczuwam, że jestem bliski swoim rodzicom	1	2	3	4	5	55
H. Trudno mi rozmawiać z rodzicami	1	2	3	4	5	56
I. Odczuwam, ze jestem dla rodziców ważną osobą	1	2	3	4	5	57
J. Mam wrażenie, ze rodzice mnie nie rozumieją	1	2	3	4	5	58
