# Supplementary material for: Mood Disorder Detection in Adolescents by Classification Trees, Random Forests and XGBoost in Presence of Missing Data
Source: Entropy (Basel). 2021 Sep 14;23(9):1210. doi: 10.3390/e23091210 (PMC8468933; doi:10.3390/e23091210)
Supplement: Supplementary file 1 [file entropy-23-01210-s001.zip › social_AI/Questionnaire_original/4.pdf]

D. Podejmuję pracę 35

E. Nie muszę pomagać, bo nie ma większych trosk 36

F. Inne 37

10. Jeśli pokreśliłeś odpowiedź D w pytaniu 9 wpisz jakie prace wykonujesz?
Jeśli dokonałeś/aś innego wyboru przejdź do pytania 13

1. Tak
2. Nie

A. Pracuję na godziny (np. pomoc w sprzątaniu, pilnowanie dzieci innych osób, przenoszeniu towaru i in.) 38

B. roznoszę ulotki 39

C. pracuję w promocji (np. reklamujesz towary w sklepie) 40

D. proszę innych o pieniądze na podręczniki czy jedzenie 41

E. Zbieram płody leśne (jagody, grzyby, jeżyny, drzewo) 42

F. Sprzedaję (płody leśne, kwiaty, owoce) 43

G. Biorę udział w pracach sezonowych (żniwa, sianokosy, wykopki i in.) 44

H. Myję szyby w samochodach, pracuję na parkingu 45

I. Inne 46

11. Z czyjej inicjatywy podejmujesz wyżej wymienioną pracę?... 47

1. wymagają tego ode mnie rodzice
2. czuję się w obowiązku wspomóc finansowo rodzinę
3. z własnej inicjatywy w celu zwiększenia swojego kieszonkowego
4. z inicjatywy kolegów i koleżanek
5. inne (jakie)..

12. Jeśli podkreśliłeś odpowiedź D) w pytaniu 9, określ na co przeznaczasz zarobione pieniądze? 48

1. w całości oddaję rodzicom
2. część oddaję rodzicom, część pozostawiam na swoje potrzeby
3. w całości na swoje utrzymanie np. odzież, bilety, jedzenie
4. w całości na swoje przyjemności np. używki, zabawa, kino
5. na swoje utrzymanie i swoje przyjemności
