# Supplementary material for: Mood Disorder Detection in Adolescents by Classification Trees, Random Forests and XGBoost in Presence of Missing Data
Source: Entropy (Basel). 2021 Sep 14;23(9):1210. doi: 10.3390/e23091210 (PMC8468933; doi:10.3390/e23091210)
Supplement: Supplementary file 1 [file entropy-23-01210-s001.zip › social_AI/Questionnaire_original/1.pdf]

**Nr ankiety**					1

Powiat	....		2
Miejscowość	....		3
Szkoła	....		4
Klasa	....		5

Gimnazjalisto!

Żyjesz w bardzo ciekawych czasach. Świat stwarza Tobie niezliczone możliwości; zdobycze współczesności sprawiają, że masz szansę na własny rozwój, życie pożyteczne i przyjemne. Jednak rzeczywistość to także wiele zagrożeń i trudności. Żyjesz w świecie, w którym tak łatwo się zagubić, być nieszczęśliwym.

Proszę wypełnij kwestionariusz ankiety. Uzyskane wyniki pozwolą na poznanie Ciebie, Twoich obaw i problemów – Twojego miejsca w otaczającej rzeczywistości. Masz szansę wypowiedzieć się i być wysłuchanym – skorzystaj z tego, Może sprawisz, że podjęte zostaną działania, które pozwolą Tobie i Tobie podobnym dobrze poczuć się ze sobą samym, w rodzinie, szkole, wśród rówieśników.

Ankieta jest anonimowa. Proszę bądź szczery w swych wypowiedziach.

1. W jakiej szkole planujesz kontynuować naukę po skończeniu gimnazjum? [ ] 6

1. *W liceum ogólnokształcącym* (każde liceum)
2. *W technikum*
3. *W zasadniczej szkole zawodowej*
4. *W ośrodku doskonalenia zawodowego, w którym zdobędę uprawnienia do wykonywania zawodu*
5. *Nie planuję dalej uczyć się*

2. Jak sądzisz, czy uda Ci się zrealizować te plany? [ ] 7

1. *Tak*
2. *Raczej tak*
3. *Raczej nie*
4. *Nie*
