# Supplementary material for: Mood Disorder Detection in Adolescents by Classification Trees, Random Forests and XGBoost in Presence of Missing Data
Source: Entropy (Basel). 2021 Sep 14;23(9):1210. doi: 10.3390/e23091210 (PMC8468933; doi:10.3390/e23091210)
Supplement: Supplementary file 1 [file entropy-23-01210-s001.zip › social_AI/Questionnaire_original/3.pdf]

4. W moim domu często brakuje pieniędzy na jedzenie, opłaty czy ubranie; często zapożyczamy się, ale wszystkie długi oddajemy

5. W moim domu bardzo często brakuje pieniędzy na jedzenie, opłaty czy ubranie; ale pomaga nam pomoc społeczna a także inni życzliwi ludzie

8. Jakie są główne troski Twojej rodziny w ostatnim czasie? (możesz dokonać wyboru kilku odpowiedzi)

1. Tak
2. Nie

A. Brak pracy dla obojga rodziców 19

B. Brak pracy dla jednego z rodziców 20

C. Niskie zarobki rodziców/rodzica 21

D. Konflikty w rodzinie 22

E. Problem z częstym spożywaniem alkoholu przez (wpisz kogo?) 23

F. Zadłużenie, konieczność spłaty kredytów 24

G. Zła sytuacja mieszkaniowa 25

H. Brak pieniędzy na podstawowe potrzeby rodziny (jedzenie, odzież, podręczniki i pomoce szkolne) 26

I. Rodzice (lub jedno z nich) bardzo dużo pracuje 27

J. Prawie nigdy nie spędzamy czasu wolnego 28

K. Opieka na osobą chorą albo niepełnosprawną 29

L. W ostatnim czasie nie ma w mojej rodzinie żadnych trosk 30

9. Czy pomagasz rodzinie w rozwiązywaniu Jej trosk i problemów? Jeśli tak, to w jaki sposób? Możesz dokonać wyboru kilku odpowiedzi.

1. Tak
2. Nie

31

A. Staram się oszczędzać, rezygnuję z własnych przyjemności, żeby rodzice nie musieli wydawać na mnie pieniędzy 32

B. Pomagam w domu, żeby rodzice mogli więcej pracować poza domem 33

C. Pomagam rodzicom w pracy zawodowej (np. w prowadzeniu gospodarstwa rolnego, w warsztacie, sklepie i in.) 34
