# Supplementary material for: Mood Disorder Detection in Adolescents by Classification Trees, Random Forests and XGBoost in Presence of Missing Data
Source: Entropy (Basel). 2021 Sep 14;23(9):1210. doi: 10.3390/e23091210 (PMC8468933; doi:10.3390/e23091210)
Supplement: Supplementary file 1 [file entropy-23-01210-s001.zip › social_AI/Questionnaire_original/2.pdf]

3. Jeśli masz obawy dotyczące dalszej edukacji to z czym są one związane? (możesz wybrać więcej niż jedną odpowiedź)

1. Tak
2. Nie

Nie wiem czy zostanę przyjęty do wybranej szkoły z powodu wyników egzaminu gimnazjalnego 8
Nie wiem czy rodzice zgodzą się na naukę w wybranej przeze mnie szkole 9
Nie wiem czy moi rodzice będą w stanie finansować dalszą naukę 10
Przypuszczam, że będę musiał/a podjąć pracę, żeby pomóc rodzinie 11
Nie mam żadnych obaw 12
Inne przyczyny 13

4. Jaki zawód chciałbyś wykonywać w przyszłości 14

1......... Chciał(a)bym, aby był to zawód (tu mają wpisać konkretny zawód)
2. Jeszcze nie zastanawiałem/am się nad tym

Sytuacja rodziny

5. Jaka jest obecnie Twoja sytuacja rodzinna? (Wybierz właściwą odpowiedź) 15

1. Jestem wychowywany przez oboje rodziców
2. Jestem wychowywany/a tylko przez matkę
3. Jestem wychowywany/a tylko przez ojca
4. Jestem wychowywany przez inne osoby z rodziny
5. Wychowuję się poza rodziną

6. Jaka jest sytuacja zawodowa Twoich rodziców?

1. pracuje w kraju
2. pracuje za granicą
3. nie pracuje w ogóle

MATKI 16
OJCA 17

7. Jak oceniasz sytuację ekonomiczną swojej rodziny (wybierz **jedną** odpowiedź) 18

1. W moim domu jest wszystko; rodzice mogą pozwolić sobie na realizację zachcianek i przyjemności wszystkich członków rodziny; nigdy niczego nie brakuje
2. W moim domu jest wszystko ale żyjemy skromnie, rodzice żyją „z ołówkiem w ręku", żeby na wszystko starczyło; czasami rodzice dają mi pieniądze na drobne przyjemności
3. W moim domu czasami brakuje pieniędzy na jedzenie, opłaty czy ubrania; jest bardzo skromnie, nie ma pieniędzy na przyjemności, ale jakoś sobie radzimy

KAPITAŁ LUDZKI
NARODOWA STRATEGIA SPÓJNOŚCI

Projekt „**Życie Lubuszan. Współczesność i perspektywy**" współfinansowany przez Unię Europejską w ramach Europejskiego Funduszu Społecznego

UNIA EUROPEJSKA
EUROPEJSKI FUNDUSZ SPOŁECZNY
